# Rapid construction of tricyclic tetrahydrocyclopenta[4,5]pyrrolo[2,3-*b*]pyridine via isocyanide-based multicomponent reaction

**DOI:** 10.3762/bjoc.20.126

**Published:** 2024-06-28

**Authors:** Xiu-Yu Chen, Ying Han, Jing Sun, Chao-Guo Yan

**Affiliations:** 1 College of Chemistry & Chemical Engineering, Yangzhou University, Jiangsu, Yangzhou 225002, China

**Keywords:** [3 + 2] cycloaddition, cyclopenta-1,3-diene, cyclopenta[4,5]pyrrolo[2,3-*b*]pyridine, 1,4-dihydropyridine, electron-deficient alkyne, isocyanide

## Abstract

An efficient protocol for the synthesis of polyfunctionalized tetrahydrocyclopenta[4,5]pyrrolo[2,3-*b*]pyridine-3,4b,5,6,7(1*H*)-pentacarboxylates was developed by a three-component reaction. In the absence of any catalyst, the three-component reaction of alkyl isocyanides, dialkyl but-2-ynedioates and 5,6-unsubstituted 1,4-dihydropyridines in refluxing acetonitrile afforded polyfunctionalized tetrahydrocyclopenta[4,5]pyrrolo[2,3-*b*]pyridine-3,4b,5,6,7(1*H*)-pentacarboxylates in high yields and with high diastereoselectivity. The reaction was finished by in situ generation of activated 5-(alkylimino)cyclopenta-1,3-dienes from addition of alkyl isocyanide to two molecules of but-2-ynedioates and sequential formal [3 + 2] cycloaddition reaction with 5,6-unsubstituted 1,4-dihydropyridine.

## Introduction

Isocyanide is a unique and attractive functional group in organic chemistry. The carbon atom of isocyanide has both a lone electron pair and empty orbitals, so it has outstanding electrophilic and nucleophilic reactivity. At the same time, isocyanide also has good coordination ability to coordinate with metals to form diverse metal complexes [1–3]. Therefore, isocyanides have been known as indispensable building blocks in modern organic chemistry. Many isocyanide-based carbon–carbon and carbon–heteroatom bond forming reactions have been developed in fascinating ways over the past decades [4–6]. The famous multicomponent reactions such as Passerini reaction, Ugi reaction, Orru reaction and Van Leusen reaction, in which isocyanides were employed as key substrates have become the most powerful tools for rapid construction of various nitrogen-containing organic compounds [7–14]. On the other hand, the reactive Huisgen’ 1,4-dipoles can be in situ generated by addition reaction of isocyanides to electron-deficient alkynes, which were sequentially trapped by various electrophiles and nucleophiles to give versatile acyclic and heterocyclic compounds [15–26]. In recent years, many multicomponent reactions based on alkyl isocyanides, electron-deficient alkynes and other reagents have been successfully developed for the synthesis of various carbocyclic and heterocyclic compounds [27–35].

The 5,6-unsubstituted 1,4-dihydropyridine is one of special kinds of 1,4-dihydropyridines. It can act as an activated enamino unit and electron-rich dienophile to take part in some synthetic reactions [36–39]. In recent years, 5,6-unsubstituted 1,4-dihydropyridines have been recognized as the reactive electron-rich dienophiles, which proceeded several Povarov reactions with various 1-aza-1,3-butadienes [40–44]. Recently, we have found that the three-component reaction of isoquinolines, dialkyl but-2-ynediaotes and 5,6-unsubstituted 1,4-dihydropyridines afforded functionalized isoquinolino[1,2-*f*][1,6]naphthyridines in good yields and with high diastereoselectivity via a [4 + 2] cycloaddition process [45]. Very recently, we also found that base-promoted [4 + 2] cycloaddition of salicyl *N*-tosylimines and 5,6-unsubstituted 1,4-dihydropyridines resulted in novel tetrahydrochromeno[3,2-*b*]pyridine derivatives in satisfactory yields [46]. Inspired by these efficient synthetic protocols and in continuation of our aim to develop isocyanide-based multicomponent reactions for construction of diverse nitrogen-containing heterocyclic compounds [47–58], herein we wish to report the mutlicomponent reaction of alkyl isocyanides, dialkyl but-2-ynedioates and 5,6-unsubstituted dihydropyridines for the efficient synthesis of polyfunctionalized tetrahydrocyclopenta[4,5]pyrrolo[2,3-*b*]pyridine-3,4b,5,6,7(1*H*)-pentacarboxylates.

## Results and Discussion

Initially, the reaction conditions were examined by employing cyclohexyl isocyanide (**1a**), dimethyl but-2-ynedioate (**2a**) and 5,6-unsubstituted dihydropyridine **3a** as standard reaction. The main results are summarized in Table 1. The expected product was not observed when the three-component reaction was carried out in methanol, ethanol or tetrahydrofuran at room temperature (Table 1, entries 1–3). The reaction in toluene, methylene dichloride or acetonitrile at room temperature afforded an unexpected tricyclic compound **4a** in 12–18% yields (Table 1, entries 4–6). ^1^H NMR spectra clearly indicated that two molecules of dimethyl but-2-ynedioates took part in the reaction. The yields of the product **4a** slightly increased to 29–45% yields when the reaction was carried out at elevated temperature in toluene, methylene dichloride or acetonitrile (Table 1, entries 7–10). When the reaction was carried out in refluxing acetonitrile, the tricyclic compound **4a** can be obtained in 47% yield (Table 1, entry 11). Then, the stoichiometry of dimethyl but-2-ynedioate was examined (Table 1, entries 12–15). The highest yield of **4a** (89%) was obtained by employing five equiv of dimethyl but-2-ynedioate in the reaction (Table 1, entry 15). It can be found that the reaction can be finished in less than one hour. In the presence of DABCO as base catalyst, the yield of **4a** decreased to 27% (Table 1, entry 16). Other common bases such as Et_3_N and DMAP were also employed in the reaction, they did no gave the product **4a** in higher yields than that in the absence of any base, which showed that the reaction does not need any base promotor (Table 1, entry 17 and 18). It was also found that the yield of product **4a** cannot be increased when the reaction time was prolonged to three hours (Table 1, entry 19). Thus, the optimized reaction conditions for this multicomponent reaction were successfully established.

**Table 1:** Optimizing reaction conditions^a^.

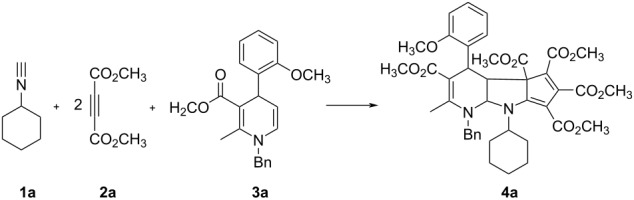						

Entry	Base	Ratio of **1a/2a/3a**	Solvent	Temp (°C)	Time (h)	Yield (%)^b^

1		1.5:3:1	MeOH	rt	6	–
2		1.5:3:1	EtOH	rt	6	–
3		1.5:3:1	THF	rt	6	–
4		1.5:3:1	PhMe	rt	1	12
5		1.5:3:1	CH_2_Cl_2_	rt	1	13
6		1.5:3:1	MeCN	rt	1	18
7		1.5:3:1	CH_2_Cl_2_	reflux	1	29
8		1.5:3:1	PhMe	reflux	1	45
9		1.5:3:1	MeCN	40 °C	1	27
10		1.5:3:1	MeCN	60 °C	1	40
11		1.5:3:1	MeCN	reflux	1	47
12		1:2:1	MeCN	reflux	1	52
13		1:3:1	MeCN	reflux	1	62
14		1:4:1	MeCN	reflux	1	71
**15**		**1:5:1**	**MeCN**	**reflux**	1	**89**
16	DABCO	1:5:1	MeCN	reflux	1	27
17	Et_3_N	1:5:1	MeCN	reflux	1	70
18	DMAP	1:5:1	MeCN	reflux	1	39
19		1:5:1	MeCN	reflux	3	87

^a^Reaction conditions: cyclohexyl isocyanide (0.1 mmol), dialkyl but-2-ynedioate, 1,4-dihydropyridine, acetonitrile (5.0 mL); ^b^isolated yields.

**Figure 1:**
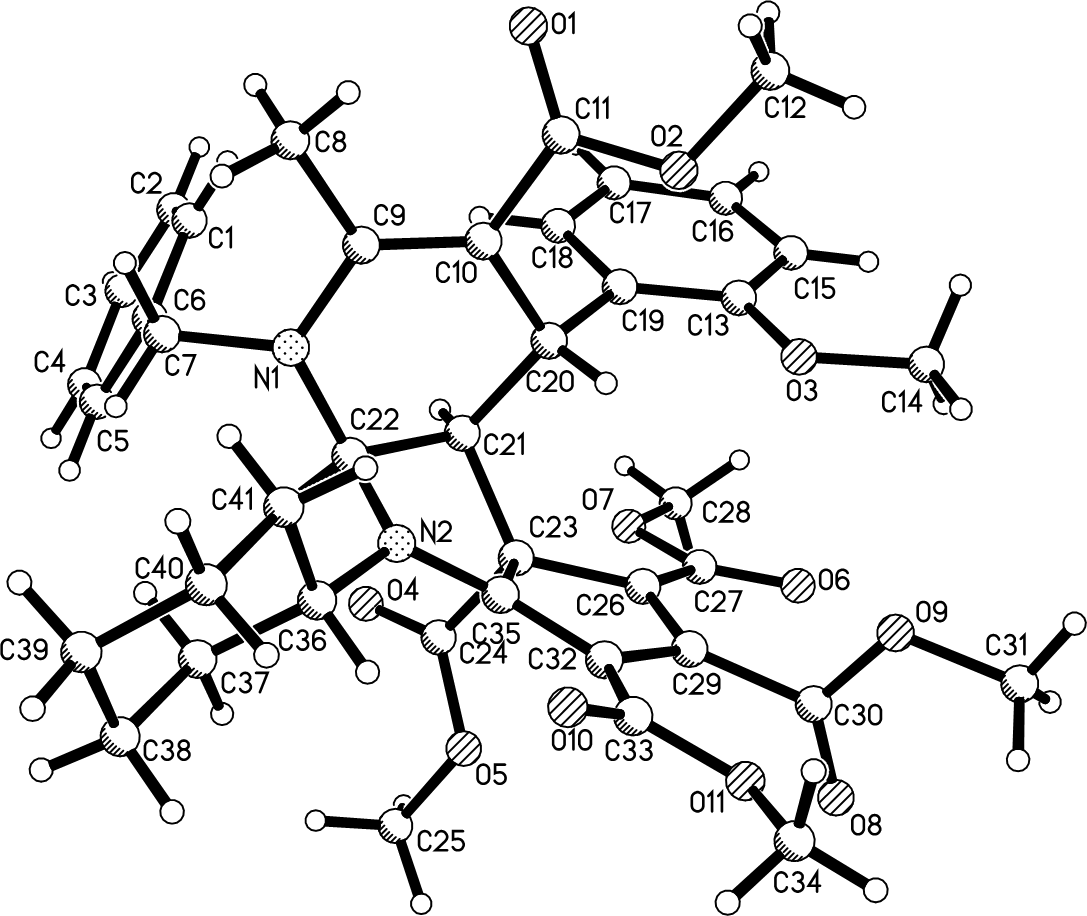
Molecular structure of compound **4a**.

Under the optimized reaction conditions, the scope of the reaction was developed by using various substrates. The results are summarized in Table 2. At first, several alkyl isocyanides such as cyclohexyl, *tert*-butyl and benzyl isocyanide have been successfully employed in the reaction. Dimethyl but-2-ynedioate usually gave the expected tricyclic products in good yields. However, the reaction with diethyl but-2-ynedioate afforded products **4p**, **4r** and **4t** in moderate to lower yields. The 5,6- unsubstituted dihydropyridines with various substituents showed marginal effects on the yields. These results clearly showed that this reaction has a wide scope of substrates. The obtained compounds **4a–t** have four chiral carbon atoms. The multicomponent reaction might result in several diastereomers. On the basis of TLC analysis and ^1^H NMR spectra of the crude products, only one relative stereochemistry was produced in the reaction, while the other diastereomers were not detected. In order to elucidate the relative configuration of the obtained compounds, the molecular structure of the compound **4a** was determined by single crystal X-ray diffraction (Figure 1). From Figure 1, it can be seen that the fused dihydropyridine ring connects with the pyrrolidine ring in *cis*-position. The 4-aryl group exists on the *trans*-position to the 2,3-pyrrolidine ring. The methoxycarbonyl group in the ring of the cyclopentadiene stretches to the *cis*-position of the 4-aryl group in the dihydropyridine ring. Thus, it can be assigned that all tricyclic compounds have this kind of relative configuration on the basis of NMR spectra and single crystal structure.

**Table 2:** The synthesis of the tricyclic compounds **4a–t**^a^.

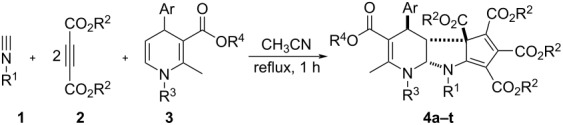							

Entry	Compound	R^1^	R^2^	Ar	R^3^	R^4^	Yield (%)^b^

1	**4a**	cyclohexyl	CH_3_	*o*-CH_3_OC_6_H_4_	Bn	CH_3_	89
2	**4b**	cyclohexyl	CH_3_	*p*-NO_2_C_6_H_4_	Bn	CH_3_	84
3	**4c**	cyclohexyl	CH_3_	*p*-CH_3_C_6_H_4_	Bn	CH_3_	81
4	**4d**	cyclohexyl	CH_3_	*o*-CH_3_OC_6_H_4_	*p*-CH_3_OC_6_H_4_CH_2_	CH_3_	84
5	**4e**	cyclohexyl	CH_3_	C_6_H_5_	*p*-ClC_6_H_4_CH_2_	CH_3_	82
6	**4f**	cyclohexyl	CH_3_	C_6_H_5_	Bn	CH_3_	90
7	**4g**	cyclohexyl	CH_3_	C_6_H_5_	*o*-CH_3_C_6_H_4_CH_2_	CH_3_	81
8	**4h**	cyclohexyl	CH_3_	C_6_H_5_	*o*-ClC_6_H_4_CH_2_	CH_3_	80
9	**4i**	cyclohexyl	CH_3_	C_6_H_5_	*m*-CH_3_OC_6_H_4_CH_2_	CH_3_	80
10	**4j**	cyclohexyl	CH_3_	C_6_H_5_	*p*-CH_3_OC_6_H_4_	CH_3_	87
11	**4k**	cyclohexyl	CH_3_	C_6_H_5_	*p*-BrC_6_H_4_	CH_3_	93
12	**4l**	cyclohexyl	CH_3_	C_6_H_5_	*m*-ClC_6_H_4_	CH_3_	92
13	**4m**	cyclohexyl	CH_3_	*o*-CH_3_OC_6_H_4_	*o*-CH_3_C_6_H_4_	CH_3_	93
14	**4n**	cyclohexyl	CH_3_	C_6_H_5_	*n*-Bu	CH_3_	80
15	**4o**	cyclohexyl	CH_3_	C_6_H_5_	Bn	CH_2_CH_3_	79
16	**4p**	cyclohexyl	CH_2_CH_3_	C_6_H_5_	Bn	CH_3_	72
17	**4q**	*t-*Bu	CH_3_	C_6_H_5_	Bn	CH_3_	66
18	**4r**	*t-*Bu	CH_2_CH_3_	C_6_H_5_	Bn	CH_3_	58
19	**4s**	Bn	CH_3_	C_6_H_5_	Bn	CH_3_	54
20	**4t**	Bn	CH_2_CH_3_	C_6_H_5_	Bn	CH_3_	30

^a^Reaction conditions: cyclohexyl isocyanide (0.1 mmol), dialkyl but-2-ynedioate (0.5 mmol), 1,4-dihydropyridine (0.1 mmol), acetonitrile (5.0 mL), reflux, 1 h; ^b^isolated yields.

**Figure 2:**
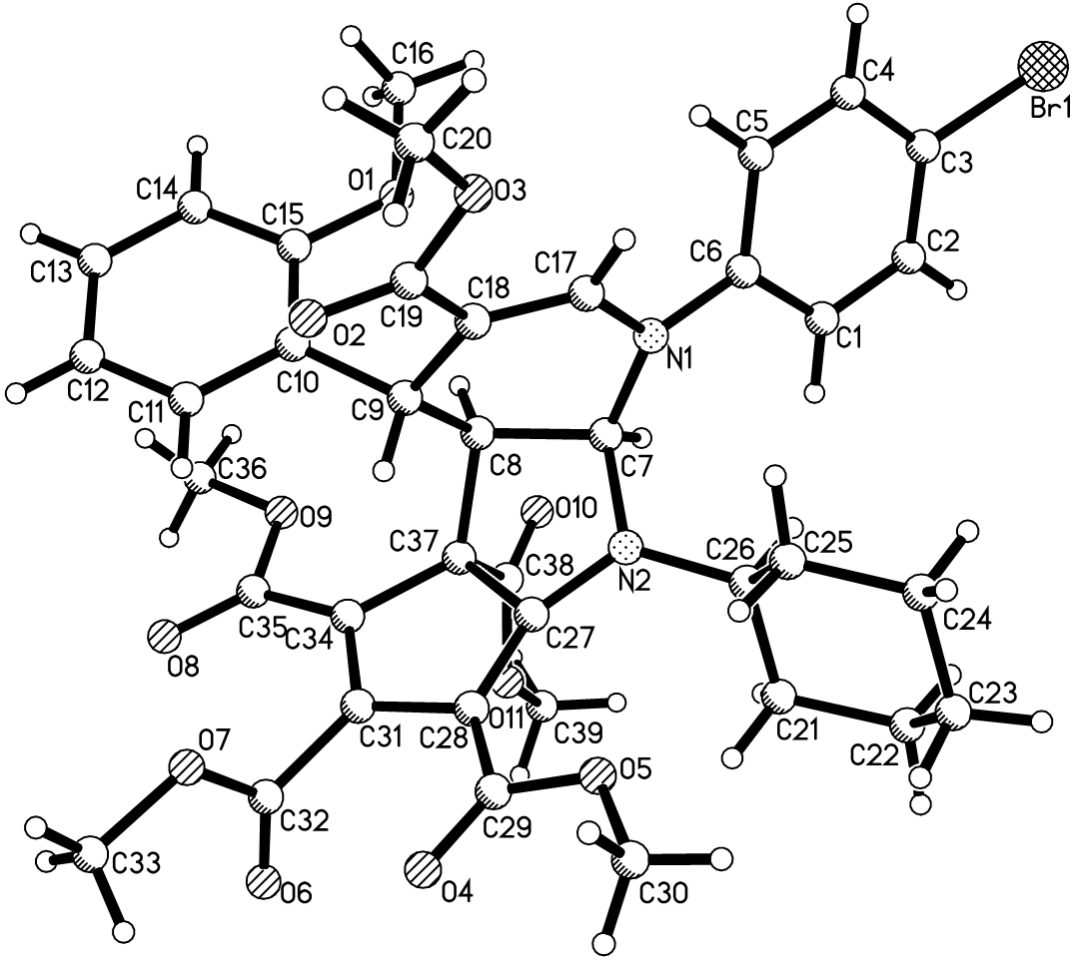
Molecular structure of compound **6g**.

In order to develop the scope of the reaction, another kind of 5,6-unsubstituted 1,4-dihydropyridines **5** were also employed in the reaction, which were previously prepared from the three-component reaction of methyl propiolate, cinnamaldehyde and arylamines. The results are summarized in Table 3. It should be pointed out that TLC analysis and ^1^H NMR spectra of the crude products usually indicated that only one diastereomer was predominately produced in the reaction even though there are four chiral carbon atoms in the products. It can be found that all reactions proceeded smoothly to give the expected polycyclic compounds **6a–k** in satisfactory yields. The substituents on the three components showed very little effect on the yields. These results showed that this reaction can be performed with a wide variety of substrates. The molecular structure of the compound **6g** was determined by single crystal X-ray diffraction method (Figure 2). The *o*-methoxyphenyl group exists on the *trans*-position of the fused pyrrolidine unit. The methoxycarbonyl group also exists on the *cis*-position of the *o*-methoxyphenyl group. Therefore, compound **6g** has the same relative configuration to that of the above mentioned product **3a**, which also indicated that this reaction has same steric controlling effect.

**Table 3:** The synthesis of the tricyclic compounds **6a–k^a^**.

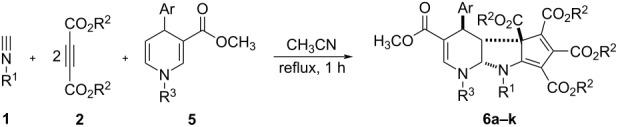						

Entry	Compd	R^1^	R^2^	Ar	R^3^	Yield (%)^b^

1	**6a**	cyclohexyl	CH_3_	C_6_H_5_	Bn	92
2	**6b**	cyclohexyl	CH_3_	C_6_H_5_	*m*-CH_3_OC_6_H_4_CH_2_	82
3	**6c**	cyclohexyl	CH_3_	C_6_H_5_	*o*-CH_3_C_6_H_4_CH_2_	83
4	**6d**	cyclohexyl	CH_3_	C_6_H_5_	*p*-CH_3_C_6_H_4_	83
5	**6e**	cyclohexyl	CH_3_	C_6_H_5_	*m*-ClC_6_H_4_	84
6	**6f**	cyclohexyl	CH_3_	C_6_H_5_	*o*-CH_3_C_6_H_4_	81
7	**6g**	cyclohexyl	CH_3_	*o*-CH_3_OC_6_H_4_	*p*-BrC_6_H_4_	88
8	**6h**	cyclohexyl	CH_3_	*p*-NO_2_C_6_H_4_	*p*-BrC_6_H_4_	82
9	**6i**	cyclohexyl	CH_2_CH_3_	C_6_H_5_	Bn	77
10	**6j**	*t*-Bu	CH_3_	C_6_H_5_	Bn	70
11	**6k**	Bn	CH_3_	C_6_H_5_	Bn	58

^a^Reaction conditions: cyclohexyl isocyanide (0.1 mmol), dialkyl but-2-ynedioate (0.5 mmol), 1,4-dihydropyridine (0.1 mmol), acetonitrile (5.0 mL), reflux, 1 h; ^b^Isolated yields.

**Scheme 1:**
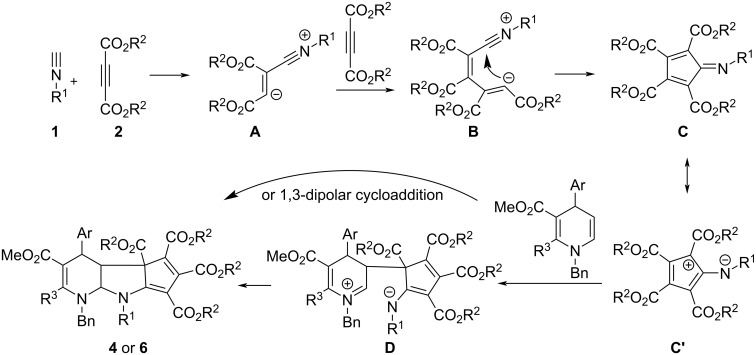
Proposed reaction mechanism.

A plausible reaction mechanism is proposed in Scheme 1 to explain the formation of the polycyclic compounds. At first, the nucleophilic addition of alkyl isocyanide to dialkyl but-2-ynedioate afforded the expected Huisgen’s 1,4-dipolar intermediate **A**. Secondly, the sequential addition of the Huisgen’s 1,4-dipole **A** to the second molecular dialkyl but-2-ynedioate resulted in a 1,5-dipolar intermediate **B**. Thirdly, the intramolecular coupling of the positive charge and the negative charge in intermediate **B** resulted in the formation of polysubstituted 5-(alkylimino)cyclopenta-1,3-diene intermediate **C**, which has been described in several papers about the reaction of alkyl isocyanides and electron-deficient alkynes [59–63]. The in situ generated cyclic intermediate **C** has a resonance hybrid **C’**. Then, the further nucleophilic addition of the electron-rich enamino unit to 5-(alkylimino)cyclopenta-1,3-diene intermediate **C** gave intermediate **D**. At last, the coupling of the iminium cation with the amide anion in intermediate **D** afforded the final product **4** or **6**. On the other hand, the final product **4** or **6** might be directly produced by dipolar cycloaddition reaction of 5-(alkylimino)cyclopenta-1,3-diene intermediate **C** with 5,6-unsaturated dihydropyridine. In consideration of the high diastereoselectivity of the reaction, the concerted addition process is much more likely. However, it is difficult to distinguish between these two reaction processes at present.

## Conclusion

In summary, we investigated the three-component reaction of alkyl isocyanides, dialkyl but-2-ynedioates and 5,6-unsubstituted 1,4-dihydropyridines in refluxing acetonitrile. This reaction provided an efficient synthetic protocol for the polyfunctionalized tetrahydrocyclopenta[4,5]pyrrolo[2,3-*b*]pyridine-3,4b,5,6,7(1*H*)-pentacarboxylates in high yields and with high diastereoselectivity. A novel example of an activated intermediate derived from the reaction of alkyl isocyanide and two molecules of but-2-ynedioate was successfully explored in the reaction. This reaction has the advantages of using readily available reagents, simple reaction conditions, high atomic convergence and atomic economy, which might be found potential applications in heterocyclic chemistry.

## Experimental

### General procedure for the multicomponent reaction

To a round flask was added alkyl isocyanide (0.1 mmol), dialkyl but-2-ynedioate (0.5 mmol), 5,6-unsubstitued 1,4-dihydropyridine (0.1 mmol) and acetonitrile (5.0 mL). The solution was stirred at reflux temperature for nearly one hour. After removing the solvent by rotatory evaporation at reduced pressure, the residue was subjected to column chromatography with a mixture of ethyl acetate and petroleum ether (v/v = 1:4) as eluent to give the pure product for analysis.

**Pentamethyl *rel*-(4*R*,4a*R*,4b*S*,8a*S*)-1-benzyl-8-cyclohexyl-4-(2-methoxyphenyl)-2-methyl-4,4a,8,8a-tetrahydrocyclopenta[4,5]pyrrolo[2,3-*b*]pyridine-3,4b,5,6,7(1*H*)-pentacarboxylate (4a)**: yellow solid, 89%, mp 209–211 °C; ^1^H NMR (400 MHz, CDCl_3_) δ 7.49 (d, *J* = 8.0 Hz, 1H, ArH), 7.47–7.44 (m, 3H, ArH), 7.41–7.38 (m, 1H, ArH), 7.02–6.97 (m, 1H, ArH), 6.68–6.62 (m, 3H, ArH), 5.76 (d, *J* = 3.6 Hz, 1H, CH), 4.96–4.90 (m, 1H, CH), 4.84 (d, *J* = 16.0 Hz, 1H, CH_2_), 4.36 (d, *J* = 15.6 Hz, 1H, CH_2_), 3.95 (s, 3H, OCH_3_), 3.74 (s, 3H, OCH_3_), 3.65–3.63 (m, 1H, CH), 3.61 (s, 3H, OCH_3_), 3.58 (s, 3H, OCH_3_), 3.38 (d, *J* = 8.4 Hz, 1H, CH), 3.21 (s, 3H, OCH_3_), 2.96 (s, 3H, OCH_3_), 2.28 (s, 3H, CH_3_), 1.89–1.86 (m, 2H, CH_2_), 1.80–1.68 (m, 4H, CH_2_), 1.55–1.33 (m, 3H, CH_2_), 1.20–1.13 (m, 1H, CH_2_) ppm; ^13^C NMR (100 MHz, CDCl_3_) δ 170.1, 168.3, 167.8, 166.5, 162.5, 161.9, 157.2, 157.0, 144.7, 137.6, 131.4, 130.9, 129.1, 128.0, 127.9, 127.1, 112.0, 113.6, 111.0, 110.2, 98.9, 84.0, 72.2, 58.2, 56.7, 55.2, 53.3, 52.2, 51.3, 50.4, 50.2, 32.4, 31.5, 31.2, 26.2, 25.7, 25.6, 18.2 ppm; IR (KBr) ν: 3435, 2931, 2862, 2360, 1737, 1698, 1587, 1547, 1435, 1385, 1335, 1251, 1204, 1125, 1092, 1001, 977, 895, 853, 792 cm^−1^; HRESIMS (*m*/*z*): [M + Na]^+^ calcd. for C_41_H_46_NaN_2_O_11_, 765.2994; found, 765.2993.

## Supporting Information

The crystallographic data of the compounds **4a** (CCDC 2346135) and **6g** (CCDC 2346136) have been deposited at the Cambridge Crystallographic Database Center (http://www.ccdc.cam.ac.uk).

File 1 Characterization data and ^1^H NMR, ^13^C NMR, and HRMS spectra of compounds.

## Data Availability

All data that supports the findings of this study is available in the published article and/or the supporting information to this article.
