# Supplementary material for: Rapid construction of tricyclic tetrahydrocyclopenta[4,5]pyrrolo[2,3-b]pyridine via isocyanide-based multicomponent reaction
Source: Beilstein J Org Chem. 2024 Jun 28;20:1436–43. doi: 10.3762/bjoc.20.126 (PMC11216090; doi:10.3762/bjoc.20.126)
Supplement: File 1 — Characterization data and 1H NMR, 13C NMR, and HRMS spectra of compounds. [file Beilstein_J_Org_Chem-20-1436-s001.pdf]

BEILSTEIN JOURNAL OF ORGANIC CHEMISTRY

Supporting Information

for

Rapid construction of tricyclic tetrahydrocyclopenta[4,5]pyrrolo[2,3-*b*]pyridine via isocyanide-based multicomponent reaction

Xiu-Yu Chen, Ying Han, Jing Sun and Chao-Guo Yan

Beilstein J. Org. Chem. **2024,** *20,* 1436–1443. doi:10.3762/bjoc.20.126

Characterization data and ^{1}H NMR, ^{13}C NMR, and HRMS spectra of compounds

License and Terms: This is a supporting information file under the terms of the Creative Commons Attribution License (https://creativecommons.org/licenses/by/4.0). Please note that the reuse, redistribution and reproduction in particular requires that the author(s) and source are credited and that individual graphics may be subject to special legal provisions.
The license is subject to the *Beilstein Journal of Organic Chemistry* terms and conditions: (https://www.beilstein-journals.org/bjoc/terms)

Experiments and general procedure **S2**

Characterization data, ^{1}H NMR, ^{13}C NMR and HRMS of the compounds **S3–S64**

Experimental section

1. General procedure for the multicomponent reaction:

To a round flask was added alkyl isocyanide (0.1 mmol), dialkyl but-2-ynedioate (0.5 mmol), 5,6-unsubstitued 1,4-dihydropyridine (0.1 mmol) and acetonitrile (5.0 mL). The solution was stirred at reflux temperature for nearly one hour. After removing the solvent by rotatory evaporation at reduced pressure, the residue was subjected to column chromatography with a mixture of ethyl acetate and petroleum ether (v/v = 1:4) as eluent to give the pure product for analysis.

Pentamethyl *rel*-(4*R*,4a*R*,4b*S*,8a*S*)-1-benzyl-8-cyclohexyl-4-(2-methoxyphenyl)-2-methyl-4,4a,8,8a-tetrahydrocyclopenta[4,5]pyrrolo[2,3-*b*]pyridine-3,4b,5,6,7(1*H*)-pentacarboxylate (4a): yellow solid, 89%, m.p. 209-211 °C; ^{1}H NMR (400 MHz, $CDCl_3$) δ 7.49 (d, *J* = 8.0 Hz, 1H, ArH), 7.47-7.44 (m, 3H, ArH), 7.41-7.38 (m, 1H, ArH),7.02-6.97 (m, 1H, ArH), 6.68-6.62 (m, 3H, ArH), 5.76 (d, *J* = 3.6 Hz, 1H, CH), 4.96-4.90 (m, 1H, CH),4.84 (d, *J* = 16.0 Hz, 1H, CH_2), 4.36 (d, *J* = 15.6 Hz, 1H, CH_2), 3.95 (s, 3H, OCH_3), 3.74 (s, 3H, OCH_3), 3.65-3.63 (m, 1H, CH), 3.61 (s, 3H, OCH_3), 3.58 (s, 3H, OCH_3), 3.38 (d, *J* = 8.4 Hz, 1H, CH), 3.21 (s, 3H, OCH_3), 2.96 (s, 3H, OCH_3), 2.28 (s, 3H, CH_3), 1.89-1.86 (m, 2H, CH_2), 1.80-1.68 (m, 4H, CH_2), 1.55-1.33 (m, 3H, CH_2), 1.20-1.13 (m, 1H, CH_2) ppm. ^{13}C NMR (100 MHz, $CDCl_3$) δ 170.1, 168.3, 167.8, 166.5, 162.5, 161.9, 157.2, 157.0, 144.7, 137.6, 131.4, 130.9, 129.1, 128.0, 127.9, 127.1, 112.0, 113.6, 111.0, 110.2, 98.9, 84.0, 72.2, 58.2, 56.7, 55.2, 53.3, 52.2, 51.3, 50.4, 50.2, 32.4, 31.5, 31.2, 26.2, 25.7, 25.6, 18.2 ppm. IR (KBr) υ: 3435, 2931, 2862, 2360, 1737, 1698, 1587, 1547, 1435, 1385, 1335, 1251, 1204, 1125, 1092, 1001, 977, 895, 853, 792 cm^{-1}; MS (*m*/*z*): HRMS (ESI) Calcd. for $C_{41}H_{46}NaN_2O_{11}$ ([M+Na]$^{+}$): 765.2994, found: 765.2993.

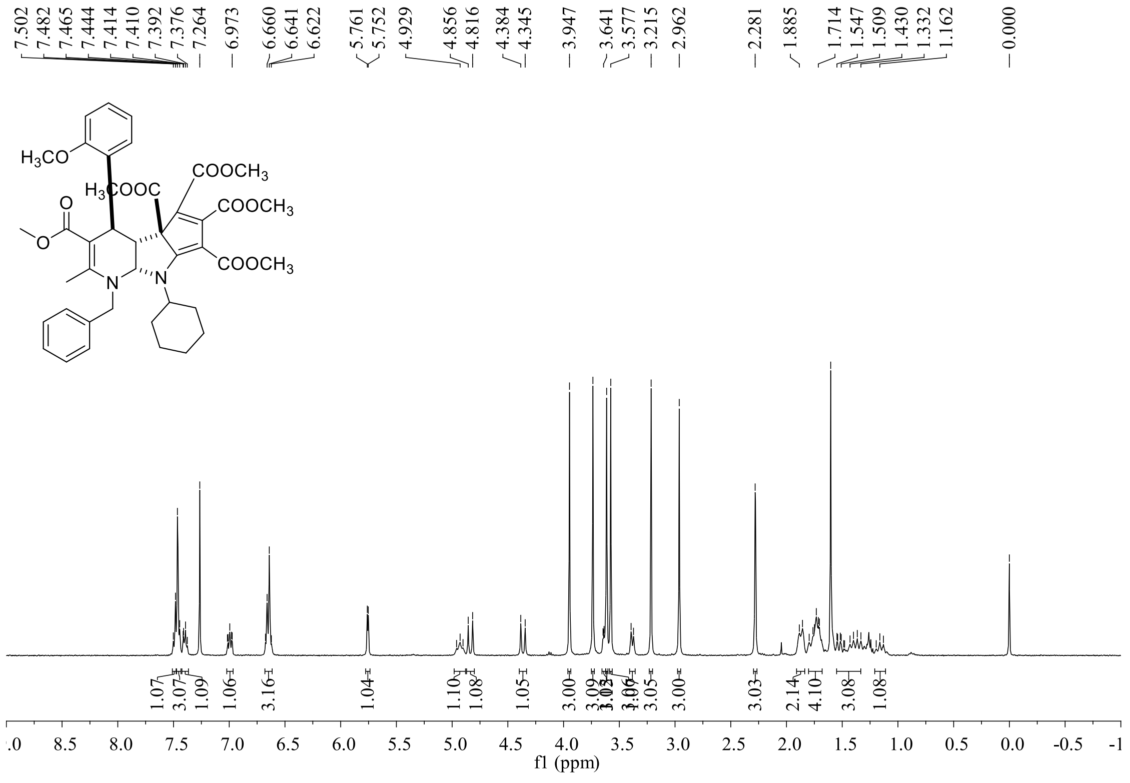

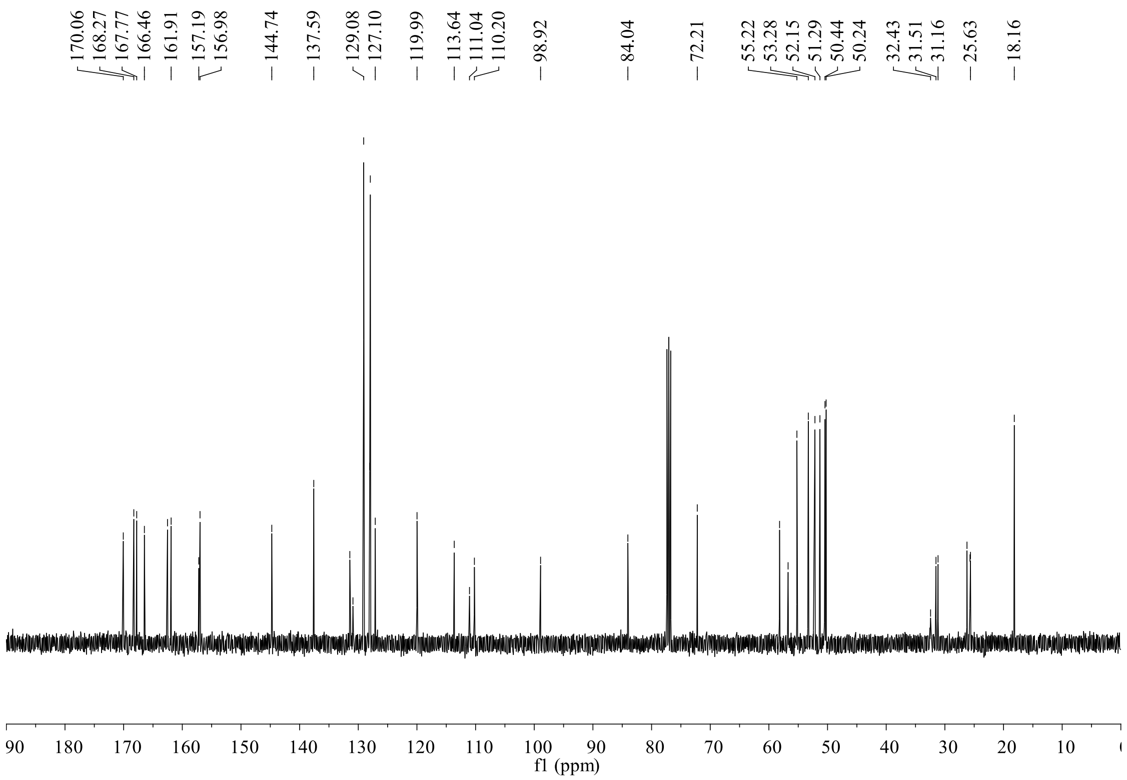
170.06
168.27
167.77
166.46
161.91
157.19
156.98
144.74
137.59
129.08
127.10
119.99
113.64
111.04
110.20
98.92
84.04
72.21
55.22
53.28
52.15
51.29
50.44
50.24
32.43
31.51
31.16
25.63
18.16
90 180 170 160 150 140 130 120 110 100 90 80 70 60 50 40 30 20 10
f1 (ppm)

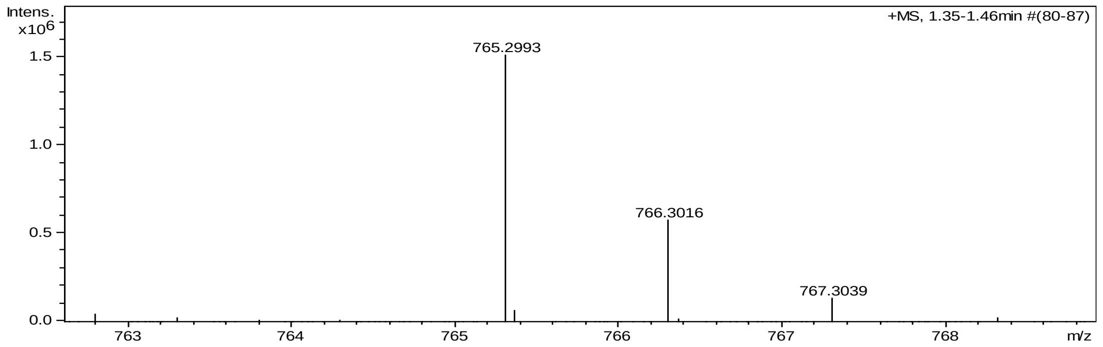
Intens.
x10^6
+MS, 1.35-1.46min #(80-87)
1.5
1.0
0.5
0.0
765.2993
766.3016
767.3039
763
764
765
766
767
768
m/z

Pentamethyl *rel*-(4*R*,4a*R*,4b*S*,8a*S*)-1-benzyl-8-cyclohexyl-2-methyl-4-(4-nitrophenyl)-4,4a,8,8a-tetrahydrocyclopenta[4,5]pyrrolo[2,3-*b*]pyridine-3,4b,5,6,7(1*H*)-pentacarboxylate (4b): yellow solid, 84%, m.p. 211-213 °C; ^{1}H NMR (400 MHz, $CDCl_3$) δ 7.89 (d, *J* = 8.8 Hz, 2H, ArH), 7.54-7.47 (m, 3H, ArH), 7.43-7.41 (m, 2H, ArH), 6.79 (d, *J* = 8.8 Hz, 2H, ArH), 5.87 (d, *J* = 3.6 Hz, 1H, CH), 4.93 (d, *J* = 15.6 Hz, 1H, CH_2), 4.90-4.86 (m, 1H, CH),4.31 (d, *J* = 15.2 Hz, 1H, CH_2), 3.96 (s, 3H, OCH_3), 3.74 (s, 3H, OCH_3), 3.60 (s, 3H, OCH_3), 3.30 (s, 3H, OCH_3), 3.19 (dd, J_1 = 8.4 Hz, J_2 = 3.6 Hz, 1H, CH), 3.07-3.06 (m, 1H, CH), 3.06 (s, 3H, OCH_3), 2.42 (s, 3H, CH_3), 1.94-1.85 (m, 2H, CH_2), 1.79-1.73 (m, 4H, CH_2), 1.48-1.35 (m, 3H, CH_2), 1.21-1.11 (m, 1H, CH_2) ppm. ^{13}C NMR (100 MHz, $CDCl_3$) δ 169.9, 167.3, 167.1, 166.0, 162.2, 161.7, 158.3, 152.7, 148.1, 146.0, 136.9, 129.7, 129.3, 128.6, 128.2, 122.8, 112.3, 110.6, 110.0, 99.7, 83.3, 72.1, 58.6, 56.7, 53.5, 52.5, 52.1, 51.5, 50.8, 50.6, 38.2, 32.0, 31.1, 26.1, 25.62, 25.56, 18.8 ppm. IR (KBr) υ: 3435, 2949, 2857, 1736, 1698, 1583, 1542, 1434, 1383, 1348, 1233, 1208, 1117, 1091, 1001, 980, 894, 847, 795 cm^{-1}; MS (*m*/*z*): HRMS (ESI) Calcd. for $C_{40}H_{43}NaN_3O_{12}$ ([M+Na]$^{+}$): 780.2739, found: 780.2733.

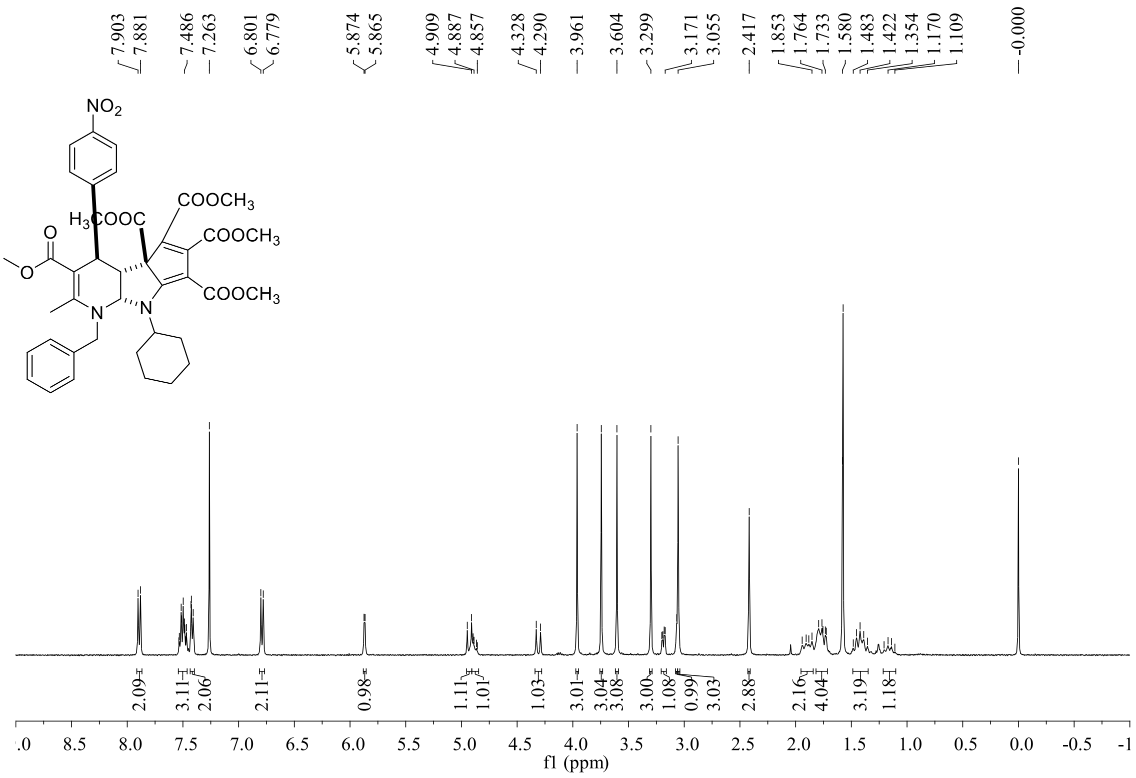

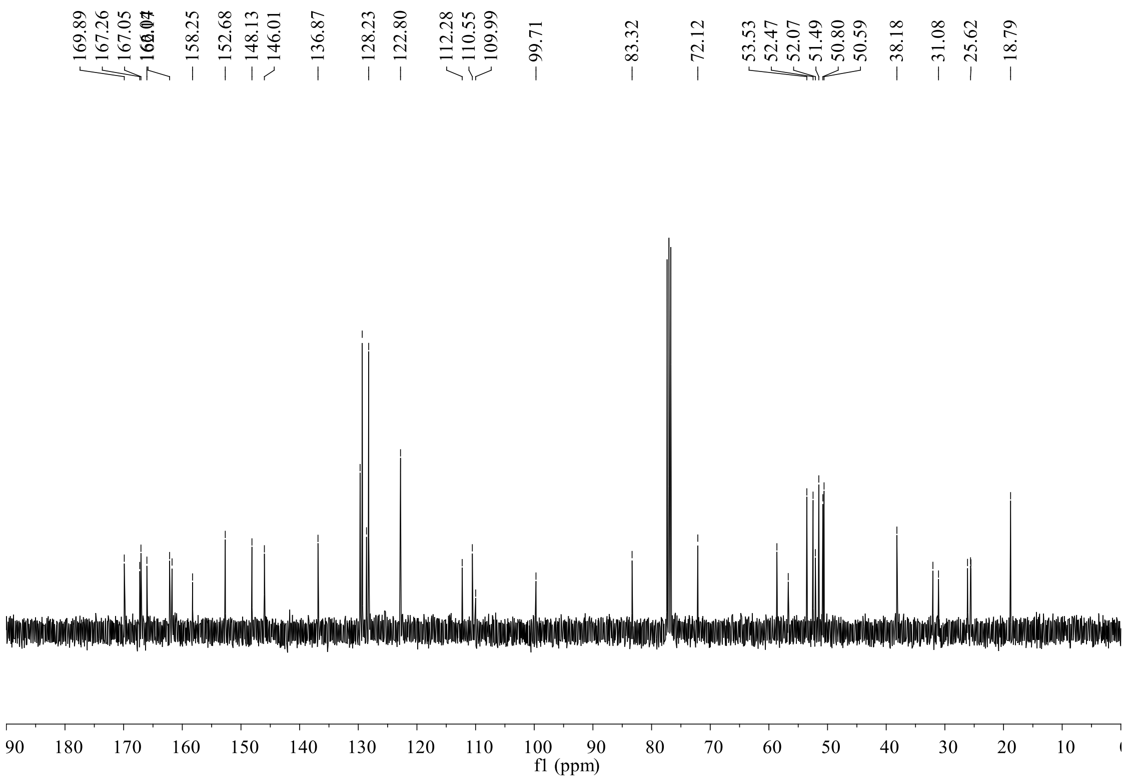
169.89
167.26
167.05
158.25
152.68
148.13
146.01
136.87
128.23
122.80
112.28
110.55
109.99
99.71
83.32
72.12
53.53
52.47
52.07
51.49
50.80
50.59
38.18
31.08
25.62
18.79
f1 (ppm)

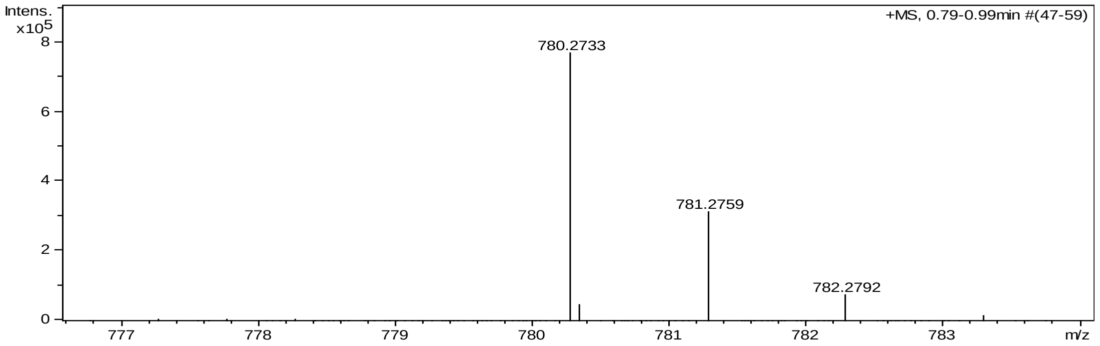
Intens.
x10^5
+MS, 0.79-0.99min #(47-59)
780.2733
781.2759
782.2792
m/z

Pentamethyl *rel*-(4*R*,4a*R*,4b*S*,8a*S*)-1-benzyl-8-cyclohexyl-2-methyl-4-(*p*-tolyl)-4,4a,8,8a-tetrahydrocyclopenta[4,5]pyrrolo[2,3-*b*]pyridine-3,4b,5,6,7(1*H*)-pentacarboxylate (4c): yellow solid, 81%, m.p. 185-187 °C; ^{1}H NMR (400 MHz, $CDCl_3$) δ 7.50-7.47 (m, 2H, ArH), 7.42-7.40 (m, 3H, ArH), 6.86 (d, *J* = 7.6 Hz, 2H, ArH), 6.64 (d, *J* = 8.0 Hz, 2H, ArH), 5.78 (d, *J* = 3.6 Hz, 1H, CH), 4.94-4.90 (m, 1H, CH), 4.87 (d, *J* = 16.0 Hz, 1H, CH_2), 4.28 (d, *J* = 15.2 Hz, 1H, CH_2), 3.95 (s, 3H, OCH_3), 3.73 (s, 3H, OCH_3), 3.57 (s, 3H, OCH_3), 3.28 (s, 3H, OCH_3), 3.26-3.25 (m, 1H, CH), 2.97 (s, 3H, OCH_3), 2.96-2.92 (m, 1H, CH), 2.31 (s, 3H, CH_3), 2.18 (s, 3H, CH_3), 1.92-1.85 (m, 2H, CH_2), 1.78-1.72 (m, 4H, CH_2), 1.48-1.35 (m, 3H, CH_2), 1.20-1.13 (m, 1H, CH_2) ppm. ^{13}C NMR (100 MHz, $CDCl_3$) δ 170.2, 167.9, 167.6, 166.4, 162.4, 162.0, 157.5, 145.1, 140.6, 137.2, 135.1, 129.1, 128.8, 128.3, 128.2, 128.0, 113.0, 112.1, 99.1, 83.8, 72.1, 58.3, 56.6, 53.3, 52.4, 52.3, 51.4, 50.6, 50.5, 37.7, 31.8, 31.1, 26.2, 25.7, 25.6, 20.8, 18.4 ppm. IR (KBr) υ: 3435, 2949, 2859, 1737, 1698, 1581, 1541, 1435, 1387, 1338, 1232, 1205, 1124, 1092, 1001, 978, 894, 852, 788 cm^{-1}; MS (*m/z*): HRMS (ESI) Calcd. for $C_{41}H_{46}NaN_2O_{10}$ ([M+Na]$^+$): 749.3045, found: 749.3039.

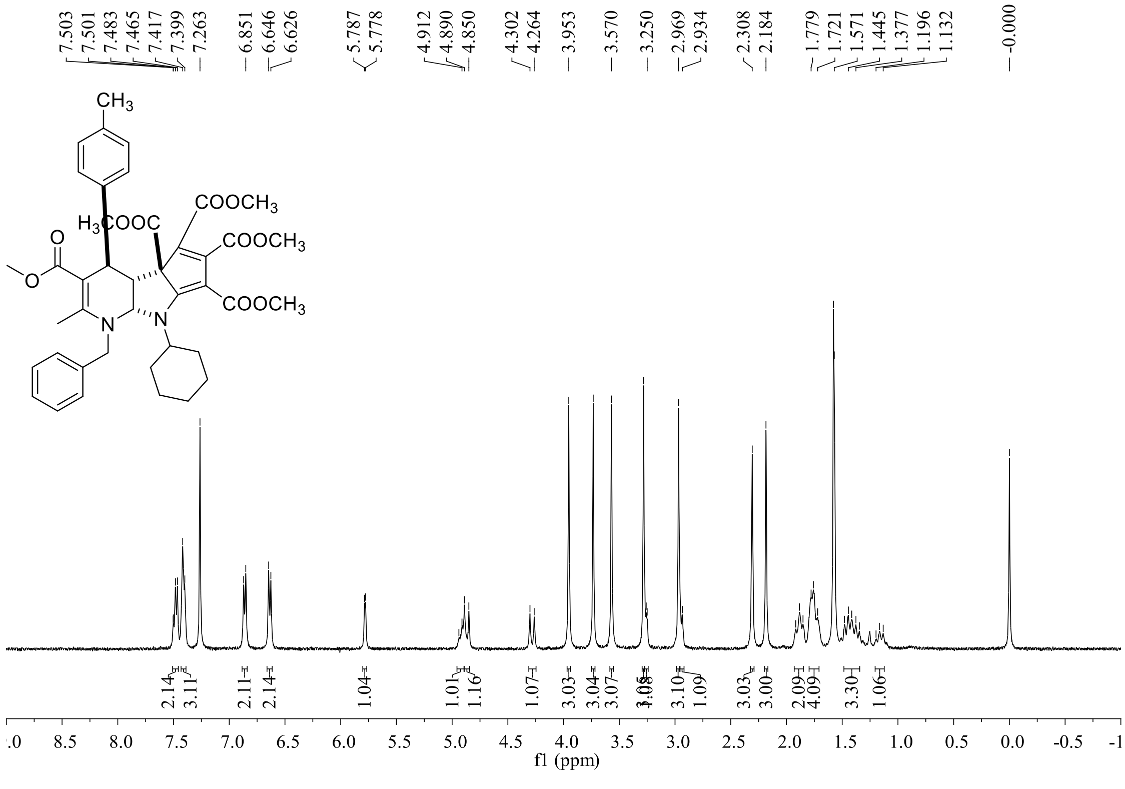

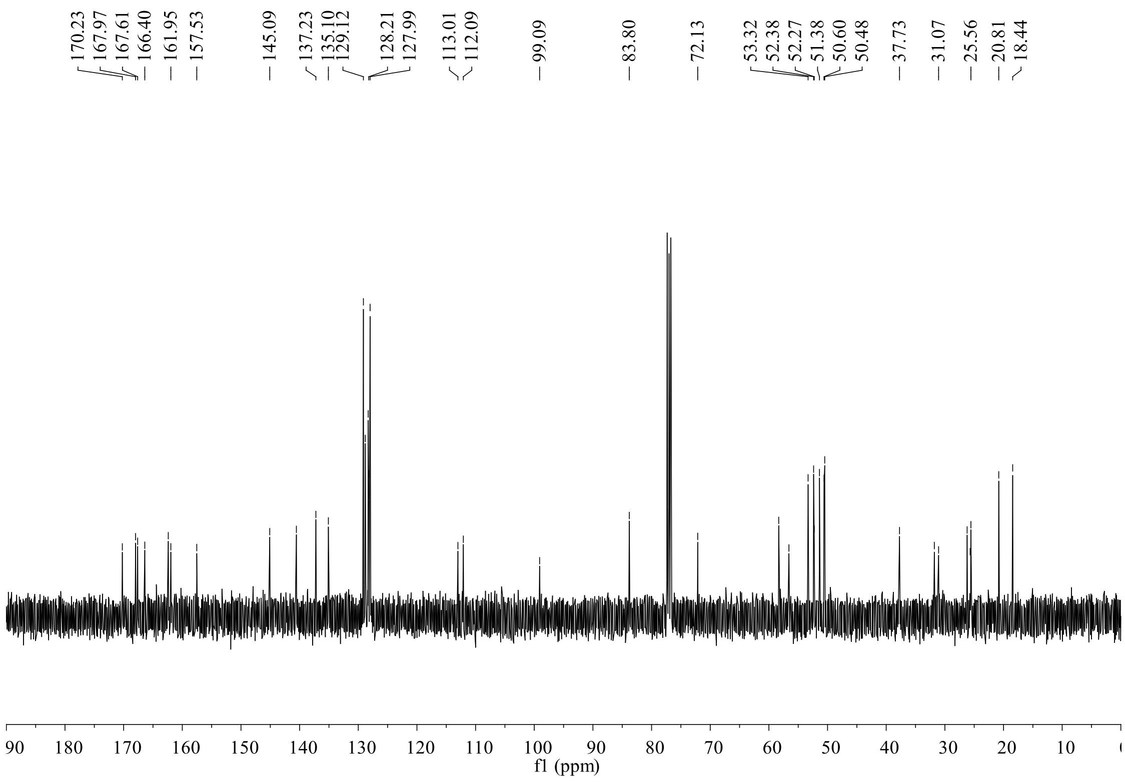
170.23
167.97
167.61
166.40
161.95
157.53
145.09
137.23
135.10
129.12
128.21
127.99
113.01
112.09
99.09
83.80
72.13
53.32
52.38
52.27
51.38
50.60
50.48
37.73
31.07
25.56
20.81
18.44
f1 (ppm)

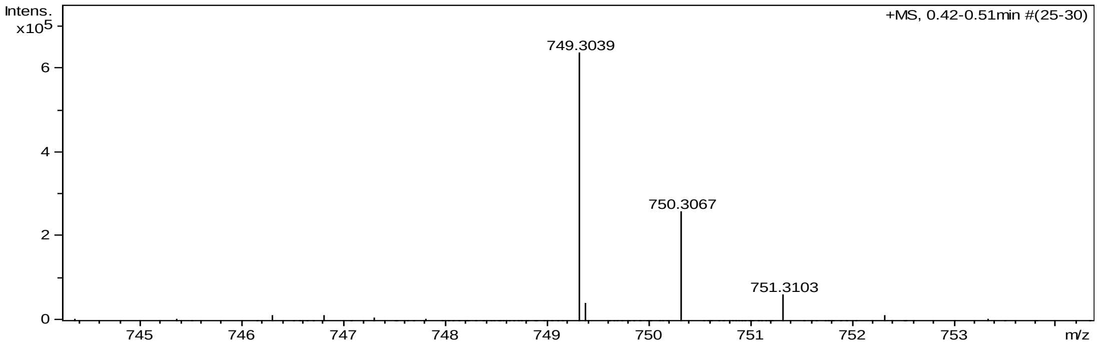
Intens.
x10^5
+MS, 0.42-0.51min #(25-30)
749.3039
750.3067
751.3103
m/z

Pentamethyl *rel*-(4*R*,4a*R*,4b*S*,8a*S*)-8-cyclohexyl-1-(4-methoxybenzyl)-4-(2-methoxyphenyl)-2-methyl-4,4a,8,8a-tetrahydrocyclopenta[4,5]pyrrolo[2,3-*b*]pyridine-3,4b,5,6,7(1*H*)-pentacarboxylate (4d): yellow solid, 84%, m.p. 183-185 °C; ^{1}H NMR (400 MHz, $CDCl_3$) δ 7.37 (d, *J* = 8.8 Hz, 2H, ArH), 7.01-6.97 (m, 3H, ArH), 6.65-6.62 (m, 2H, ArH), 6.55 (d, *J* = 6.8 Hz, 1H, ArH), 5.75 (d, *J* = 3.6 Hz, 1H, CH), 4.93-4.87 (m, 1H, CH),4.77 (d, *J* = 15.6 Hz, 1H, CH_2), 4.30 (d, *J* = 15.2 Hz, 1H, CH_2), 3.95 (s, 3H, OCH_3), 3.86 (s, 3H, OCH_3), 3.76 (s, 3H, OCH_3), 3.61 (s, 3H, OCH_3), 3.59 (s, 3H, OCH_3), 3.58-3.55 (m, 1H, CH), 3.39 (d, *J* = 8.4 Hz, 1H, CH), 3.22 (s, 3H, OCH_3), 2.97 (s, 3H, OCH_3), 2.29 (s, 3H, CH_3), 1.87-1.84 (m, 2H, CH_2), 1.79-1.68 (m, 4H, CH_2), 1.54-1.32 (m, 3H, CH_2), 1.19-1.12 (m, 1H, CH_2) ppm. ^{13}C NMR (100 MHz, $CDCl_3$) δ 170.0, 168.3, 167.9, 166.5, 162.5, 161.9, 159.4, 157.2, 156.9, 145.1, 131.7, 130. 7, 129.7, 129.4, 127.1, 119.9, 114.4, 113.6, 111.4, 110.1, 98.9, 84.0, 72.3, 58.2, 56.1, 55.4, 55.2, 53.3, 52.2, 51.3, 50.7, 50.4, 50.3, 31.6, 31.1, 26.2, 25.7, 25.6, 18.3 ppm. IR (KBr) υ: 3435, 2947, 2857, 1733, 1697, 1583, 1549, 1436, 1384, 1334, 1251, 1201, 1108, 1087, 1032, 1000, 894, 819, 791, 756 cm^{-1}; MS (*m*/*z*): HRMS (ESI) Calcd. for $C_{42}H_{48}NaN_2O_{12}$ ($[M+Na]^+$): 795.3099, found: 795.3099.

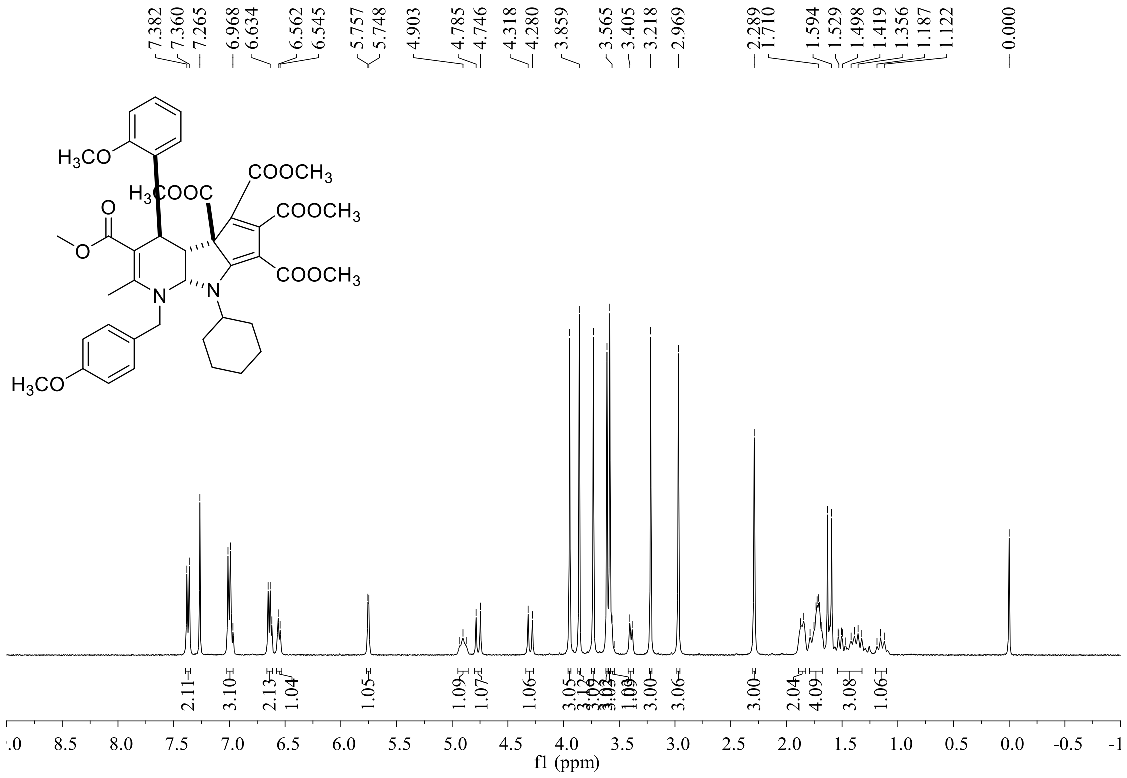

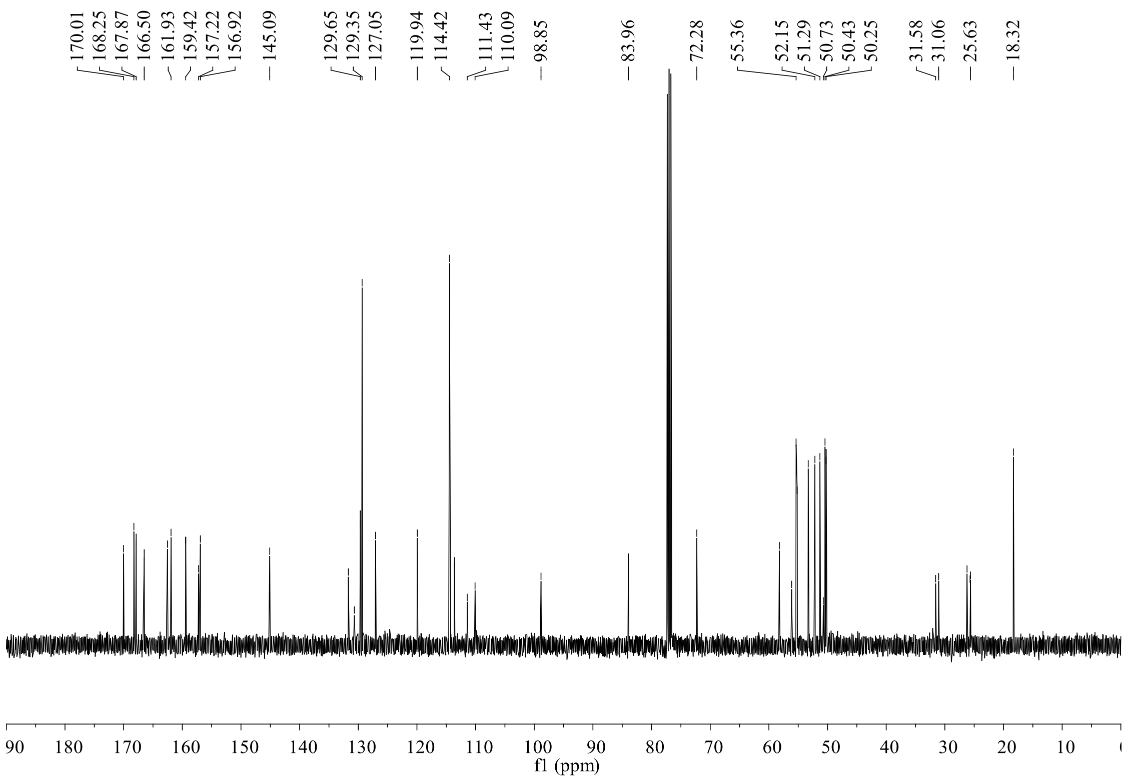
170.01
168.25
167.87
166.50
161.93
159.42
157.22
156.92
145.09
129.65
129.35
127.05
119.94
114.42
111.43
110.09
98.85
83.96
72.28
55.36
52.15
51.29
50.73
50.43
50.25
31.58
31.06
25.63
18.32
f1 (ppm)

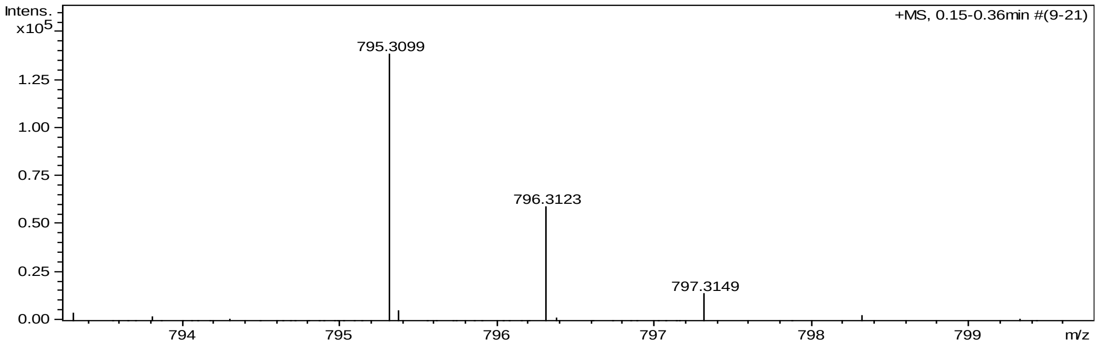
Intens.
x10^5
+MS, 0.15-0.36min #(9-21)
795.3099
796.3123
797.3149
m/z

Pentamethyl *rel*-(4*R*,4a*R*,4b*S*,8a*S*)-1-(4-chlorobenzyl)-8-cyclohexyl-2-methyl-4-phenyl-4,4a,8,8a-tetrahydrocyclopenta[4,5]pyrrolo[2,3-*b*]pyridine-3,4b,5,6,7(1*H*)-pentacarboxylate (4e): yellow solid, 82%, m.p. 206-208 °C; ^{1}H NMR (400 MHz, $CDCl_3$) δ 7.47 (d, *J* = 8.4 Hz, 2H, ArH), 7.36 (d, *J* = 8.4 Hz, 2H, ArH), 7.10-7.06 (m, 2H, ArH), 7.03-7.00 (m, 1H, ArH), 6.70 (d, *J* = 6.8 Hz, 2H, ArH), 5.80 (d, *J* = 3.6 Hz, 1H, CH), 4.93-4.89 (m, 1H, CH), 4.84 (d, *J* = 15.6 Hz, 1H, CH_2), 4.27 (d, *J* = 16.0 Hz, 1H, CH_2), 3.96 (s, 3H, OCH_3), 3.74 (s, 3H, OCH_3), 3.58 (s, 3H, OCH_3), 3.30-3.28 (m, 1H, CH), 3.27 (s, 3H, OCH_3), 3.01-2.99 (m, 1H, CH), 2.99 (s, 3H, OCH_3), 2.30 (s, 3H, CH_3), 1.92-1.86 (m, 2H, CH_2), 1.79-1.71 (m, 4H, CH_2), 1.45-1.35 (m, 3H, CH_2), 1.16-1.12 (m, 1H, CH_2) ppm. ^{13}C NMR (100 MHz, $CDCl_3$) δ 170.0, 167.73, 167.71, 166.3, 162.4, 161.9, 157.6, 145.0, 143.6, 135.7, 134.2, 129.4, 128.8, 127.7, 126.0, 113.1, 112.5, 99.3, 83.7, 77.3, 77.0, 76.7, 72.1, 58.4, 55.9, 53.4, 52.4, 52.3, 51.5, 50.8, 50.5, 38.1, 31.9, 31.1, 26.2, 25.7, 25.5, 18.5 ppm. IR (KBr) υ: 3434, 2950, 2856, 1734, 1692, 1581, 1547, 1435, 1389, 1355, 1228, 1202, 1114, 1090, 1011, 975, 892, 789, 755 cm^{-1}; MS (*m*/*z*): HRMS (ESI) Calcd. for $C_{40}H_{43}ClNaN_2O_{10}$ ([M+Na]$^+$): 769.2498, found: 769.2493.

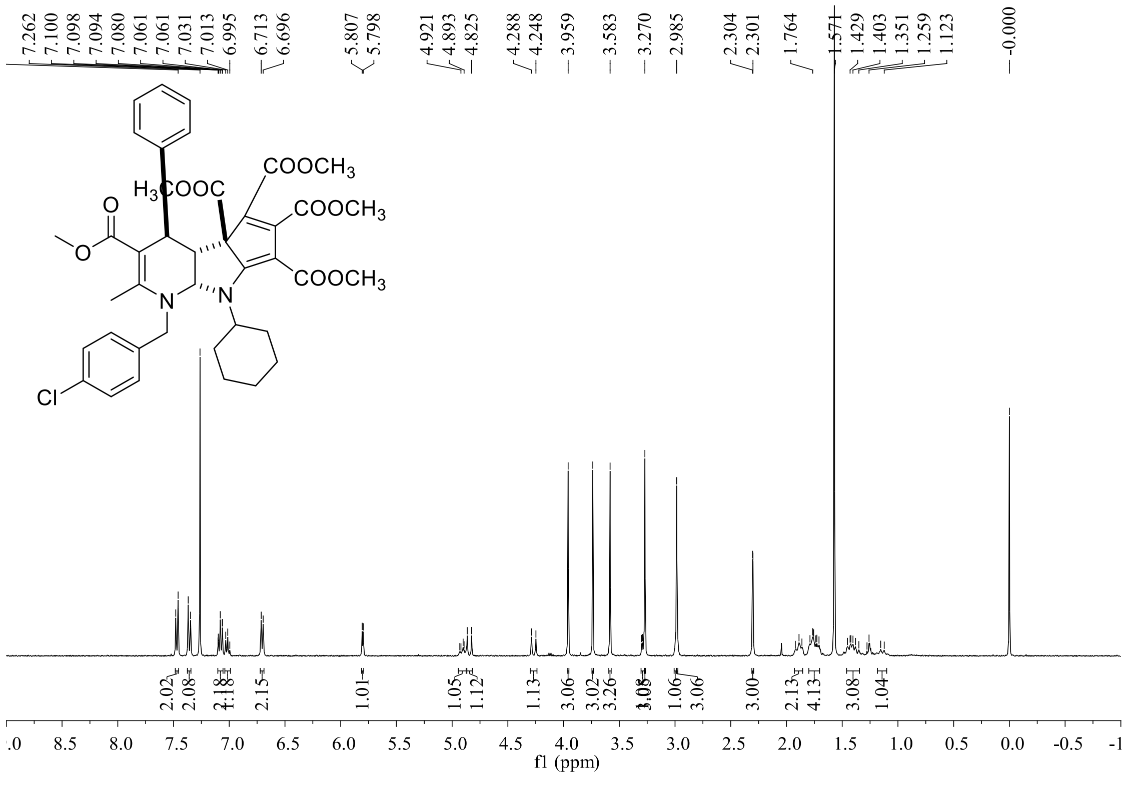

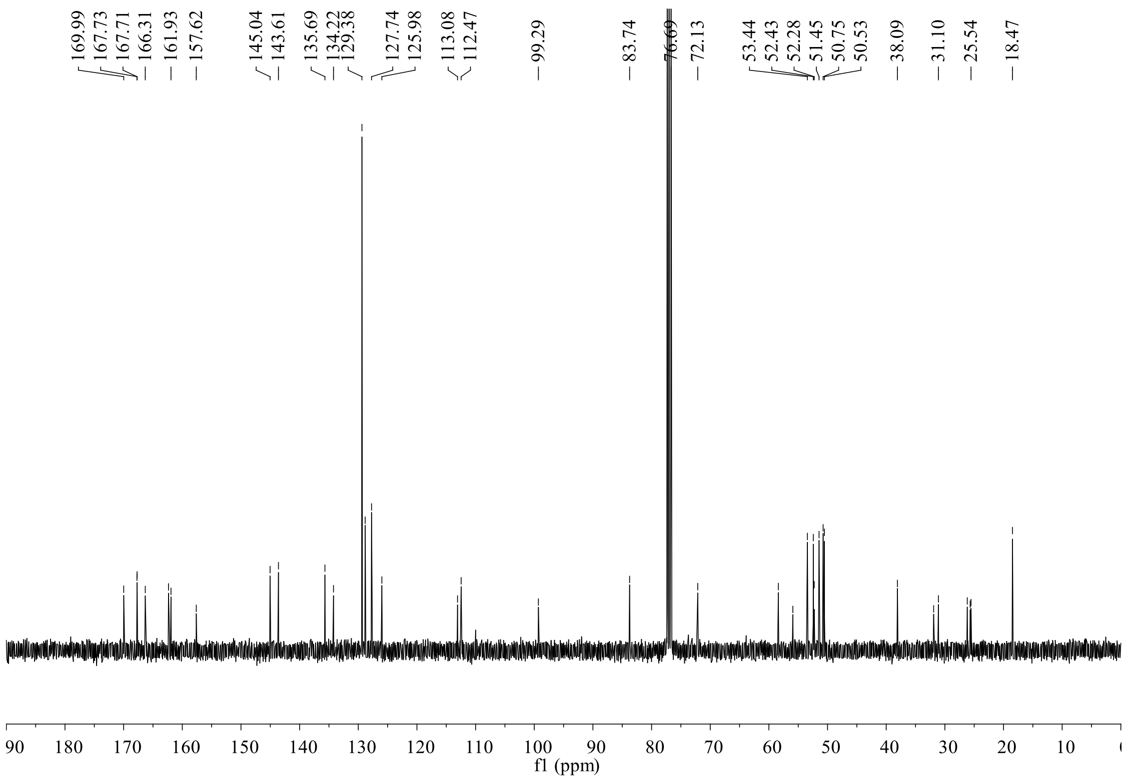
169.99
167.73
167.71
166.31
161.93
157.62
145.04
143.61
135.69
134.22
129.38
127.74
125.98
113.08
112.47
99.29
83.74
76.69
72.13
53.44
52.43
52.28
51.45
50.75
50.53
38.09
31.10
25.54
18.47
90 180 170 160 150 140 130 120 110 100 90 80 70 60 50 40 30 20 10
f1 (ppm)

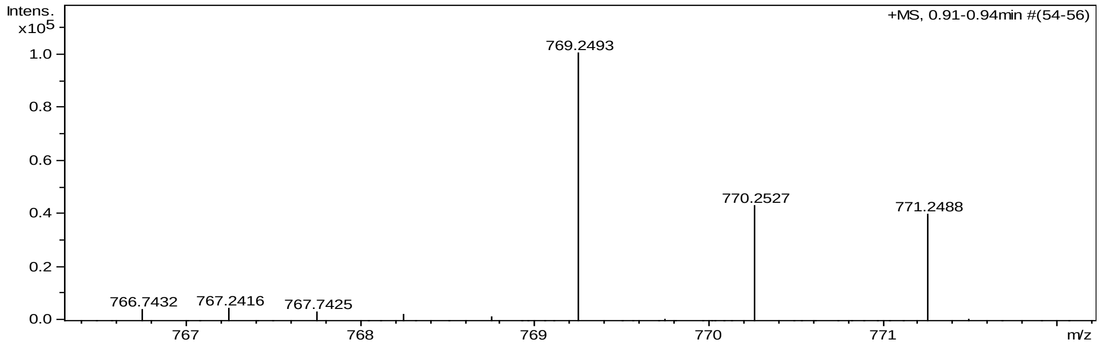
Intens.
x10^5
1.0
0.8
0.6
0.4
0.2
0.0
+MS, 0.91-0.94min #(54-56)
769.2493
770.2527
771.2488
766.7432
767.2416
767.7425
767 768 769 770 771
m/z

Pentamethyl *rel*-(4*R*,4a*R*,4b*S*,8a*S*)-1-benzyl-8-cyclohexyl-2-methyl-4-phenyl-4,4a,8,8a-tetrahydrocyclopenta[4,5]pyrrolo[2,3-*b*]pyridine-3,4b,5,6,7(1*H*)-pentacarboxylate (4f): yellow solid, 90%, m.p. 196-198 °C; ^{1}H NMR (400 MHz, $CDCl_3$) δ 7.51-7.47 (m, 2H, ArH), 7.42-7.40 (m, 3H, ArH), 7.07-7.03 (m, 2H, ArH), 7.01-6.97 (m, 1H, ArH), 6.75 (d, J = 7.2 Hz, 2H, ArH), 5.81 (d, J = 3.6 Hz, 1H, CH), 4.95-4.91 (m, 1H, CH),4.88 (d, J = 15.6 Hz, 1H, CH_2), 4.30 (d, J = 16.0 Hz, 1H, CH_2), 3.96 (s, 3H, OCH_3), 3.74 (s, 3H, OCH_3), 3.58 (s, 3H, OCH_3), 3.30 (dd, J_1 = 8.8 Hz, J_2 = 3.6 Hz, 1H, CH), 3.26 (s, 3H, OCH_3), 2.99-2.97 (m, 1H, CH), 2.96 (s, 3H, OCH_3), 2.33 (s, 3H, CH_3), 1.92-1.86 (m, 2H, CH_2), 1.79-1.72 (m, 4H, CH_2), 1.49-1.35 (m, 3H, CH_2), 1.20-1.10 (m, 1H, CH_2) ppm. ^{13}C NMR (100 MHz, $CDCl_3$) δ 170.2, 167.8, 167.6, 166.4, 162.4, 161.9, 157.7, 145.6, 143.8, 137.2, 129.2, 128.9, 128.3, 128.0, 127.6, 125.8, 113.0, 111.9, 99.2, 83.7, 72.1, 58.4, 56.6, 53.3, 52.4, 52.2, 51.4, 50.7, 50.4, 38.2, 31.8, 31.1, 26.2, 25.7, 25.6, 18.5 ppm. IR (KBr) υ: 3435, 2952, 2862, 1737, 1698, 1583, 1542, 1435, 1386, 1338, 1239, 1204, 1121, 1092, 1000, 978, 894, 852, 789 cm^{-1}; MS (m/z): HRMS (ESI) Calcd. for $C_{40}H_{44}NaN_2O_{10}$ ($[M+Na]^+$): 735.2888, found: 735.2885.

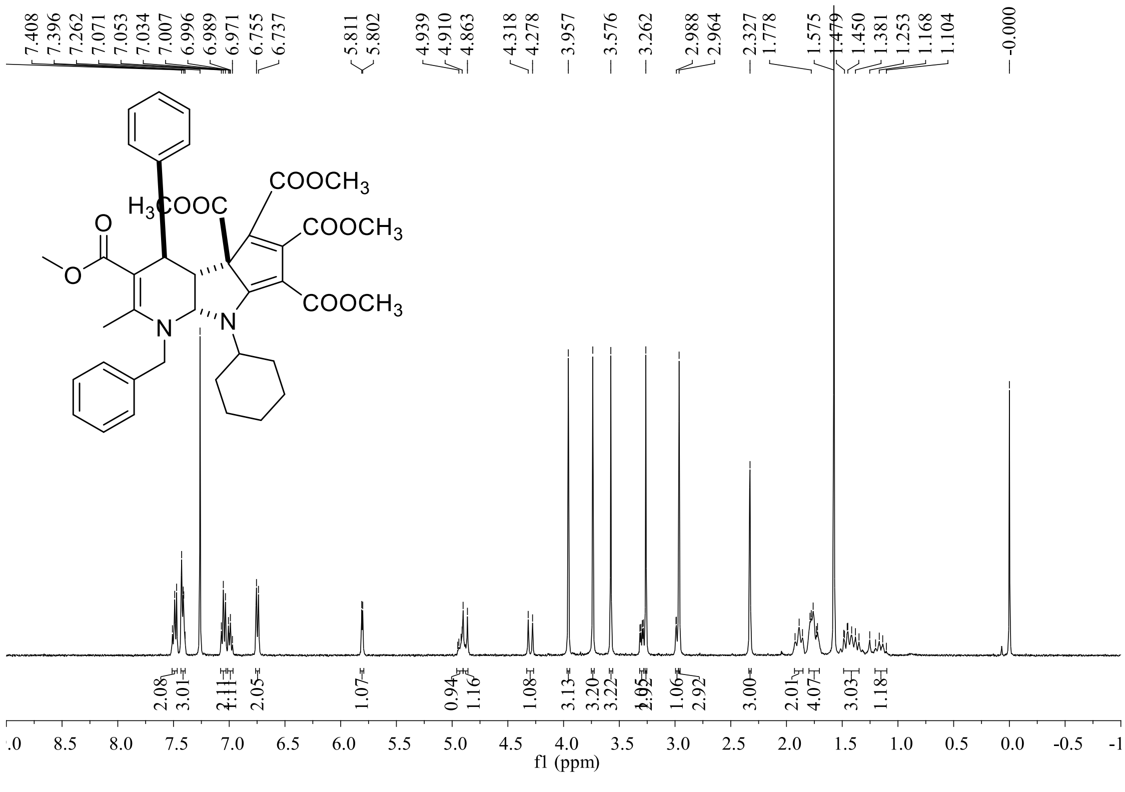

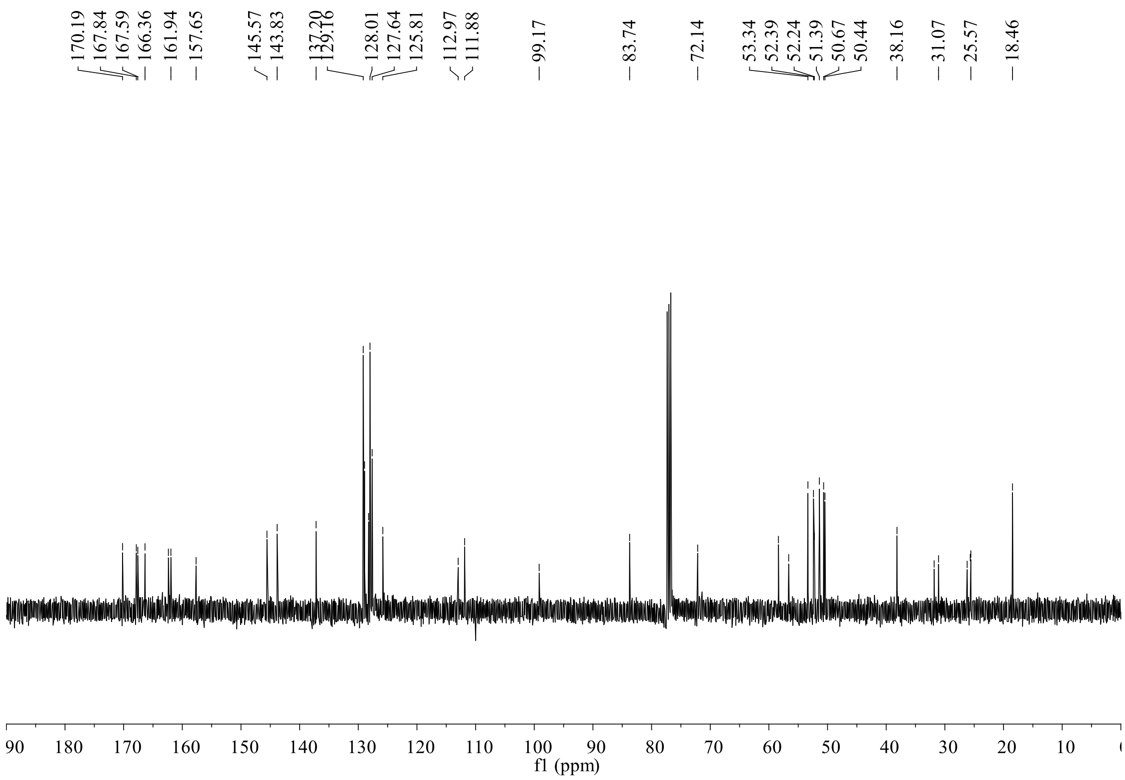
170.19
167.84
167.59
166.36
161.94
157.65
145.57
143.83
137.20
129.16
128.01
127.64
125.81
112.97
111.88
99.17
83.74
72.14
53.34
52.39
52.24
51.39
50.67
50.44
38.16
31.07
25.57
18.46
90 180 170 160 150 140 130 120 110 100 90 80 70 60 50 40 30 20 10
f1 (ppm)

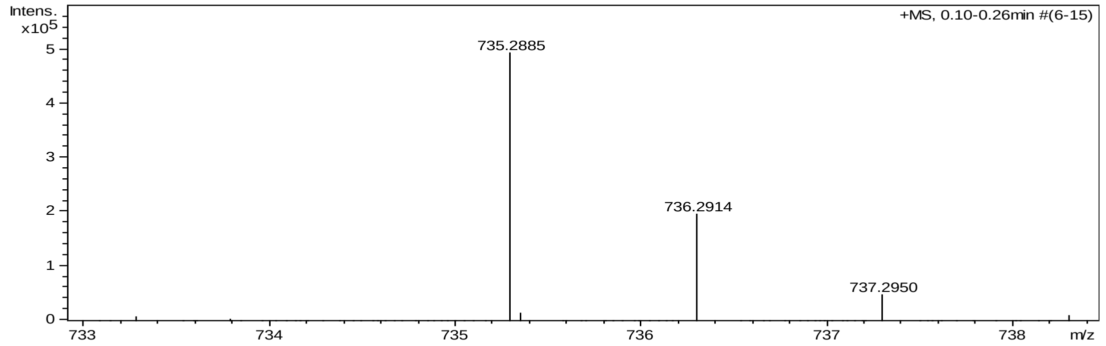
Intens.
x10^5
+MS, 0.10-0.26min #(6-15)
735.2885
736.2914
737.2950
733
734
735
736
737
738
m/z

Pentamethyl *rel*-(4*R*,4a*R*,4b*S*,8a*S*)-8-cyclohexyl-2-methyl-1-(2-methylbenzyl)-4-phenyl-4,4a,8,8a-tetrahydrocyclopenta[4,5]pyrrolo[2,3-*b*]pyridine-3,4b,5,6,7(1*H*)-pentacarboxylate (4g): yellow solid, 81%, m.p. 202-204 °C; ^{1}H NMR (400 MHz, $CDCl_3$) δ 7.43 (d, *J* = 7.2 Hz, 1H, ArH), 7.36 (t, *J* = 6.8 Hz, 1H, ArH), 7.30-7.27 (m, 1H, ArH), 7.25-7.22 (m, 1H, ArH), 7.17-7.13 (m, 2H, ArH), 7.09-7.02 (m, 3H, ArH), 5.64 (d, *J* = 2.8 Hz, 1H, CH), 4.95-4.84 (m, 2H, CH, CH_2),4.38 (d, *J* = 16.4 Hz, 1H, CH_2), 3.96 (s, 3H, OCH_3), 3.74 (s, 3H, OCH_3), 3.52 (s, 3H, OCH_3), 3.50-3.46 (m, 1H, CH), 3.26 (s, 3H, OCH_3), 3.07 (d, *J* = 8.4 Hz, 1H, CH), 2.98 (s, 3H, OCH_3), 2.42 (s, 3H, CH_3), 2.26 (s, 3H, CH_3), 1.94-1.86 (m, 2H, CH_2), 1.83-1.75 (m, 2H, CH_2), 1.74-1.69 (m, 2H, CH_2), 1.60-1.51 (m, 1H, CH_2), 1.42-1.33 (m, 2H, CH_2), 1.18-1.12 (m, 1H, CH_2) ppm. ^{13}C NMR (100 MHz, $CDCl_3$) δ 170.5, 168.1, 167.4, 166.4, 162.4, 161.9, 157.5, 146.1, 143.8, 135.3, 135.1, 131.0, 129.1, 127.9, 127. 8, 127.1, 126.7, 126.0, 113.1, 109.9, 99.4, 83.4, 72.0, 58.3, 53.3, 52.4, 52.3, 51.4, 50.7, 50.4, 38.4, 31.5, 31.4, 26.3, 25.70, 25.66, 19.3, 17.7 ppm. IR (KBr) υ: 3446, 2947, 1733, 1687, 1581, 1545, 1435, 1383, 1339, 1233, 1204, 1131, 1092, 981, 894, 818, 790 cm^{-1}; MS (m/z): HRMS (ESI) Calcd. for $C_{41}H_{46}NaN_2O_{10}$ ([M+Na]+): 749.3045, found: 749.3039.

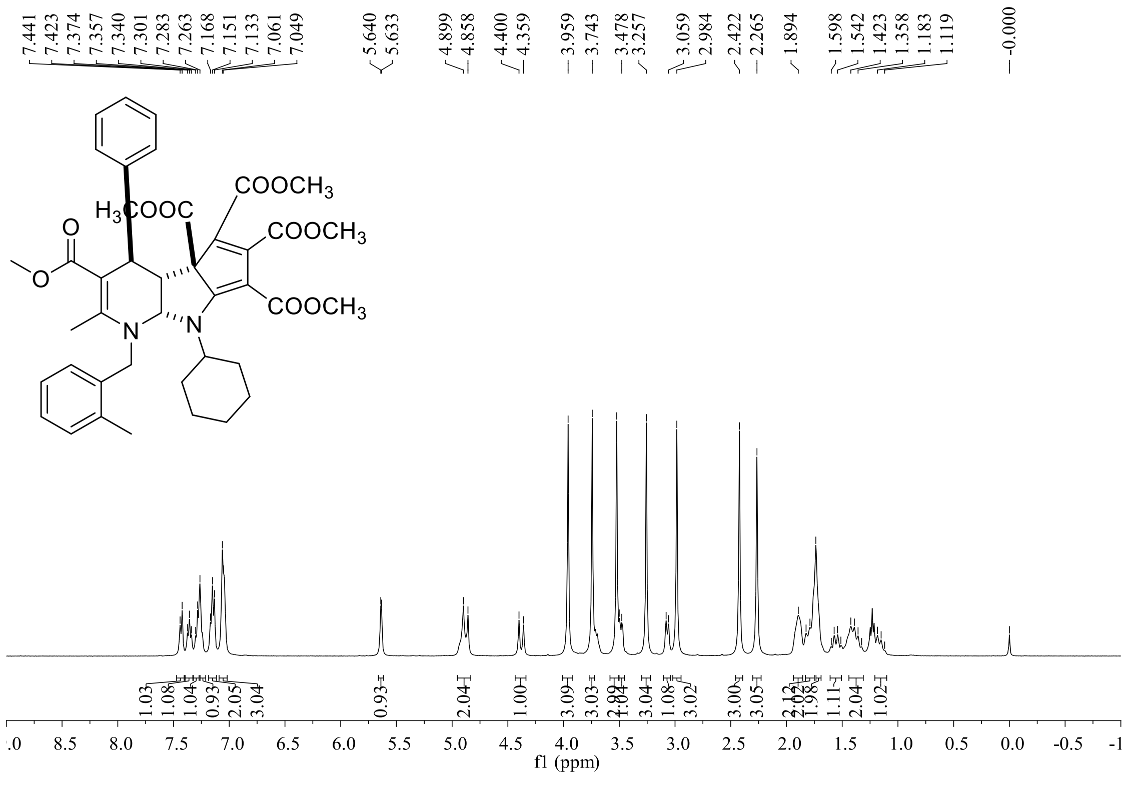

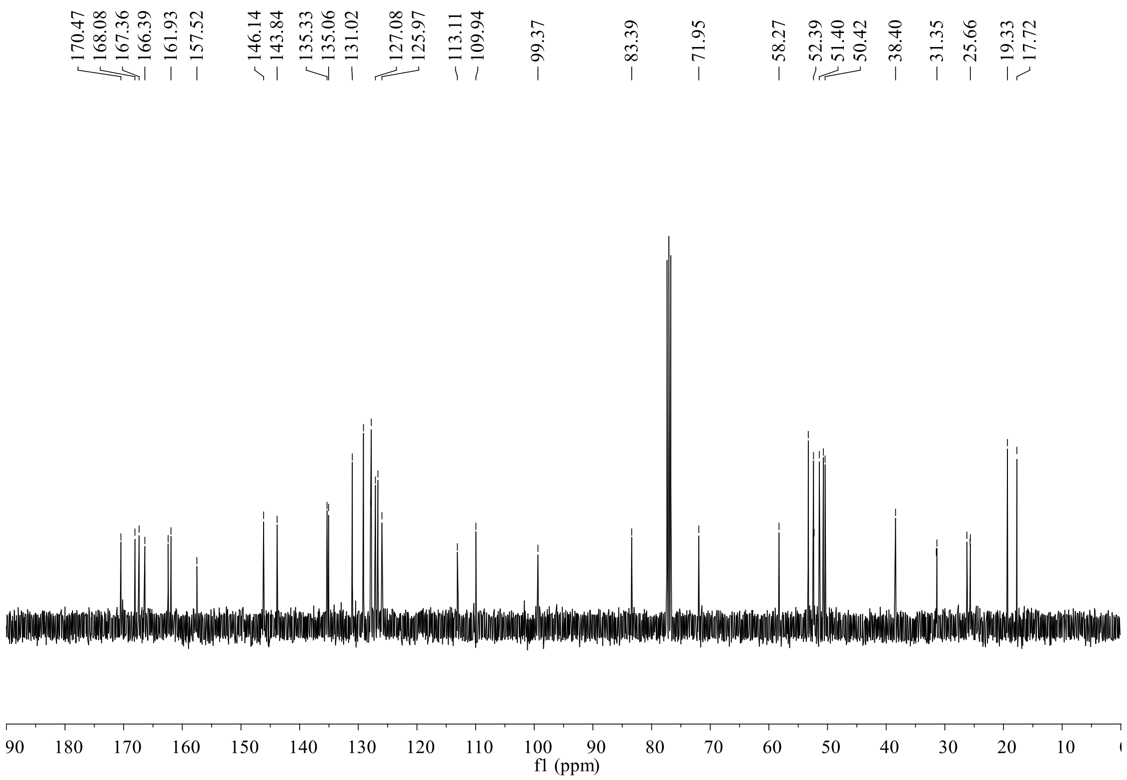
170.47
168.08
167.36
166.39
161.93
157.52
146.14
143.84
135.33
135.06
131.02
127.08
125.97
113.11
109.94
99.37
83.39
71.95
58.27
52.39
51.40
50.42
38.40
31.35
25.66
19.33
17.72
90 180 170 160 150 140 130 120 110 100 90 80 70 60 50 40 30 20 10
f1 (ppm)

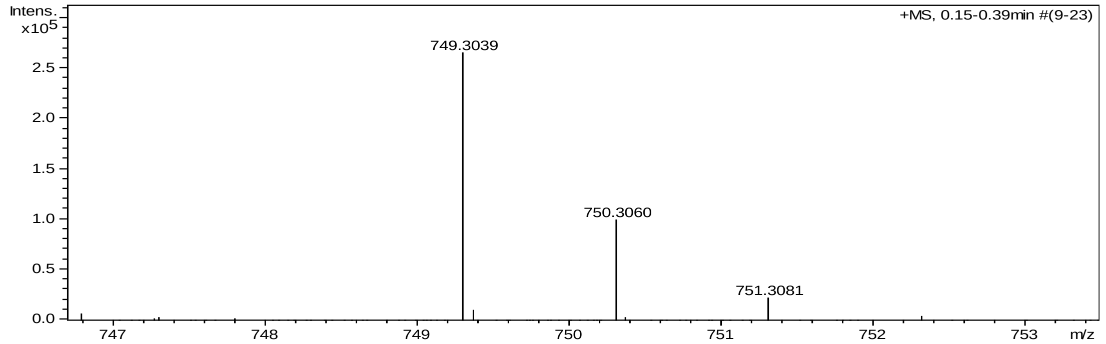
Intens.
x10^5
+MS, 0.15-0.39min #(9-23)
749.3039
750.3060
751.3081
2.5
2.0
1.5
1.0
0.5
0.0
747
748
749
750
751
752
753
m/z

Pentamethyl *rel*-(4*R*,4a*R*,4b*S*,8a*S*)-1-(2-chlorobenzyl)-8-cyclohexyl-2-methyl-4-phenyl-4,4a,8,8a-tetrahydrocyclopenta[4,5]pyrrolo[2,3-*b*]pyridine-3,4b,5,6,7(1*H*)-pentacarboxylate (4h): yellow solid, 80%, m.p. 203-205 °C; ^{1}H NMR (400 MHz, $CDCl_3$) δ 7.52-7.47 (m, 2H, ArH), 7.43 (t, J = 7.6 Hz, 1H, ArH), 7.35 (t, J = 7.6 Hz, 1H, ArH), 7.13 (t, J = 7.2 Hz, 2H, ArH), 7.06-6.99 (m, 3H, ArH), 5.67 (d, J = 3.2 Hz, 1H, CH), 4.99 (d, J = 16.8 Hz, 1H, CH_2), 4.94-4.89 (m, 1H, CH), 4.56 (d, J = 16.4 Hz, 1H, CH_2), 3.96 (s, 3H, OCH_3), 3.75 (s, 3H, OCH_3), 3.55 (s, 3H, OCH_3), 3.42 (dd, J_1 = 8.4 Hz, J_2 = 2.8 Hz, 1H, CH), 3.26 (s, 3H, OCH_3), 3.06 (d, J = 8.4 Hz, 1H, CH), 2.99 (s, 3H, OCH_3), 2.28 (s, 3H, CH_3), 1.93-1.89 (m, 2H, CH_2), 1.83-1.73 (m, 4H, CH_2), 1.55-1.36 (m, 3H, CH_2), 1.23-1.16 (m, 1H, CH_2) ppm. ^{13}C NMR (100 MHz, $CDCl_3$) δ 170.5, 168.0, 167.3, 166.4, 162.4, 161.9, 157.6, 145.6, 143.7, 134.8, 133.0, 130.2, 129.4, 129.1, 128.9, 127.8, 127.5, 126.0, 113.1, 110.6, 110.0, 99.5, 83.2, 77.4, 77.0, 76.7, 72.0, 58.4, 53.4, 53.0, 52.4, 52.2, 51.4, 50.7, 50.5, 38.3, 31.6, 31.4, 26.2, 25.71, 25.65, 17.9 ppm. IR (KBr) υ: 3435, 2949, 2856, 1737, 1695, 1581, 1541, 1435, 1385, 1337, 1240, 1206, 1117, 1091, 999, 978, 895, 854, 790, 755cm^{-1}; MS (*m/z*): HRMS (ESI) Calcd. for $C_{40}H_{43}ClNaN_2O_{10}$ ($[M+Na]^+$): 769.2498, found: 769.2489.

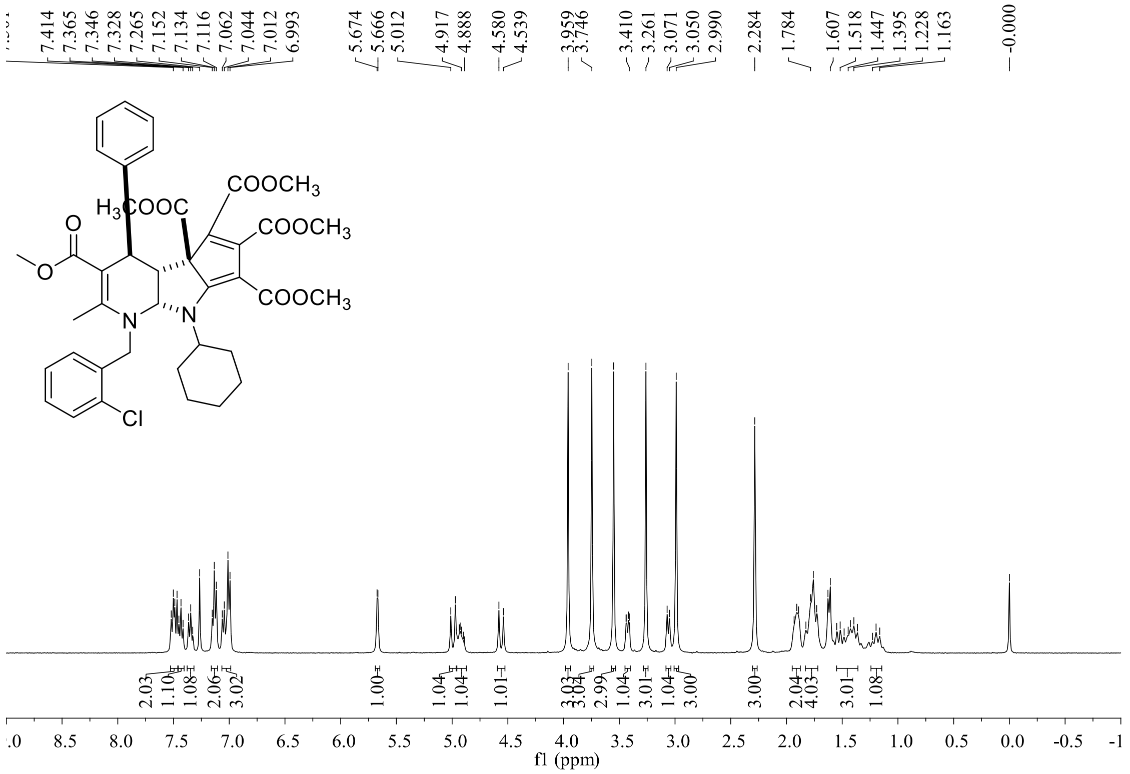

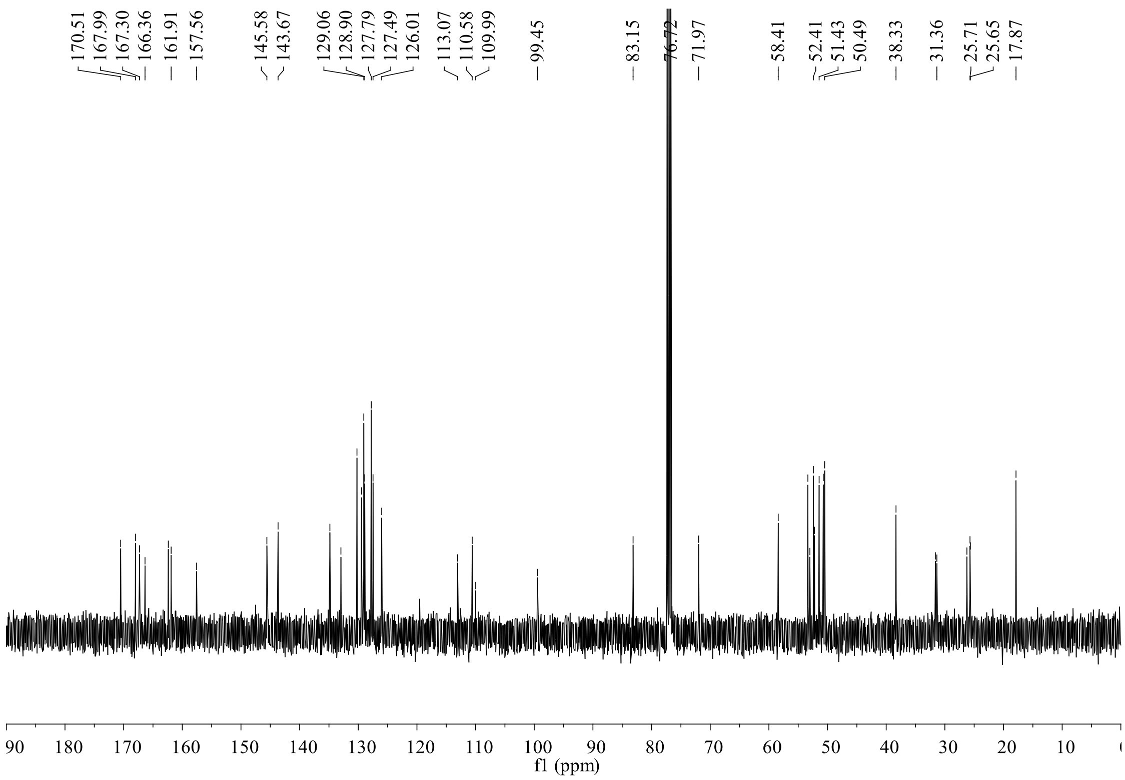
170.51
167.99
167.30
166.36
161.91
157.56
145.58
143.67
129.06
128.90
127.79
127.49
126.01
113.07
110.58
109.99
99.45
83.15
76.72
71.97
58.41
52.41
51.43
50.49
38.33
31.36
25.71
25.65
17.87
f1 (ppm)

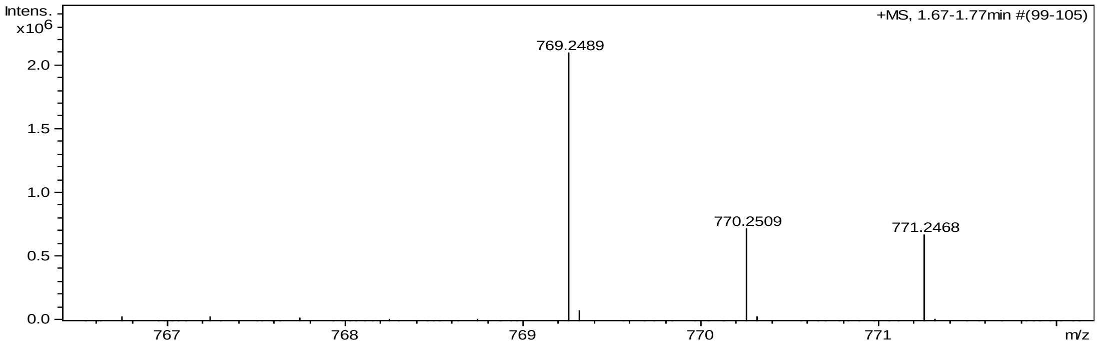
Intens.
x10^6
+MS, 1.67-1.77min #(99-105)
769.2489
770.2509
771.2468
m/z

Pentamethyl *rel*-(4*R*,4a*R*,4b*S*,8a*S*)-8-cyclohexyl-1-(3-methoxybenzyl)-2-methyl-4-phenyl-4,4a,8,8a-tetrahydrocyclopenta[4,5]pyrrolo[2,3-*b*]pyridine-3,4b,5,6,7(1*H*)-pentacarboxylate (4i): yellow solid, 80%, m.p. 201-203 °C; ^{1}H NMR (400 MHz, $CDCl_3$) δ 7.39 (t, *J* = 7.6 Hz, 1H, ArH), 7.06 (t, *J* = 6.8 Hz, 2H, ArH), 7.01-6.98 (m, 2H, ArH), 6.97-6.93 (m, 2H, ArH), 6.77 (d, *J* = 7.2 Hz, 2H, ArH), 5.81 (d, *J* = 3.6 Hz, 1H, CH), 4.94-4.88 (m, 1H, CH), 4.85 (d, *J* = 15.6 Hz, 1H, CH_2), 4.27 (d, *J* = 15.6 Hz, 1H, CH_2), 3.96 (s, 3H, OCH_3), 3.87 (s, 3H, OCH_3), 3.74 (s, 3H, OCH_3), 3.57 (s, 3H, OCH_3), 3.34 (dd, J_1 = 8.4 Hz, J_2 = 3.2 Hz, 1H, CH), 3.26 (s, 3H, OCH_3), 3.00-2.97 (m, 1H, CH), 2.96 (s, 3H, OCH_3), 2.33 (s, 3H, CH_3), 1.92-1.85 (m, 2H, CH_2), 1.79-1.72 (m, 4H, CH_2), 1.48-1.35 (m, 3H, CH_2), 1.20-1.13 (m, 1H, CH_2) ppm. ^{13}C NMR (100 MHz, $CDCl_3$) δ 170.2, 167.8, 167.5, 166.4, 162.4, 161.9, 160.2, 157.6, 145.6, 143.9, 138.7, 130.2, 128.9, 127.7, 125.8, 120.3, 114.0, 113.4, 113.0, 112.0, 99.2, 83.8, 72.1, 58.4, 56.6, 55.3, 53.3, 52.4, 52.3, 51.4, 50.6, 50.4, 38.2, 31.8, 31.1, 26.2, 25.7, 25.6, 18.4 ppm. IR (KBr) υ: 3446, 2949, 2855, 1733, 1697, 1582, 1542, 1435, 1382, 1347, 1231, 1205, 1113, 1094, 1045, 1001, 893, 867, 789 cm^{-1} (*m*/*z*): HRMS (ESI) Calcd. for $C_{41}H_{46}NaN_2O_{11}$ ([M+Na]$^{+}$): 765.2994, found: 765.2992.

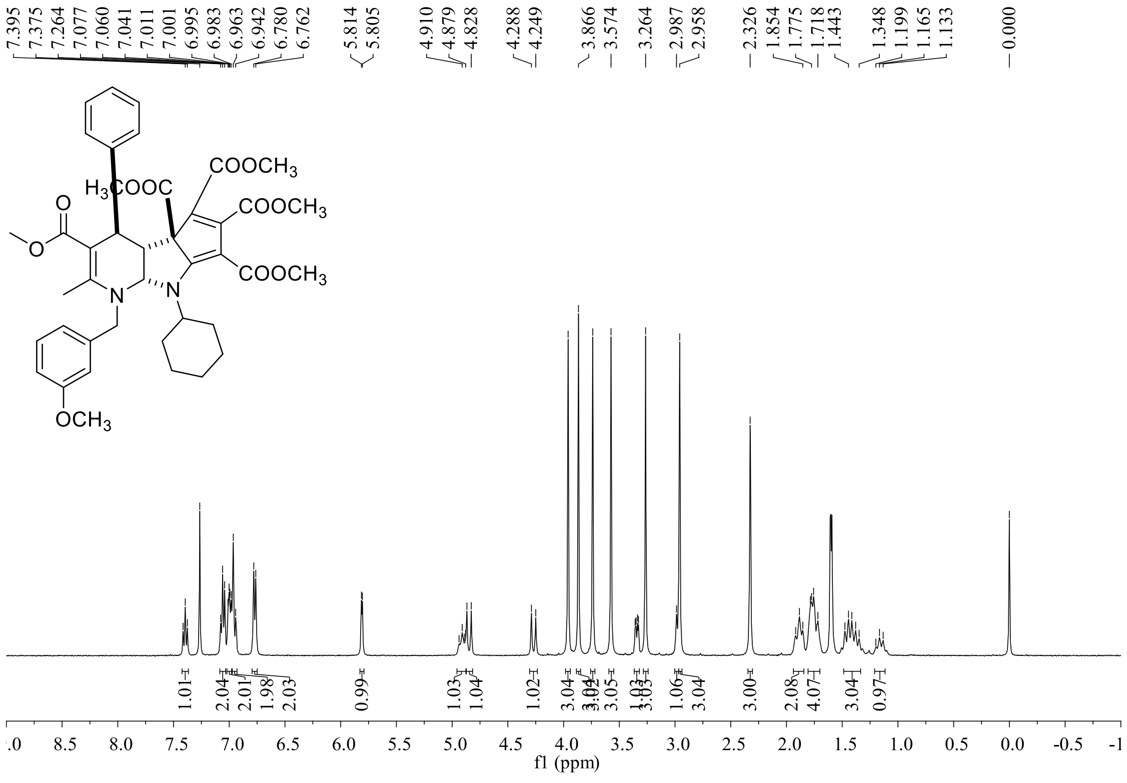

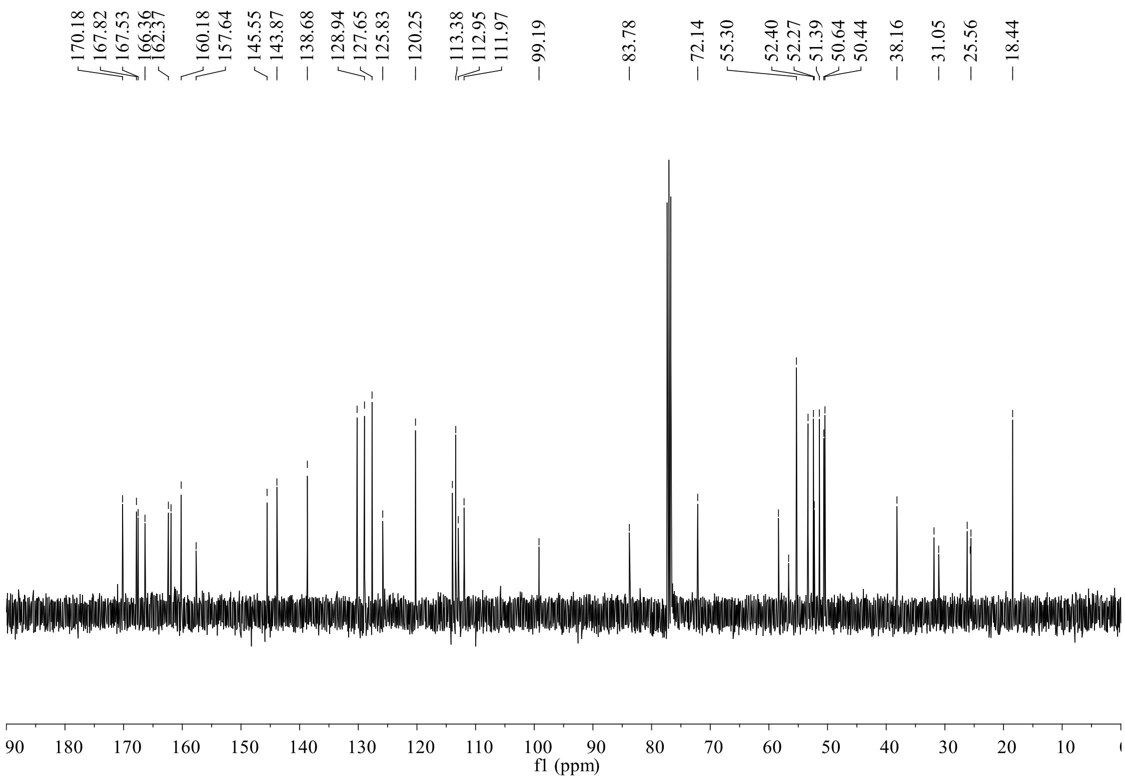
170.18
167.82
167.53
166.36
162.37
160.18
157.64
145.55
143.87
138.68
128.94
127.65
125.83
120.25
113.38
112.95
111.97
99.19
83.78
72.14
55.30
52.40
52.27
51.39
50.64
50.44
38.16
31.05
25.56
18.44
f1 (ppm)

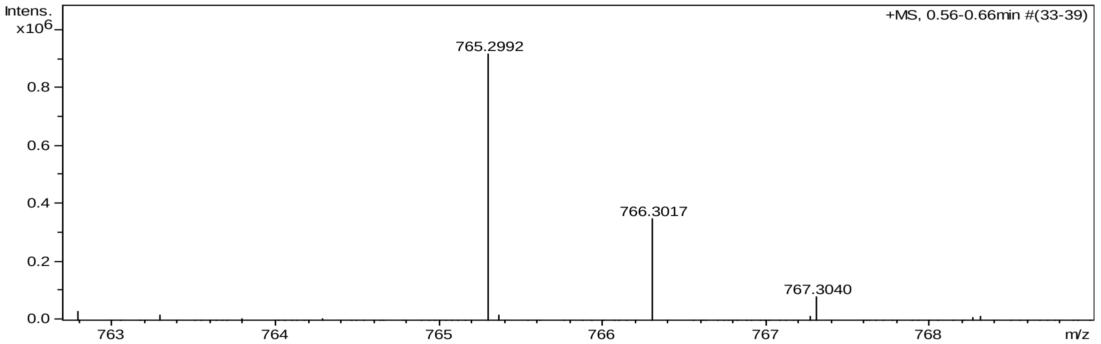
Intens.
x10^6
+MS, 0.56-0.66min #(33-39)
765.2992
766.3017
767.3040
m/z

Pentamethyl *rel*-(4*R*,4a*R*,4b*S*,8a*R*)-8-cyclohexyl-1-(4-methoxyphenyl)-2-methyl-4-phenyl-4,4a,8,8a-tetrahydrocyclopenta[4,5]pyrrolo[2,3-*b*]pyridine-3,4b,5,6,7(1*H*)-pentacarboxylate (4j): yellow solid, 87%, m.p. 144-146 ºC; ^{1}H NMR (400 MHz, $CDCl_3$) δ 7.30-7.27 (m, 1H, ArH), 7.25-7.19 (m, 3H, ArH), 7.15 (d, J = 7.2 Hz, 2H, ArH), 7.11-7.07 (m, 1H, ArH), 6.98 (d, J = 8.4 Hz, 2H, ArH), 5.85 (s, 1H, CH), 4.72 (t, J = 12.8 Hz, 1H, CH), 3.98 (s, 3H, OCH_3), 3.86 (s, 3H, OCH_3), 3.76 (s, 3H, OCH_3), 3.71-3.67 (m, 1H, CH), 3.55 (s, 3H, OCH_3), 3.37 (s, 3H, OCH_3), 3.15 (d, J = 7.6 Hz, 1H, CH), 3.01 (s, 3H, OCH_3), 2.05 (s, 3H, CH_3), 2.02-1.95 (m, 1H, CH_2), 1.90-1.83 (m, 3H, CH_2), 1.72-1.63 (m, 3H, CH_2), 1.41-1.31 (m, 2H, CH_2), 1.24-1.20 (m, 1H, CH_2) ppm. ^{13}C NMR (100 MHz, $CDCl_3$) δ 170.1, 167.6, 167.4, 166.3, 162.4, 161.9, 158.0, 157.8, 145.8, 143.8, 138.6, 129.0, 128.0, 126.8, 126.1, 114.9, 114.3, 113.1, 100.0, 85.5, 72.4, 58.6, 55.6, 53.4, 52.4, 51.8, 51.4, 50.7, 38.5, 30.8, 26.1, 25.9, 25.3, 20.5 ppm. IR (KBr) υ: 3434, 2949, 1737, 1694, 1581, 1542, 1509, 1434, 1339, 1231, 1204, 1096, 1067, 1033, 1001, 825, 794, 761 cm^{-1}; MS (m/z): HRMS (ESI) Calcd. for $C_{40}H_{44}NaN_2O_{11}$ ([M+Na]$^+$): 751.2837, found: 751.2840.

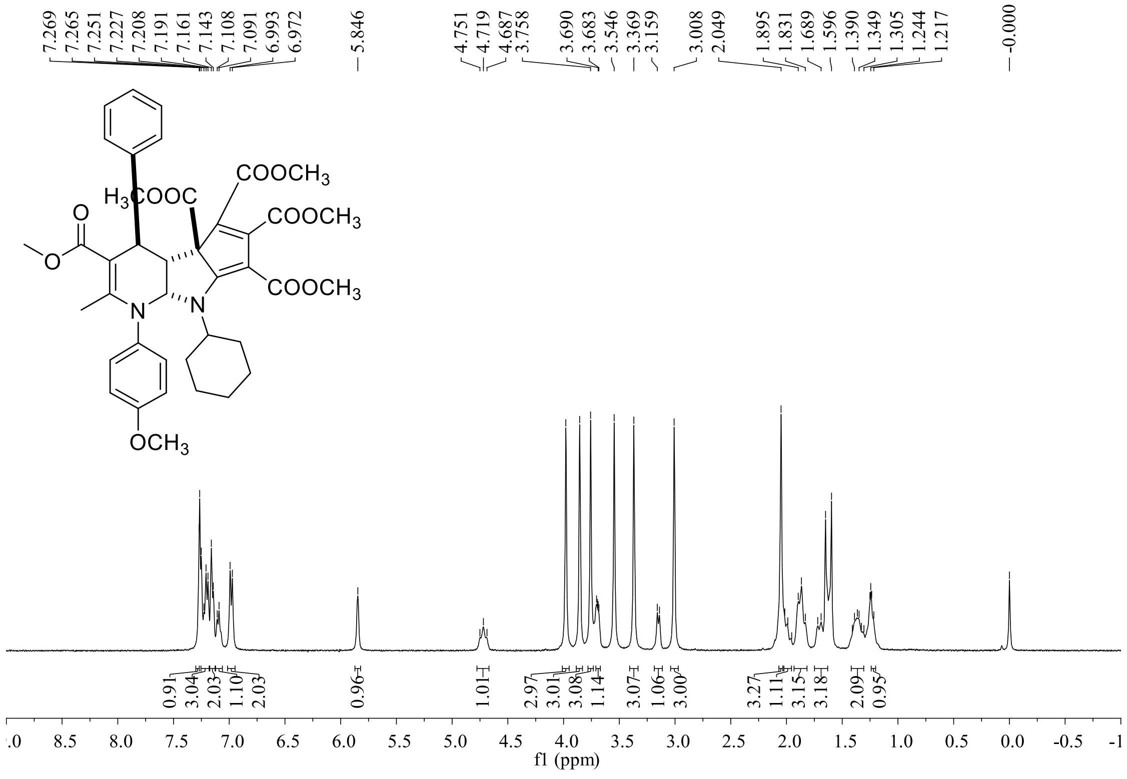

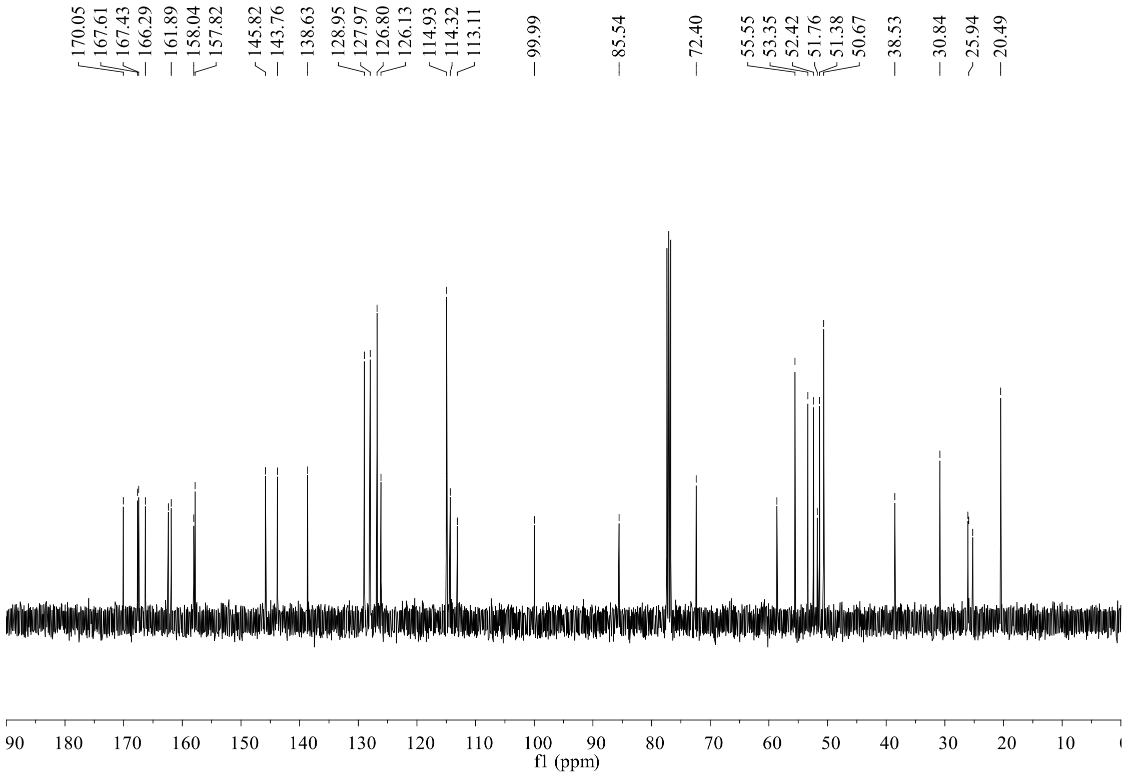
170.05
167.61
167.43
166.29
161.89
158.04
157.82
145.82
143.76
138.63
128.95
127.97
126.80
126.13
114.93
114.32
113.11
99.99
85.54
72.40
55.55
53.35
52.42
51.76
51.38
50.67
38.53
30.84
25.94
20.49
90 180 170 160 150 140 130 120 110 100 90 80 70 60 50 40 30 20 10
f1 (ppm)

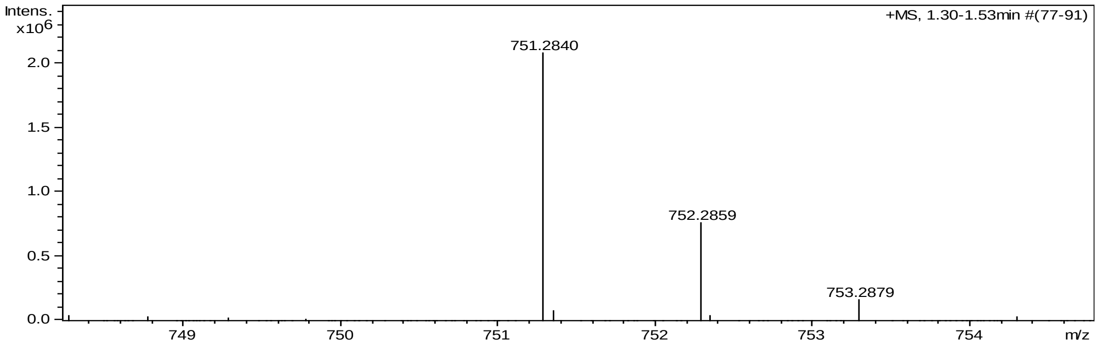
Intens.
x10^6
+MS, 1.30-1.53min #(77-91)
751.2840
752.2859
753.2879
2.0
1.5
1.0
0.5
0.0
749
750
751
752
753
754
m/z

Pentamethyl *rel*-(4*R*,4a*R*,4b*S*,8a*R*)-1-(4-bromophenyl)-8-cyclohexyl-2-methyl-4-phenyl-4,4a,8,8a-tetrahydrocyclopenta[4,5]pyrrolo[2,3-*b*]pyridine-3,4b,5,6,7(1*H*)-pentacarboxylate (4k): yellow solid, 93%, m.p. 134-136 °C; ^{1}H NMR (400 MHz, $CDCl_3$) δ 7.55 (d, *J* = 8.8 Hz, 2H, ArH), 7.21-7.17 (m, 2H, ArH), 7.11-7.07 (m, 5H, ArH), 6.05 (d, *J* = 4.0 Hz, 1H, CH), 4.72-4.66 (m, 1H, CH), 3.98 (s, 3H, OCH_3), 3.77-3.74 (m, 4H, CH, OCH_3), 3.58 (s, 3H, OCH_3), 3.46 (s, 3H, OCH_3), 3.26 (d, *J* = 6.4 Hz, 1H, CH), 3.08 (s, 3H, OCH_3), 2.10 (s, 3H, CH_3), 1.98-1.86 (m, 4H, CH_2), 1.84-1.80 (m, 1H, CH_2), 1.71-1.68 (m, 2H, CH_2), 1.39-1.33 (m, 2H, CH_2), 1.22-1.16 (m, 1H, CH_2) ppm. ^{13}C NMR (100 MHz, $CDCl_3$) δ 169.9, 167.9, 167.1, 166.1, 162.3, 161.9, 157.9, 145.1, 143.9, 143.4, 132.8, 128.6, 128.1, 126.3, 124.9, 118.6, 118.0, 113.6, 100.2, 85.5, 72.7, 58.9, 53.5, 52.4, 52.2, 51.4, 51.0, 50.8, 38.6, 31.3, 31.0, 26.1, 25.9, 25.2, 20.9 ppm. IR (KBr) υ: 3434, 2949, 2857, 1735, 1698, 1584, 1545, 1489, 1435, 1342, 1235, 1153, 1098, 1071, 1009, 822, 795 cm^{-1}; MS (*m*/*z*): HRMS (ESI) Calcd. for $C_{39}H_{41}BrNaN_2O_{10}$ ($[M+Na]^+$): 800.6542, found: 800.1854.

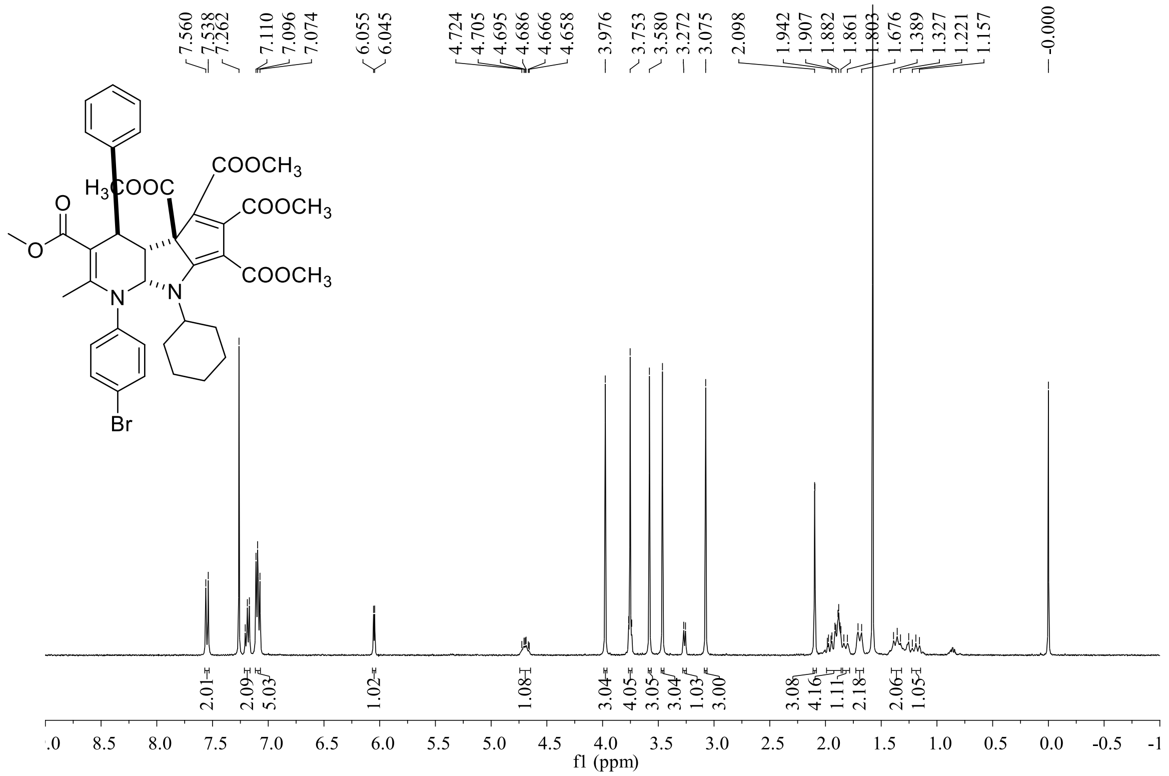

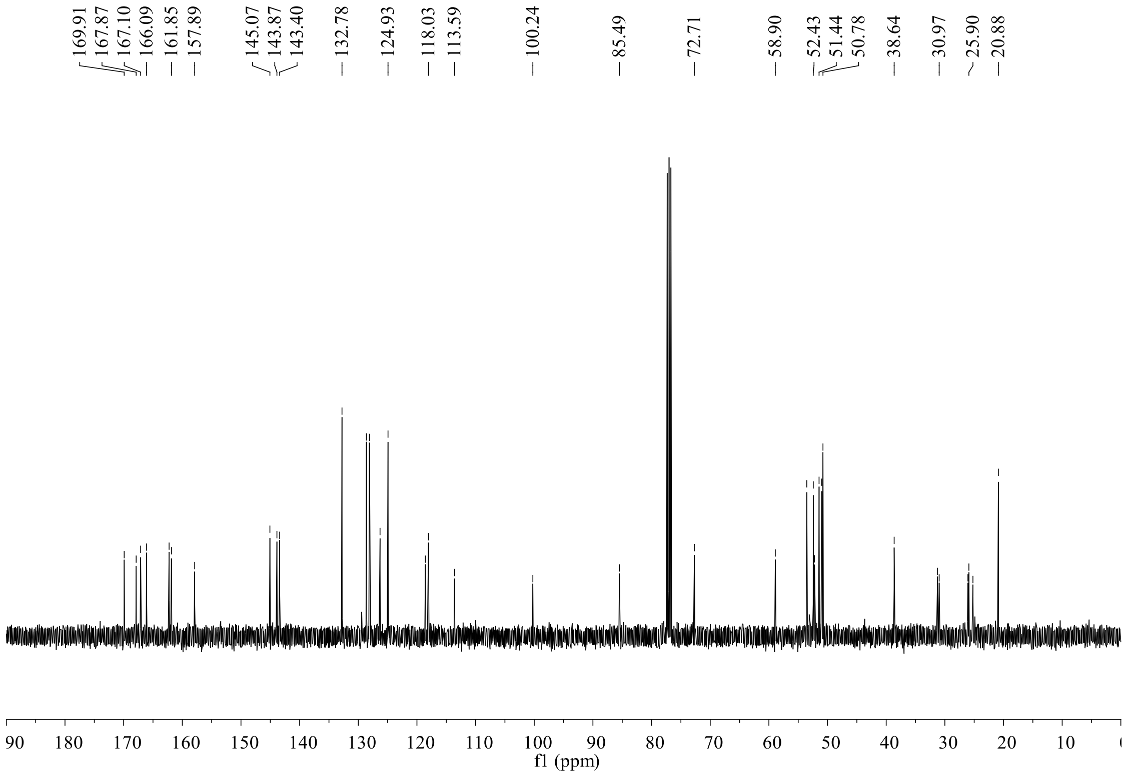
169.91
167.87
167.10
166.09
161.85
157.89
145.07
143.87
143.40
132.78
124.93
118.03
113.59
100.24
85.49
72.71
58.90
52.43
51.44
50.78
38.64
30.97
25.90
20.88
90 180 170 160 150 140 130 120 110 100 90 80 70 60 50 40 30 20 10
f1 (ppm)

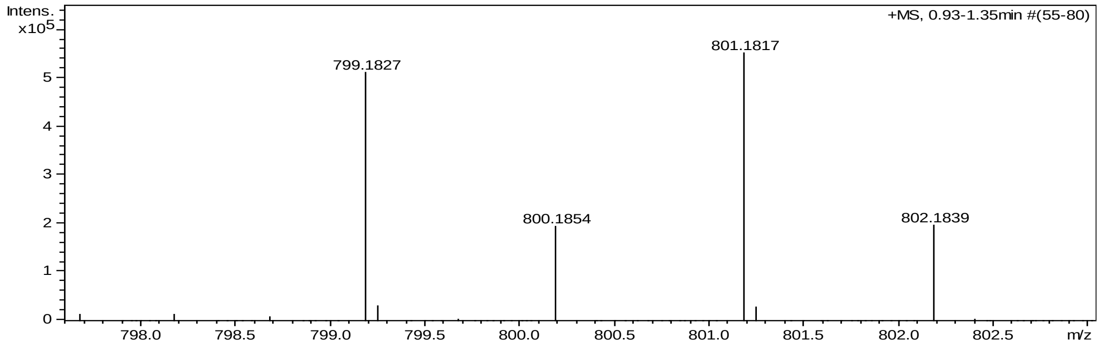
Intens.
x10^5
+MS, 0.93-1.35min #(55-80)
799.1827
800.1854
801.1817
802.1839
0
1
2
3
4
5
798.0 798.5 799.0 799.5 800.0 800.5 801.0 801.5 802.0 802.5 m/z

Pentamethyl *rel*-(4*R*,4a*R*,4b*S*,8a*R*)-1-(3-chlorophenyl)-8-cyclohexyl-2-methyl-4-phenyl-4,4a,8,8a-tetrahydrocyclopenta[4,5]pyrrolo[2,3-*b*]pyridine-3,4b,5,6,7(1*H*)-pentacarboxylate (4l): yellow solid, 92%, m.p. 184-186 °C; ^{1}H NMR (400 MHz, $CDCl_3$) δ 7.37-7.33 (m, 1H, ArH), 7.21-7.15 (m, 4H, ArH), 7.12-7.05 (m, 4H, ArH), 6.08 (d, *J* = 3.2 Hz, 1H, CH), 4.70-4.65 (m, 1H, CH), 3.98 (s, 3H, OCH_3), 3.81-3.78 (m, 1H, CH), 3.75 (s, 3H, OCH_3), 3.59 (s, 3H, OCH_3), 3.48 (s, 3H, OCH_3), 3.31 (d, *J* = 5.2 Hz, 1H, CH), 3.10 (s, 3H, OCH_3), 2.11 (s, 3H, CH_3), 1.99-1.81 (m, 6H, CH_2), 1.71-1.69 (m, 2H, CH_2), 1.39-1.33 (m, 2H, CH_2) ppm. ^{13}C NMR (100 MHz, $CDCl_3$) δ 169.9, 168.0, 167.1, 166.1, 162.3, 161.9, 157.9, 145.8, 145.2, 143.3, 135.3, 130.6, 128.6, 128.1, 126.3, 124.9, 123.3, 121.2, 119.1, 113.7, 100.3, 85.4, 77.4, 77.0, 76.7, 72.8, 59.0, 53.6, 52.5, 52.3, 51.5, 51.1, 50.9, 38.6, 31.3, 30.9, 26.1, 25.9, 25.2, 21.0 ppm. IR (KBr) υ: 3434, 2949, 2855, 1734, 1702, 1589, 1546, 1479, 1435, 1342, 1312, 1229, 1153, 1098, 1067, 1000, 751 cm^{-1}; MS (*m*/*z*): HRMS (ESI) Calcd. for $C_{39}H_{41}ClNaN_2O_{10}$ ($[M+Na]^+$): 755.2342, found: 755.2339.

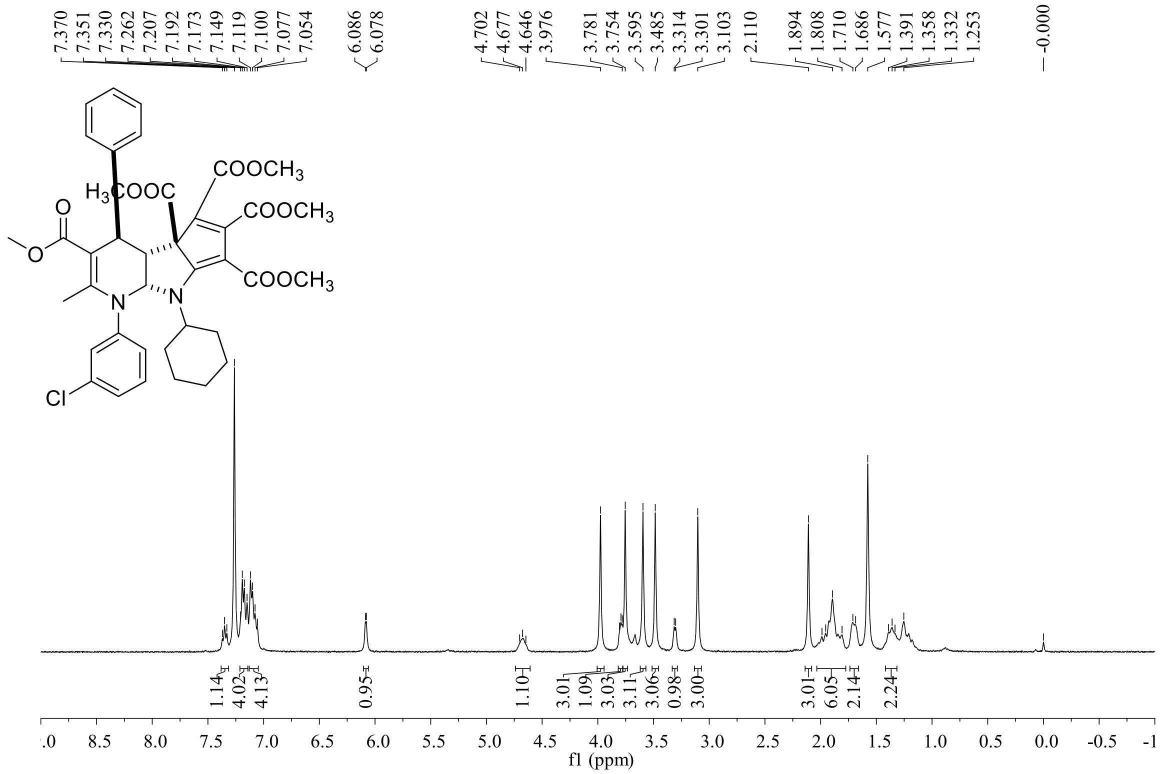

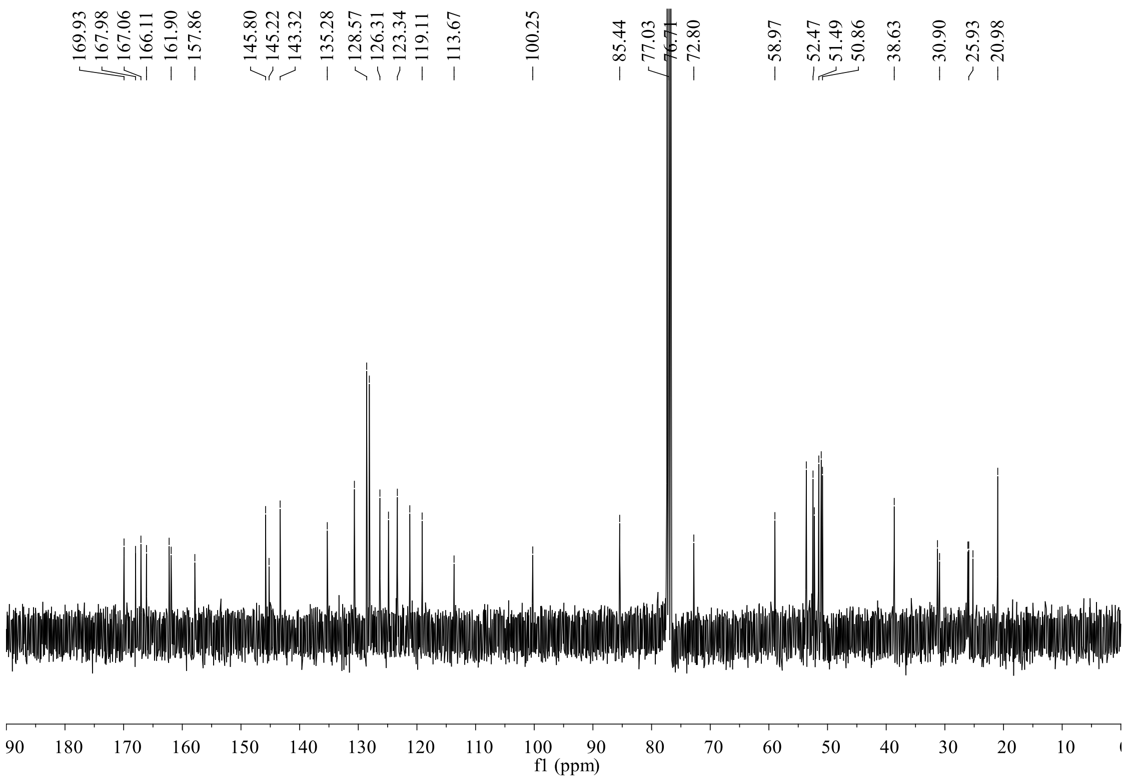
169.93
167.98
167.06
166.11
161.90
157.86
145.80
145.22
143.32
135.28
128.57
126.31
123.34
119.11
113.67
100.25
85.44
77.03
76.71
72.80
58.97
52.47
51.49
50.86
38.63
30.90
25.93
20.98
90 180 170 160 150 140 130 120 110 100 90 80 70 60 50 40 30 20 10
f1 (ppm)

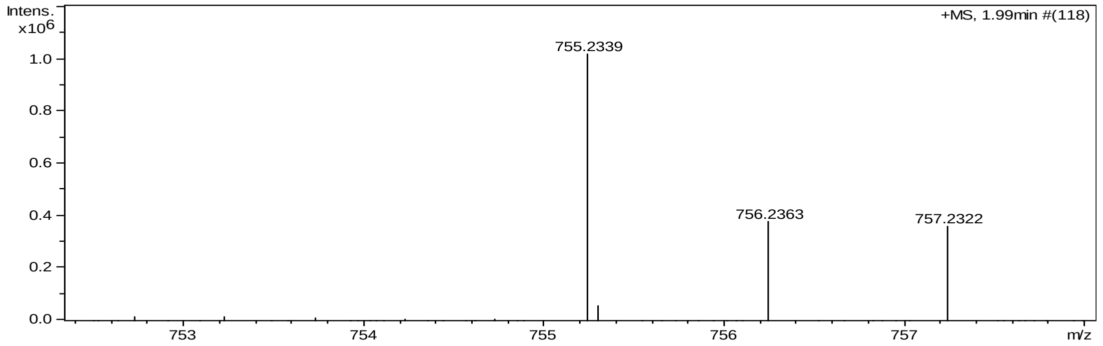
Intens.
x10^6
+MS, 1.99min #(118)
755.2339
756.2363
757.2322
1.0
0.8
0.6
0.4
0.2
0.0
753 754 755 756 757
m/z

Pentamethyl *rel*-(4*R*,4a*R*,4b*S*,8a*R*)-8-cyclohexyl-4-(2-methoxyphenyl)-2-methyl-1-(*o*-tolyl)-4,4a,8,8a-tetrahydrocyclopenta[4,5]pyrrolo[2,3-*b*]pyridine-3,4b,5,6,7(1*H*)-pentacarboxylate (4m): yellow solid, 93%, m.p. 183-185 °C; ^{1}H NMR (400 MHz, $CDCl_3$) δ 7.43-7.27 (m, 4H, ArH), 7.17-7.14 (m, 1H, ArH), 7.10-7.06 (m, 1H, ArH), 6.82-6.75 (m, 2H, ArH), 5.59 (d, *J* = 3.2 Hz, 1H, CH), 4.14-4.09 (m, 2H, CH), 3.95 (s, 3H, OCH_3), 3.78 (s, 3H, OCH_3), 3.76 (s, 3H, OCH_3), 3.53 (s, 3H, OCH_3), 3.41-3.34 (m, 1H, CH), 3.20 (s, 3H, OCH_3), 2.98 (s, 3H, OCH_3), 2.58 (s, 3H, CH_3), 2.49-2.42 (m, 1H, CH_2), 2.24-2.14 (m, 1H, CH_2), 1.88 (s, 3H, CH_3), 1.87-1.83 (m, 2H, CH_2), 1.62-1.56 (m, 3H, CH_2), 1.39-1.27 (m, 3H, CH_2) ppm. ^{13}C NMR (100 MHz, $CDCl_3$) δ 168.6, 167.6, 166.5, 162.6, 161.8, 157.3, 144.1, 143.1, 137.7, 132.2, 130.4, 130.2, 128.1, 127.6, 126.3, 120.1, 114.0, 110.6, 100.5, 85.9, 72.9, 58.3, 57.5, 55.4, 53.3, 52.2, 51.2, 50.6, 50.3, 29.9, 29.4, 26.14, 26.09, 24.4, 18.94, 18.86, 18.4 ppm. IR (KBr) υ: 3435, 2947, 2850, 1734, 1702, 1584, 1542, 1434, 1398, 1337, 1232, 1200, 1118, 1091, 1064, 949, 852, 795, 754 cm^{-1}; MS (*m*/*z*): HRMS (ESI) Calcd. for $C_{41}H_{46}NaN_2O_{11}$ ($[M+Na]^+$): 765.2994, found: 765.2995.

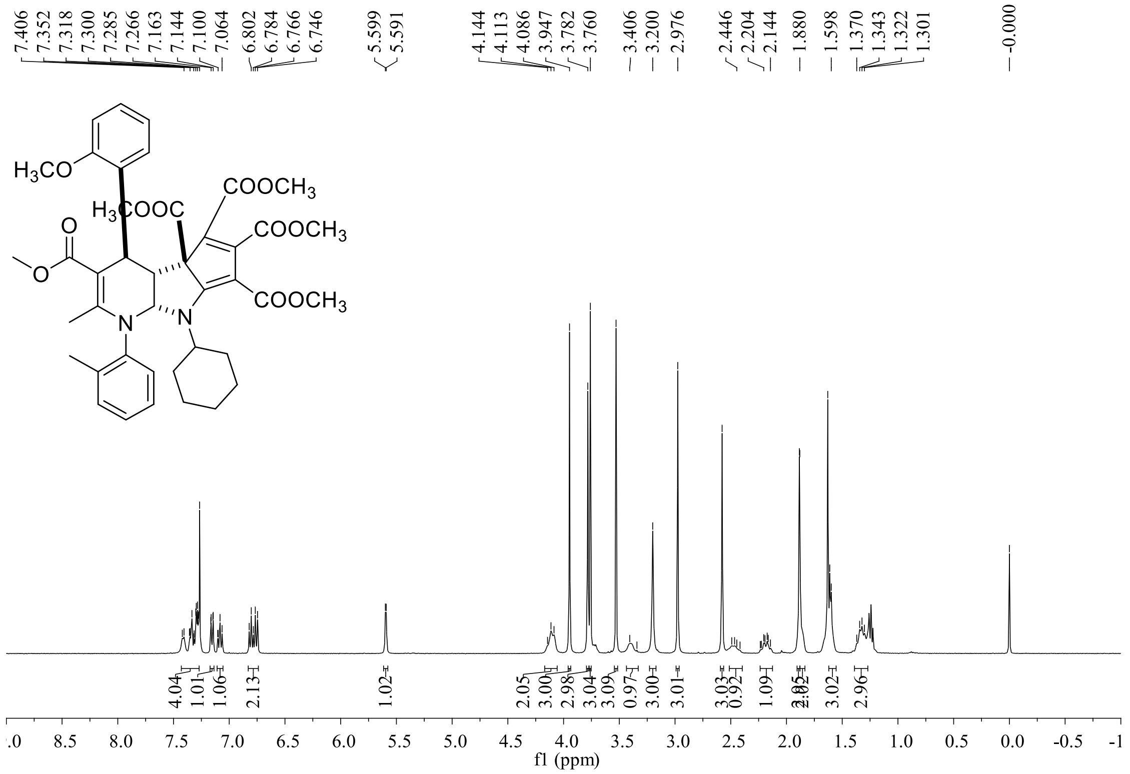

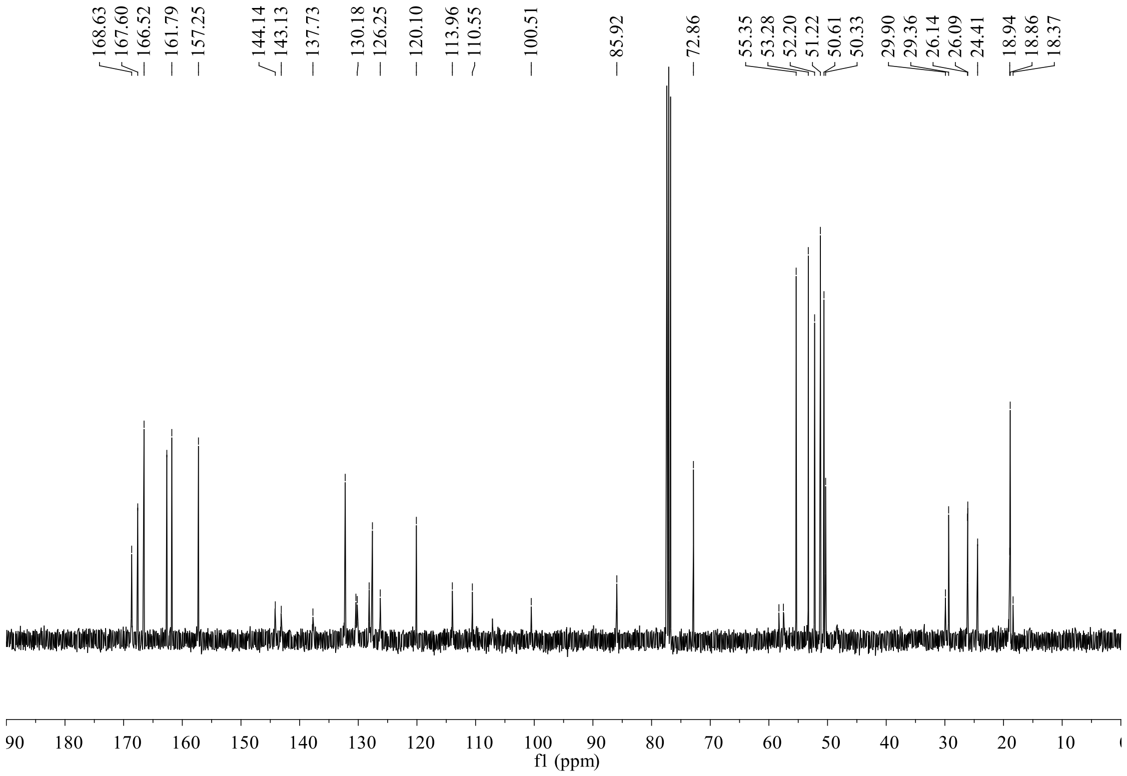
168.63
167.60
166.52
161.79
157.25
144.14
143.13
137.73
130.18
126.25
120.10
113.96
110.55
100.51
85.92
72.86
55.35
53.28
52.20
51.22
50.61
50.33
29.90
29.36
26.14
26.09
24.41
18.94
18.86
18.37
f1 (ppm)

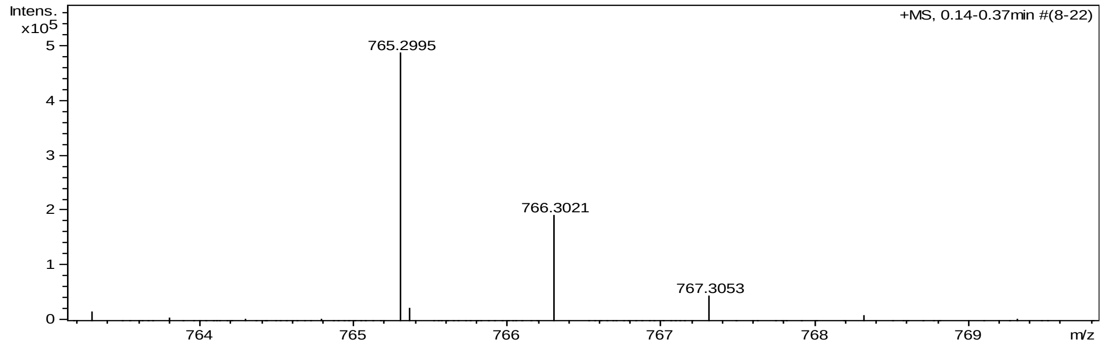
Intens.
x10^5
+MS, 0.14-0.37min #(8-22)
765.2995
766.3021
767.3053
m/z

Pentamethyl *rel*-(4*R*,4a*R*,4b*S*,8a*S*)-1-butyl-8-cyclohexyl-4-phenyl-2-methyl-4,4a,8,8a-tetrahydrocyclopenta[4,5]pyrrolo[2,3-*b*]pyridine-3,4b,5,6,7(1*H*)-pentacarboxylate (4n): yellow solid, 80%, m.p. 177-179 °C; ^{1}H NMR (400 MHz, $CDCl_3$) δ 7.17 (t, *J* = 7.6 Hz, 2H, ArH), 7.08-7.02 (m, 3H, ArH), 5.58 (d, *J* = 3.6 Hz, 1H, CH), 4.90 (d, *J* = 10.4 Hz, 1H, CH), 3.97 (s, 3H, OCH_3), 3.78-3.69 (m, 4H, CH_2, OCH_3), 3.60 (s, 3H, OCH_3), 3.48 (dd, J_1 = 9.2 Hz, J_2 = 3.6 Hz, 1H, CH), 3.26 (s, 3H, OCH_3), 3.08-3.01 (m, 2H, CH, CH_2), 2.99 (s, 3H, OCH_3), 2.23 (s, 3H, CH_3), 1.88-1.79 (m, 2H, CH_2), 1.73-1.63 (m, 6H, CH_2), 1.49-1.31 (m, 5H, CH_2), 1.14-1.09 (m, 1H, CH_2), 1.04 (t, *J* = 7.2 Hz, 3H, CH_3) ppm. ^{13}C NMR (100 MHz, $CDCl_3$) δ 170.0, 168.1, 167.7, 166.4, 162.4, 162.0, 157.7, 145.6, 144.0, 129.1, 127.8, 126.0, 112.8, 110.8, 98.9, 83.7, 71.9, 58.2, 53.4, 53.3, 52.9, 52.4, 51.4, 50.7, 50.4, 38.2, 31.71, 31.67, 30.9, 26.21, 26.19, 25.7, 25.4, 20.1, 17.8, 13.8 ppm. IR (KBr) υ: 3434, 2946, 2857, 2360, 1737, 1691, 1584, 1547, 1434, 1379, 1337, 1306, 1227, 1206, 1111, 1082, 1001, 756 cm^{-1}; MS (*m*/*z*): HRMS (ESI) Calcd. for $C_{37}H_{46}NaN_2O_{10}$ ($[M+Na]^+$): 701.3045, found: 701.3049.

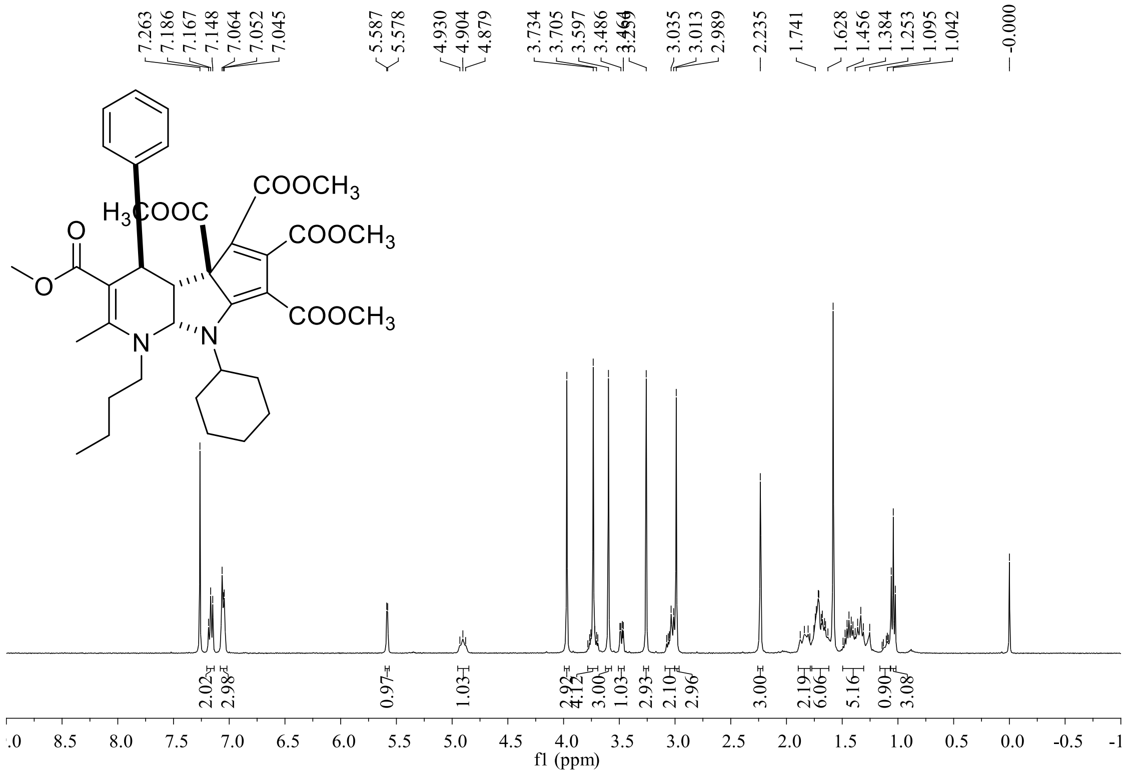

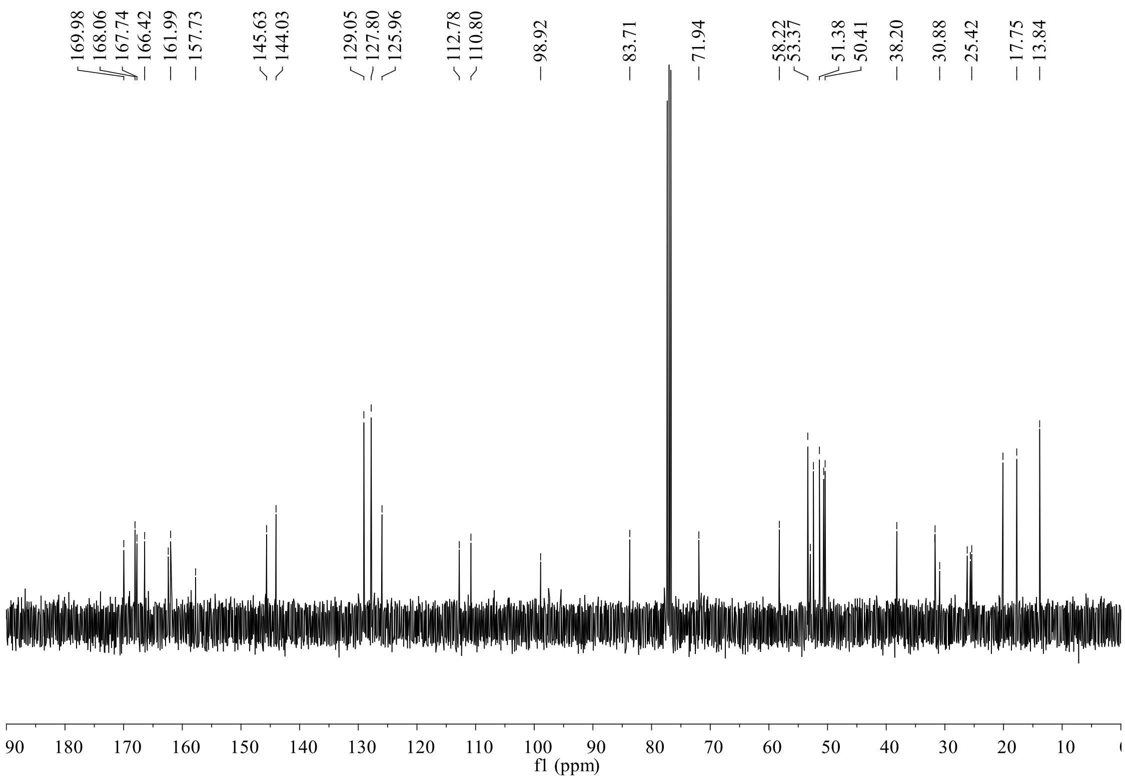
169.98
168.06
167.74
166.42
161.99
157.73
145.63
144.03
129.05
127.80
125.96
112.78
110.80
98.92
83.71
71.94
58.22
53.37
51.38
50.41
38.20
30.88
25.42
17.75
13.84
f1 (ppm)

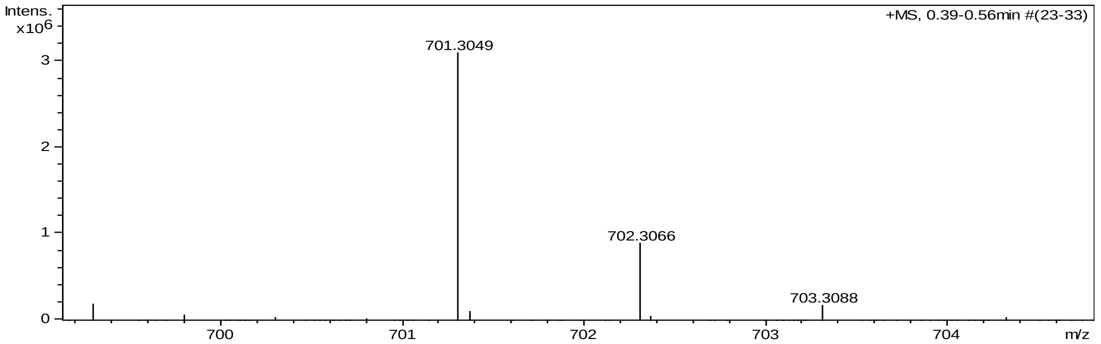
Intens.
x10^6
+MS, 0.39-0.56min #(23-33)
701.3049
702.3066
703.3088
m/z

3-Ethyl 4b,5,6,7-tetramethyl *rel*-(4*R*,4a*R*,4b*S*,8a*S*)-1-benzyl-8-cyclohexyl-2-methyl-4-phenyl-4,4a,8,8a-tetrahydrocyclopenta[4,5]pyrrolo[2,3-*b*]pyridine-3,4b,5,6,7(1*H*)-pentacarboxylate (4o): yellow solid, 79%, m.p. 204-206 °C; ^{1}H NMR (400 MHz, $CDCl_3$) δ 7.51-7.47 (m, 2H, ArH), 7.43-7.39 (m, 3H, ArH), 7.06-6.97 (m, 3H, ArH), 6.74 (d, *J* = 7.2 Hz, 2H, ArH), 5.81 (d, *J* = 3.2 Hz, 1H, CH), 4.90-4.86 (m, 2H, CH, CH_2), 4.30 (d, *J* = 15.6 Hz, 1H, CH_2), 3.96 (s, 3H, OCH_3), 3.79-3.75 (m, 2H, CH_2) 3.74 (s, 3H, OCH_3), 3.58 (s, 3H, OCH_3), 3.30 (dd, J_1 = 8.4 Hz, J_2 = 3.6 Hz, 1H, CH), 3.04-3.01 (m, 1H, CH), 3.00 (s, 3H, OCH_3), 2.35 (s, 3H, CH_3), 1.91-1.88 (m, 2H, CH_2), 1.80-1.71 (m, 4H, CH_2), 1.51-1.35 (m, 3H, CH_2), 1.17-1.11 (m, 1H, CH_2), 0.88 (t, *J* = 7.2 Hz, 3H, CH_3) ppm. ^{13}C NMR (100 MHz, $CDCl_3$) δ 170.3, 167.7, 167.4, 166.4, 162.4, 162.0, 157.7, 145.8, 144.0, 137.3, 129.2, 129.1, 128.2, 128.1, 127.6, 125.8, 113.0, 112.0, 99.3, 83.8, 72.1, 59.6, 58.5, 56.6, 53.3, 52.5, 52.4, 51.4, 50.7, 38.1, 31.9, 31.1, 26.2, 25.7, 25.6, 18.5, 13.6 ppm. IR (KBr) υ: 3435, 2949, 2860, 1736, 1698, 1583, 1542, 1434, 1386, 1338, 1236, 1204, 1120, 1091, 1002, 980, 894, 852, 790 cm^{-1}; MS (*m/z*): HRMS (ESI) Calcd. for $C_{41}H_{46}NaN_2O_{10}$ ([M+Na]$^{+}$): 749.3045, found: 749.3042.

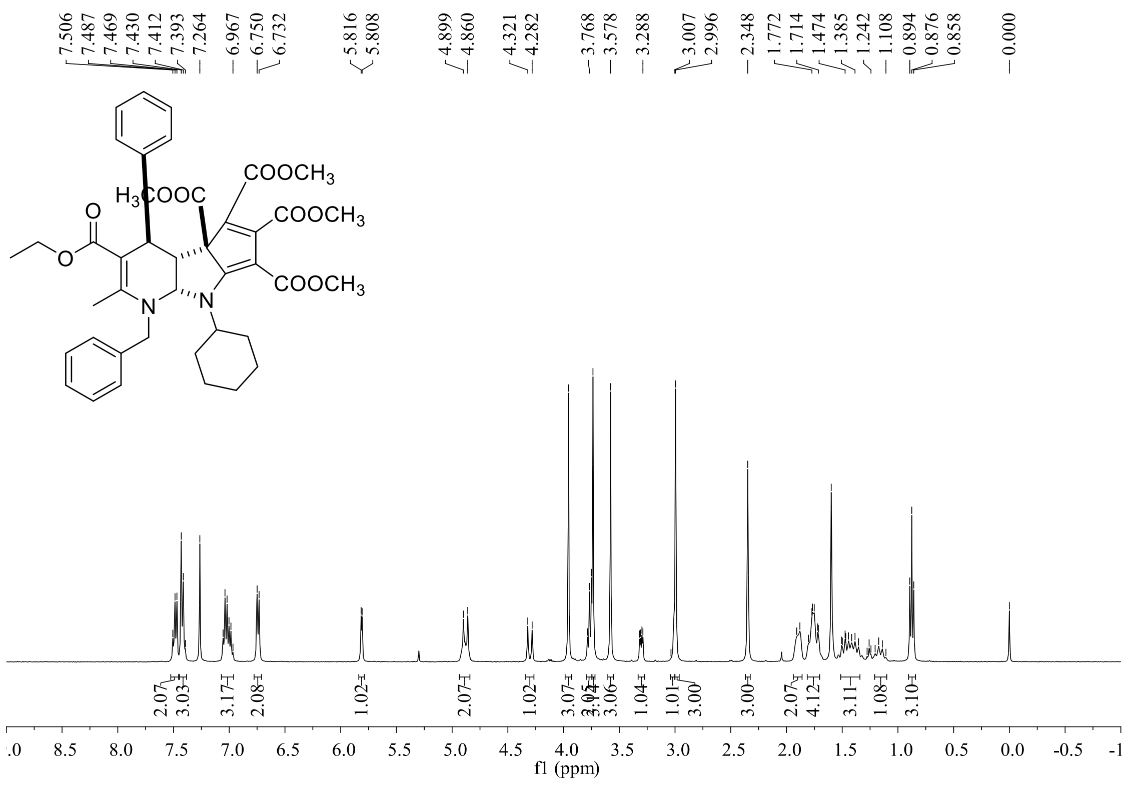

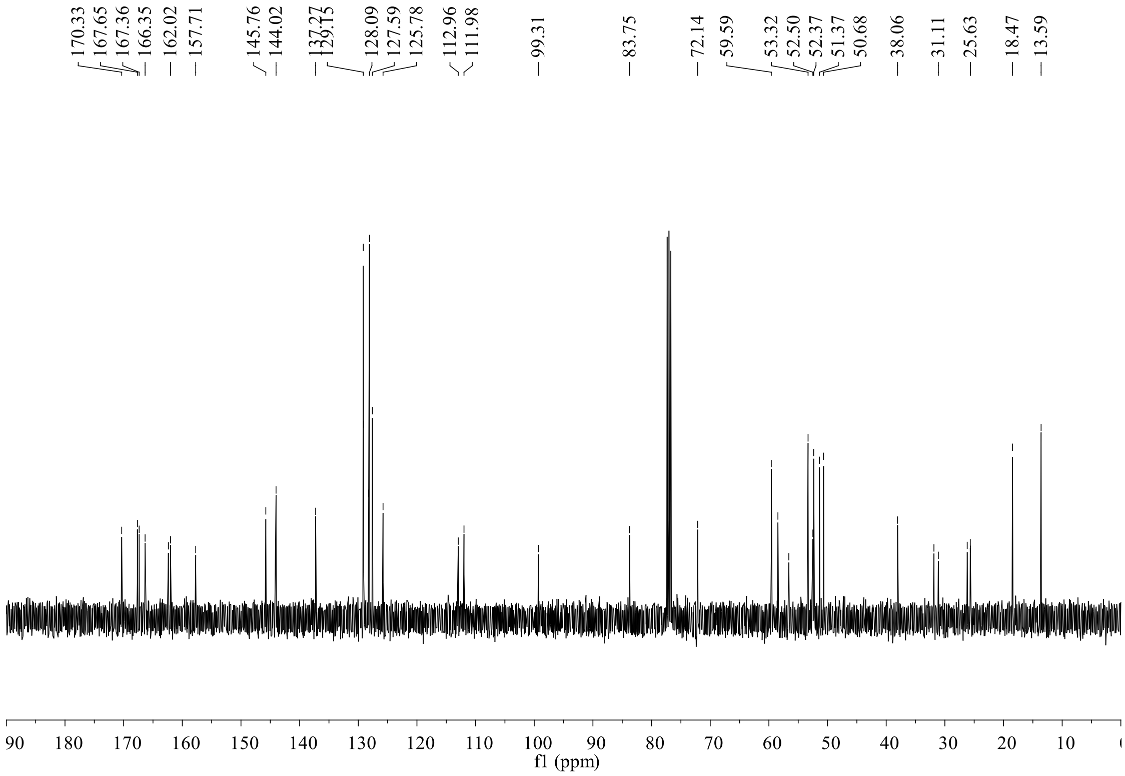
170.33
167.65
167.36
166.35
162.02
157.71
145.76
144.02
137.27
129.15
128.09
127.59
125.78
112.96
111.98
99.31
83.75
72.14
59.59
53.32
52.50
52.37
51.37
50.68
38.06
31.11
25.63
18.47
13.59
f1 (ppm)

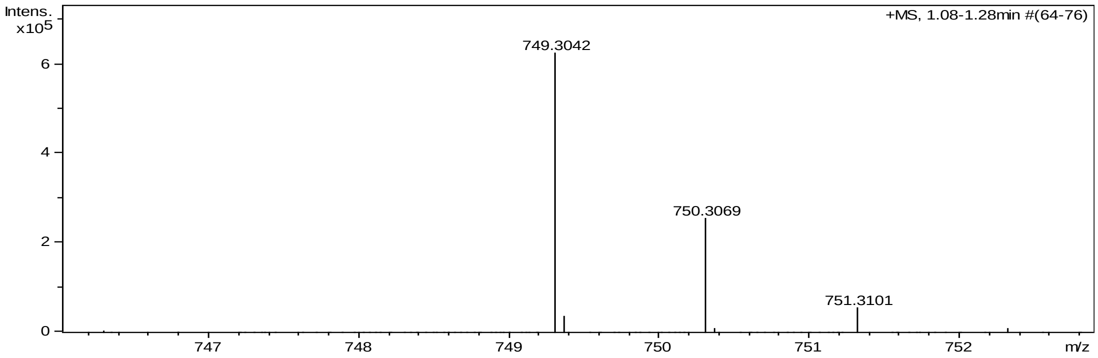
Intens.
x10^5
+MS, 1.08-1.28min #(64-76)
749.3042
750.3069
751.3101
m/z

4b,5,6,7-Tetraethyl 3-methyl *rel*-(4*R*,4a*R*,4b*S*,8a*S*)-1-benzyl-8-cyclohexyl-2-methyl-4-phenyl-4,4a,8,8a-tetrahydrocyclopenta[4,5]pyrrolo[2,3-*b*]pyridine-3,4b,5,6,7(1*H*)-pentacarboxylate (4p): yellow solid, 72%, m.p. 182-184 °C; ^{1}H NMR (400 MHz, $CDCl_3$) δ 7.49-7.38 (m, 5H, ArH), 7.04-6.59 (m, 3H, ArH), 6.72 (t, *J* = 6.8 Hz, 2H, ArH), 5.85 (d, *J* = 3.6 Hz, 1H, CH), 5.00-4.94 (m, 1H, CH),4.87 (d, *J* = 15.6 Hz, 1H, CH_2), 4.44-4.40 (m, 1H, CH_2), 4.37-4.33 (m, 1H, CH_2), 4.30 (d, *J* = 15.6 Hz, 1H, CH_2), 4.22-4.17 (m, 2H, CH_2), 4.14-4.08 (m, 1H, CH_2), 4.00-3.92 (m, 1H, CH_2), 3.65-3.57 (m, 1H, CH_2), 3.26 (s, 3H, OCH_3), 3.24-3.22 (m, 1H, CH), 3.19-3.10 (m, 1H, CH_2), 3.00-2.97 (m, 1H, CH), 2.34 (s, 3H, CH_3), 1.95-1.85 (m, 2H, CH_2), 1.79-1.71 (m, 4H, CH_2), 1.59-1.57 (m, 1H, CH_2), 1.48-1.45 (m, 1H, CH_2), 1.43-1.40 (m, 4H, CH_2, CH_3), 1.30 (t, *J* = 7.2 Hz, 3H, CH_3), 1.18-1.13 (m, 1H, CH_2), 1.09 (t, *J* = 7.2 Hz, 3H, CH_3), 0.72 (t, *J* = 7.2 Hz, 3H, CH_3) ppm. ^{13}C NMR (100 MHz, $CDCl_3$) δ 170.2, 167.9, 167.1, 165.8, 162.2, 161.5, 157.4, 145.8, 144.1, 137.3, 129.1, 128.9, 128.2, 127.5, 125.7, 113.6, 112.3, 99.5, 83.6, 72.4, 62.0, 61.0, 60.1, 59.4, 58.3, 56.6, 52.2, 50.4, 38.3, 31.9, 31.1, 26.2, 25.7, 25.5, 18.6, 14.1, 14.0, 13.8, 13.5 ppm. IR (KBr) υ: 3435, 2930, 2858, 1753, 1731, 1684, 1577, 1541, 1445, 1387, 1347, 1327, 1229, 1196, 1126, 1092, 1058, 1027, 984, 895, 757 cm^{-1}; MS (*m*/*z*): HRMS (ESI) Calcd. for $C_{44}H_{52}NaN_2O_{10}$ ([M+Na]$^+$): 791.3514, found: 791.3512.

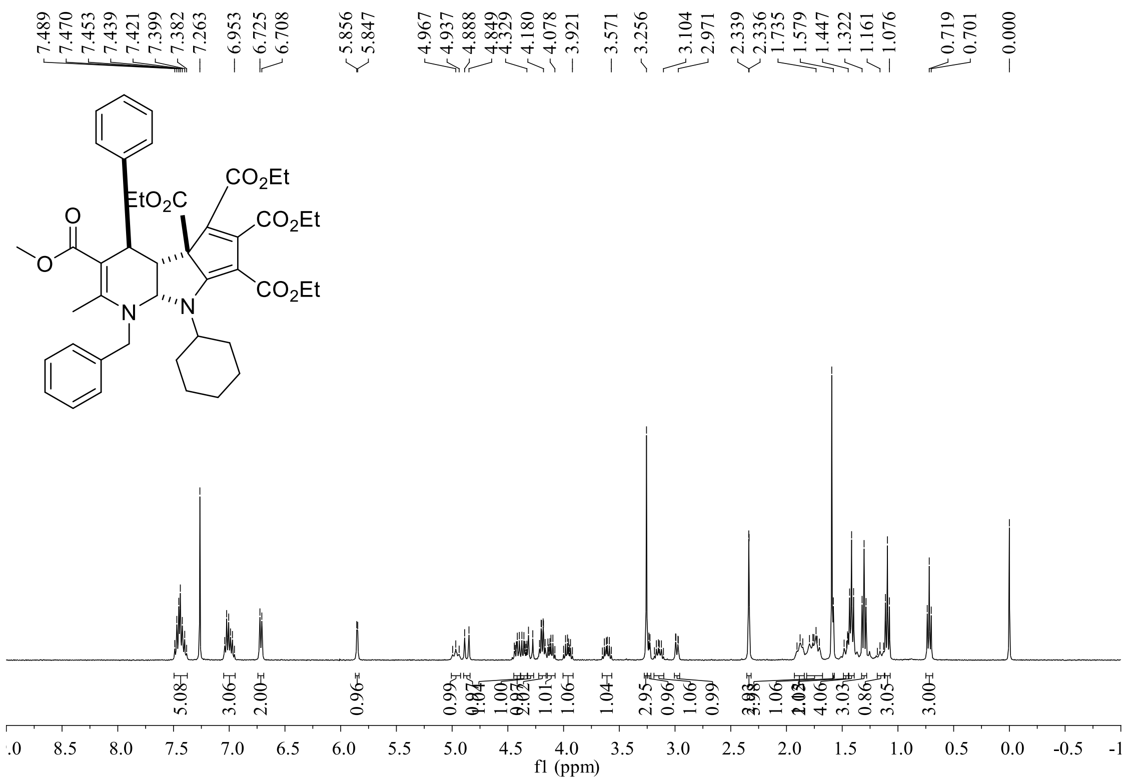

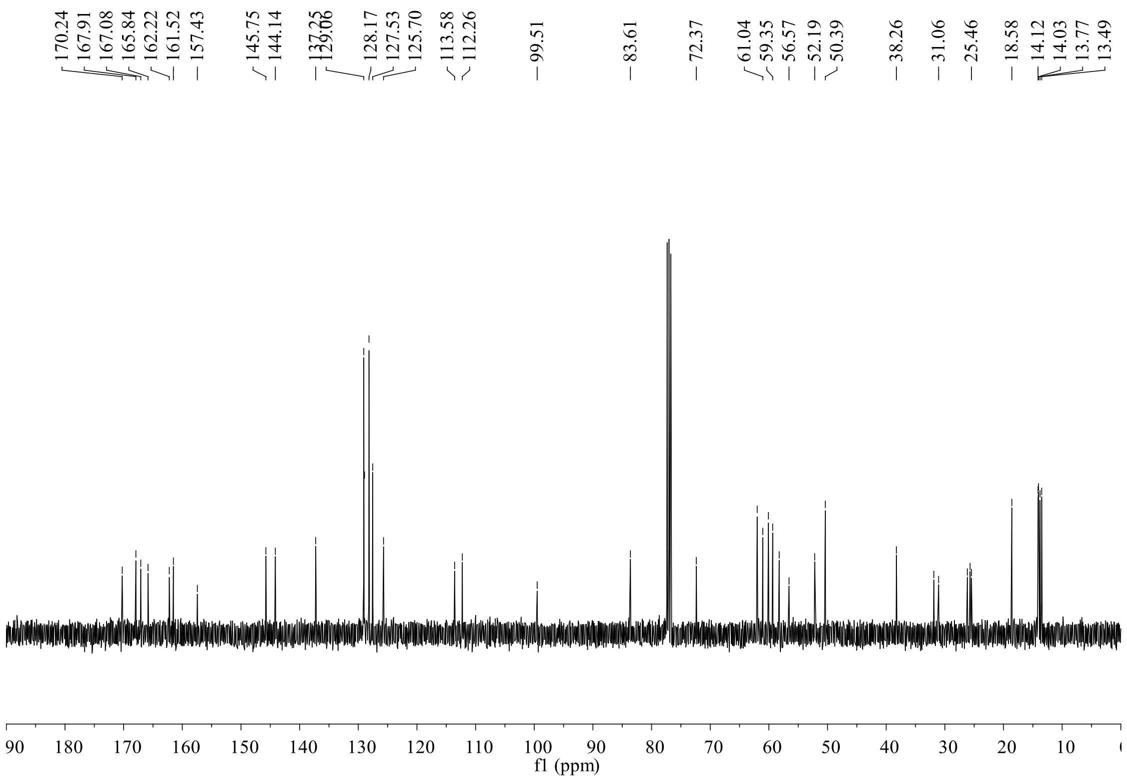
170.24
167.91
167.08
165.84
162.22
161.52
157.43
145.75
144.14
137.25
129.06
128.17
127.53
125.70
113.58
112.26
99.51
83.61
72.37
61.04
59.35
56.57
52.19
50.39
38.26
31.06
25.46
18.58
14.12
14.03
13.77
13.49
90 180 170 160 150 140 130 120 110 100 90 80 70 60 50 40 30 20 10
f1 (ppm)

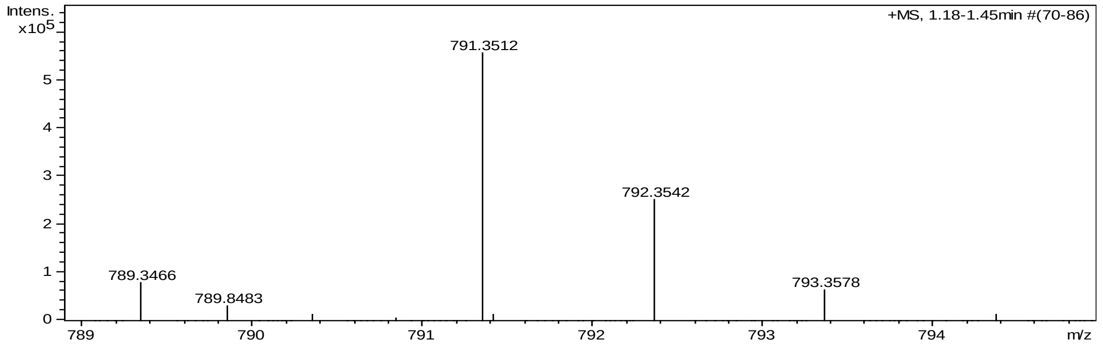
Intens.
x10^5
+MS, 1.18-1.45min #(70-86)
791.3512
792.3542
789.3466
789.8483
793.3578
0
1
2
3
4
5
789
790
791
792
793
794
m/z

Pentamethyl *rel*-(4*R*,4a*R*,4b*S*,8a*S*)-1-benzyl-8-(*tert*-butyl)-2-methyl-4-phenyl-4,4a,8,8a-tetrahydrocyclopenta[4,5]pyrrolo[2,3-*b*]pyridine-3,4b,5,6,7(1*H*)-pentacarboxylate (4q): yellow solid, 66%, m.p. 207-209 °C; ^{1}H NMR (400 MHz, $CDCl_3$) δ 7.35-7.28 (m, 3H, ArH), 7.16-7.10 (m, 2H, ArH), 7.00 (s, 3H, ArH), 6.57 (s, 2H, ArH) 5.85 (d, J = 4.4 Hz, 1H, CH), 4.75 (d, J = 14.8 Hz, 1H, CH_2), 4.34 (d, J = 14.8 Hz, 1H, CH_2), 3.94 (s, 3H, OCH_3), 3.72 (s, 3H, OCH_3), 3.63 (s, 3H, OCH_3), 3.54-3.50 (m, 1H, CH), 3.41 (s, 3H, OCH_3), 3.28 (s, 3H, OCH_3), 3.25-3.22 (m, 1H, CH), 2.40 (s, 3H, CH_3), 1.48 (s, 9H, CH_3) ppm. ^{13}C NMR (100 MHz, $CDCl_3$) δ 170.0, 168.9, 167.4, 166.2, 162.5, 161.9, 157.2, 150.3, 144.3, 137.0, 129.0, 128.8, 128.13, 128.07, 127.8, 125.6, 115.3, 114.4, 110.0, 104.3, 86.2, 74.0, 58.1, 55.5, 53.2, 52.4, 51.2, 51.1, 51.0, 50.7, 37.7, 29.6, 18.7 ppm. IR (KBr) υ: 3408, 2951, 2360, 1732, 1707, 1596, 1537, 1433, 1384, 1347, 1225, 1205, 1107, 1012, 1002, 967, 943, 793, 778 cm^{-1}; MS (*m*/*z*): HRMS (ESI) Calcd. for $C_{38}H_{42}NaN_2O_{10}$ ([M+Na]$^+$): 709.2732, found: 709.2737.

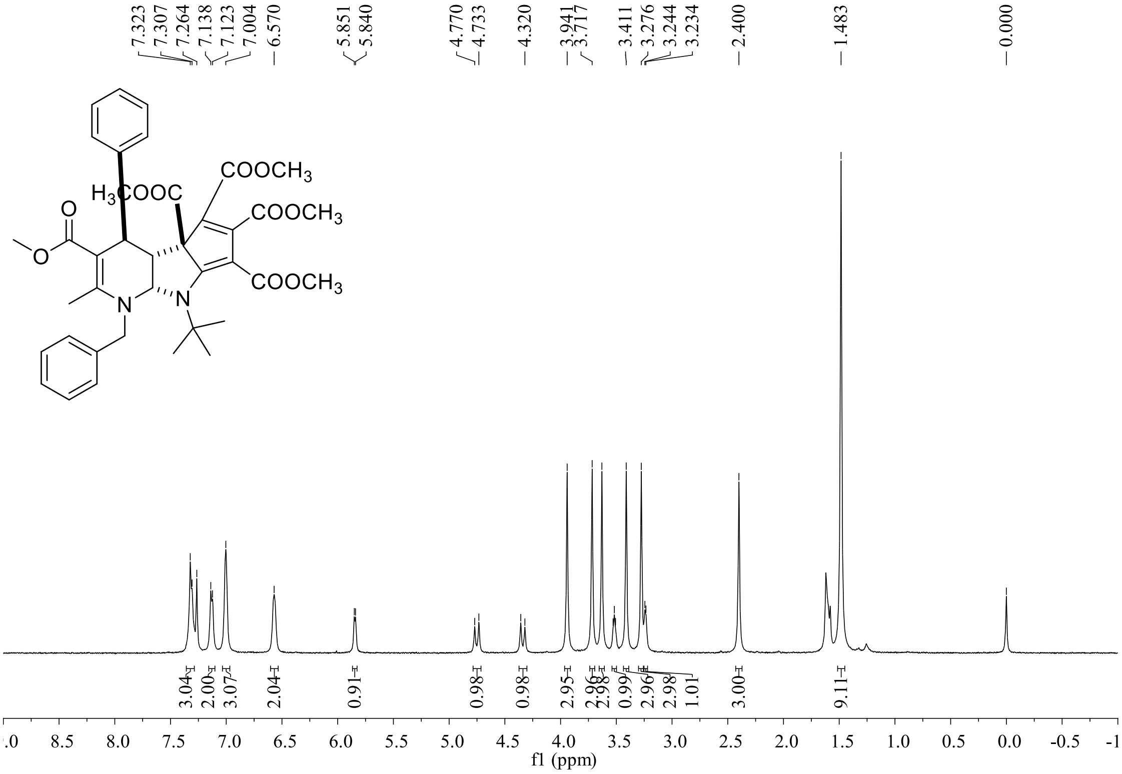

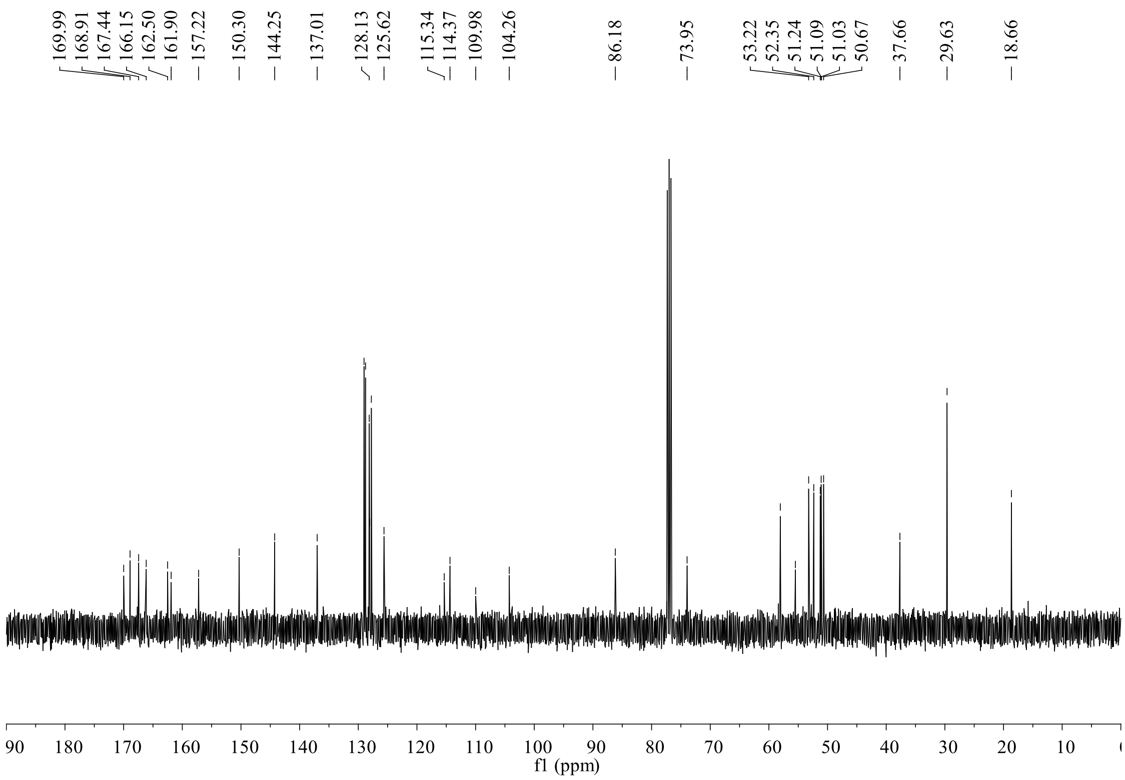
169.99
168.91
167.44
166.15
162.50
161.90
157.22
150.30
144.25
137.01
128.13
125.62
115.34
114.37
109.98
104.26
86.18
73.95
53.22
52.35
51.24
51.09
51.03
50.67
37.66
29.63
18.66
f1 (ppm)

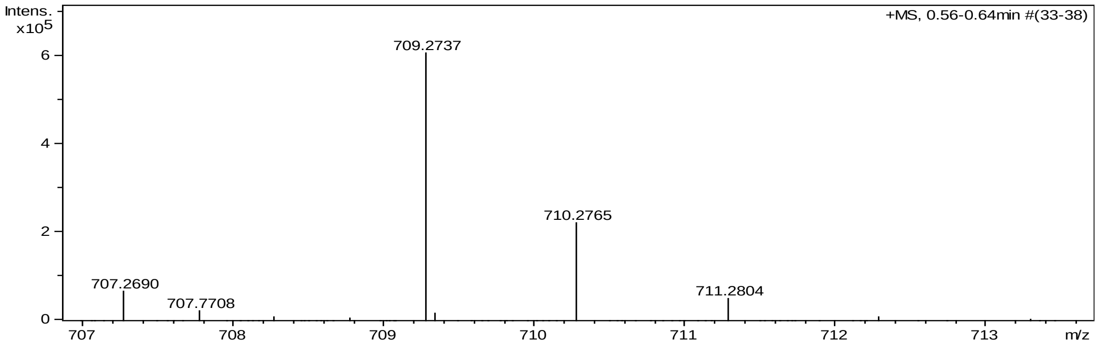
Intens.
x10^5
+MS, 0.56-0.64min #(33-38)
709.2737
710.2765
707.2690
707.7708
711.2804
m/z

4b,5,6,7-Tetraethyl 3-methyl *rel*-(4*R*,4a*R*,4b*S*,8a*S*)-1-benzyl-8-(*tert*-butyl)-2-methyl-4-phenyl-4,4a,8,8a-tetrahydrocyclopenta[4,5]pyrrolo[2,3-*b*]pyridine-3,4b,5,6,7(1*H*)-pentacarboxylate (4r): yellow solid, 58%, m.p. 141-143 °C; ^{1}H NMR (400 MHz, $CDCl_3$) δ 7.36-7.29 (m, 3H, ArH), 7.23-7.17 (m, 2H, ArH), 6.97 (s, 3H, ArH), 6.52 (d, *J* = 4.0 Hz, 2H, ArH), 5.88 (d, *J* = 3.6 Hz, 1H, CH), 4.76 (d, *J* = 14.8 Hz, 1H, CH_2),4.45-4.32 (m, 3H, CH_2, CH_2), 4.23-4.17 (m, 2H, CH_2), 4.11-4.09 (m, 2H, CH_2), 3.87-3.79 (m, 1H, CH_2), 3.54-3.48 (m, 1H, CH_2), 3.46-3.43 (m, 1H, CH), 3.39 (s, 3H, OCH_3), 3.21-3.16 (m, 1H, CH), 2.40 (s, 3H, CH_3), 1.50 (s, 9H, CH_3), 1.41 (t, *J* = 6.8 Hz, 3H, CH_3), 1.28 (t, *J* = 6.4 Hz, 3H, CH_3), 1.18 (t, *J* = 6.4 Hz, 3H, CH_3), 0.84 (t, *J* = 6.8 Hz, 3H, CH_3) ppm. ^{13}C NMR (100 MHz, $CDCl_3$) δ 170.3, 168.3, 167.5, 165.7, 162.2, 161.5, 157.0, 149.6, 144.5, 137.2, 129.0, 128.9, 128.3, 128.1, 127.6, 125.5, 116.0, 114.6, 105.0, 85.9, 73.9, 62.0, 61.1, 60.1, 59.8, 57.9, 55.5, 50.9, 50.5, 37.7, 29.7, 18.7, 14.11, 14.07, 13.6 ppm. IR (KBr) υ: 3399, 2980, 2360, 1731, 1701, 1591, 1534, 1455, 1368, 1347, 1330, 1226, 1193, 1111, 1029, 1004, 967, 857, 792, 763 cm^{-1}; MS (*m*/*z*): HRMS (ESI) Calcd. for $C_{42}H_{50}NaN_2O_{10}$ ($[M+Na]^+$): 765.3358, found: 765.3354.

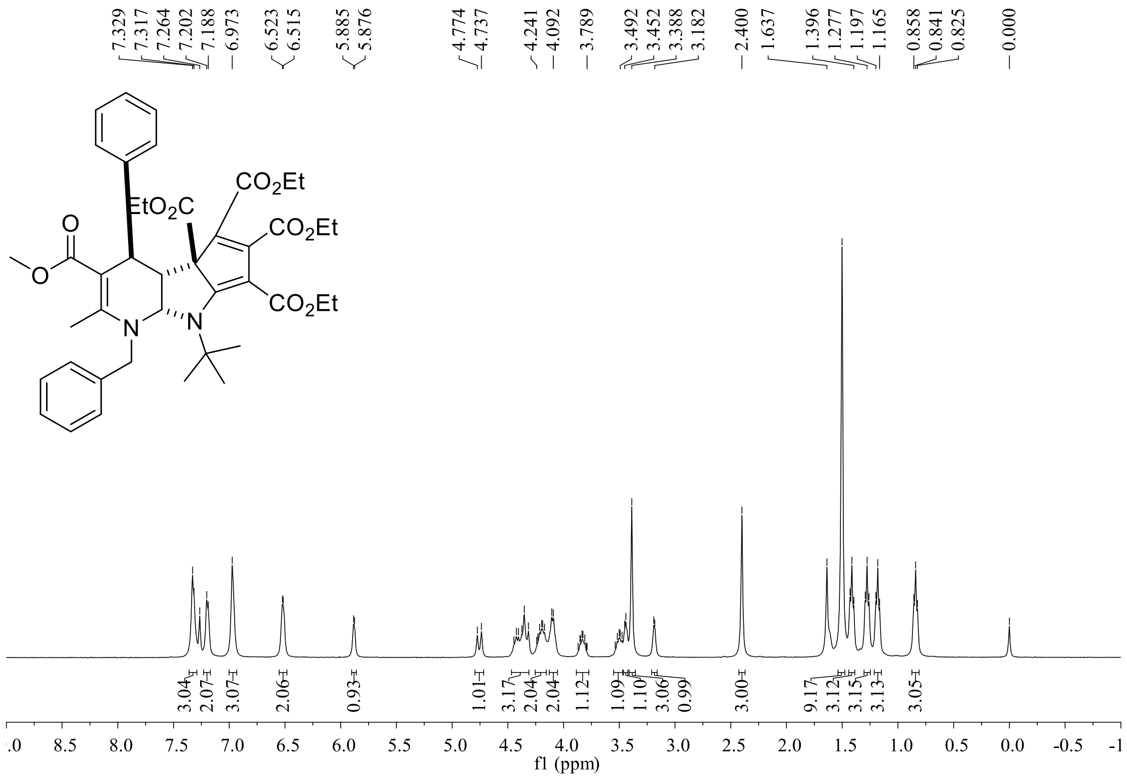

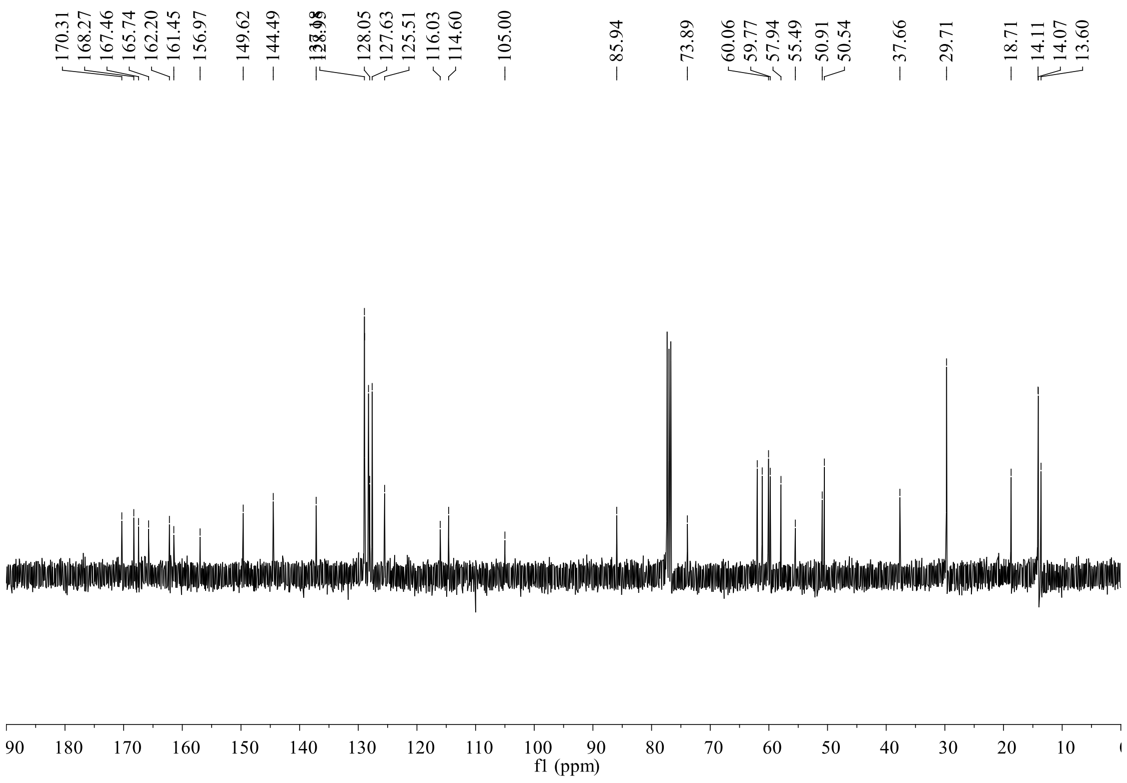
170.31
168.27
167.46
165.74
162.20
161.45
156.97
149.62
144.49
128.05
127.63
125.51
116.03
114.60
105.00
85.94
73.89
60.06
59.77
57.94
55.49
50.91
50.54
37.66
29.71
18.71
14.11
14.07
13.60
f1 (ppm)

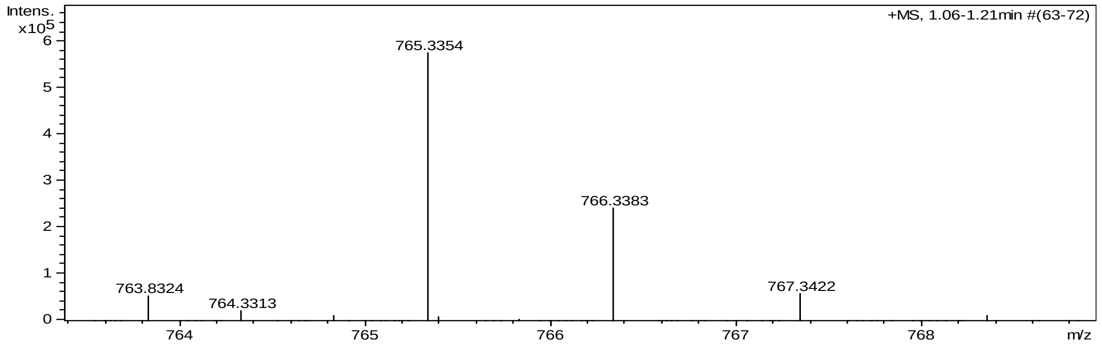
Intens.
x10^5
+MS, 1.06-1.21min #(63-72)
765.3354
766.3383
763.8324
764.3313
767.3422
m/z

Pentamethyl *rel*-(4*R*,4a*R*,4b*S*,8a*S*)-1,8-dibenzyl-2-methyl-4-phenyl-4,4a,8,8a-tetrahydrocyclopenta[4,5]pyrrolo[2,3-*b*]pyridine-3,4b,5,6,7(1*H*)-pentacarboxylate (4s): yellow solid, 54%, m.p. 235-237 °C; ^{1}H NMR (400 MHz, $CDCl_3$) δ 7.40 (t, *J* = 7.2 Hz, 2H, ArH), 7.34 (t, *J* = 6.0 Hz, 2H, ArH), 7.32-7.27 (m, 2H, ArH), 7.18 (t, *J* = 7.2 Hz, 4H, ArH), 7.15-7.10 (m, 2H, ArH), 7.07-7.02 (m, 1H, ArH), 6.95 (d, *J* = 7.2 Hz, 2H, ArH), 5.73 (d, *J* = 15.6 Hz, 1H, CH_2), 5.59-5.55 (m, 1H, CH),4.75 (d, *J* = 15.6 Hz, 1H, CH_2), 4.71 (d, *J* = 16.0 Hz, 1H, CH_2), 3.99 (s, 3H, OCH_3), 3.86 (d, *J* = 16.0 Hz, 1H, CH_2), 3.67 (s, 3H, OCH_3), 3.59 (s, 3H, OCH_3), 3.57-3.54 (m, 1H, CH), 3.28 (s, 3H, OCH_3), 3.15 (d, *J* = 8.4 Hz, 1H, CH), 3.02 (s, 3H, OCH_3), 2.06 (s, 3H, CH_3) ppm. ^{13}C NMR (100 MHz, $CDCl_3$) δ 170.7, 168.1, 167.2, 166.2, 162.7, 162.0, 157.2, 145.5, 143.9, 137.4, 137.3, 129.1, 128.9, 128.8, 128.0, 127.9, 127.7, 126.9, 126.6, 126.0, 113.2, 109.3, 98.9, 83.9, 72.7, 55.7, 53.4, 52.8, 52.5, 51.4, 50.8, 50.5, 49.0, 38.3, 17.1 ppm. IR (KBr) υ: 3391, 2949, 1740, 1692, 1590, 1556, 1438, 1381, 1342, 1226, 1116, 1096, 1073, 1015, 992, 840, 815, 799, 788, 760 cm^{-1}; MS (*m*/*z*): HRMS (ESI) Calcd. for $C_{41}H_{40}NaN_2O_{10}$ ([M+Na]$^{+}$): 743.2575, found: 743.2579.

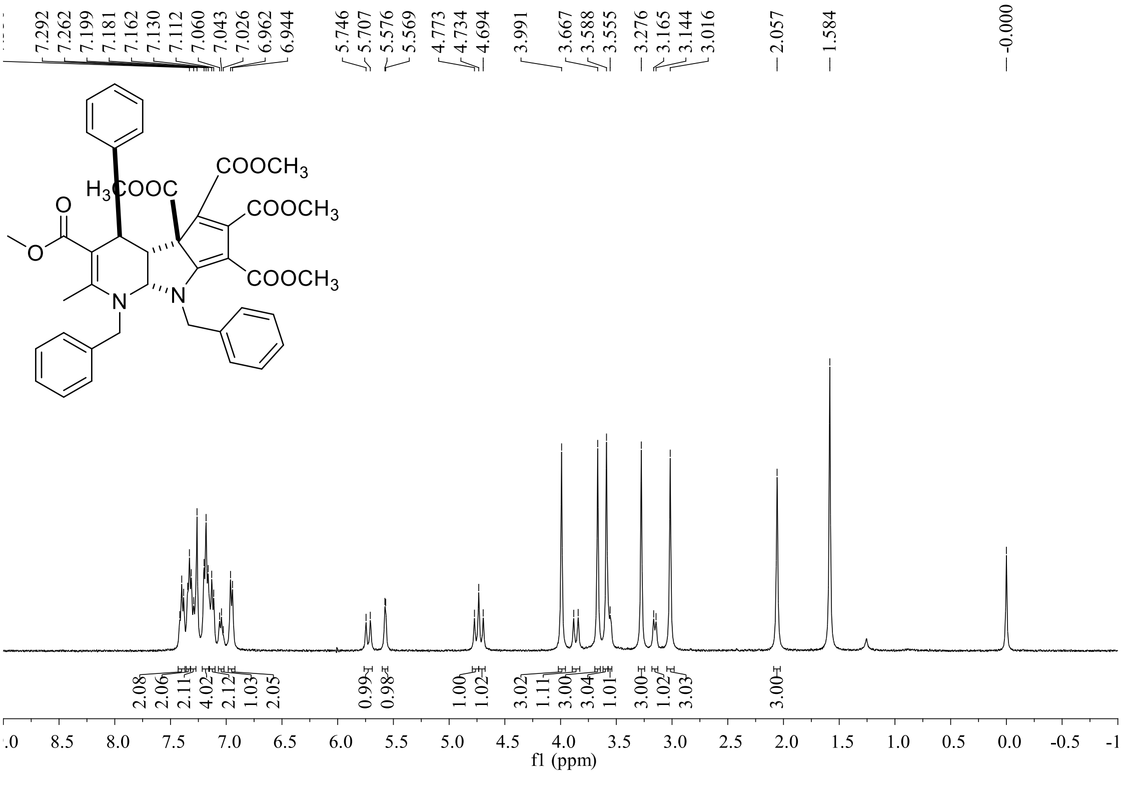

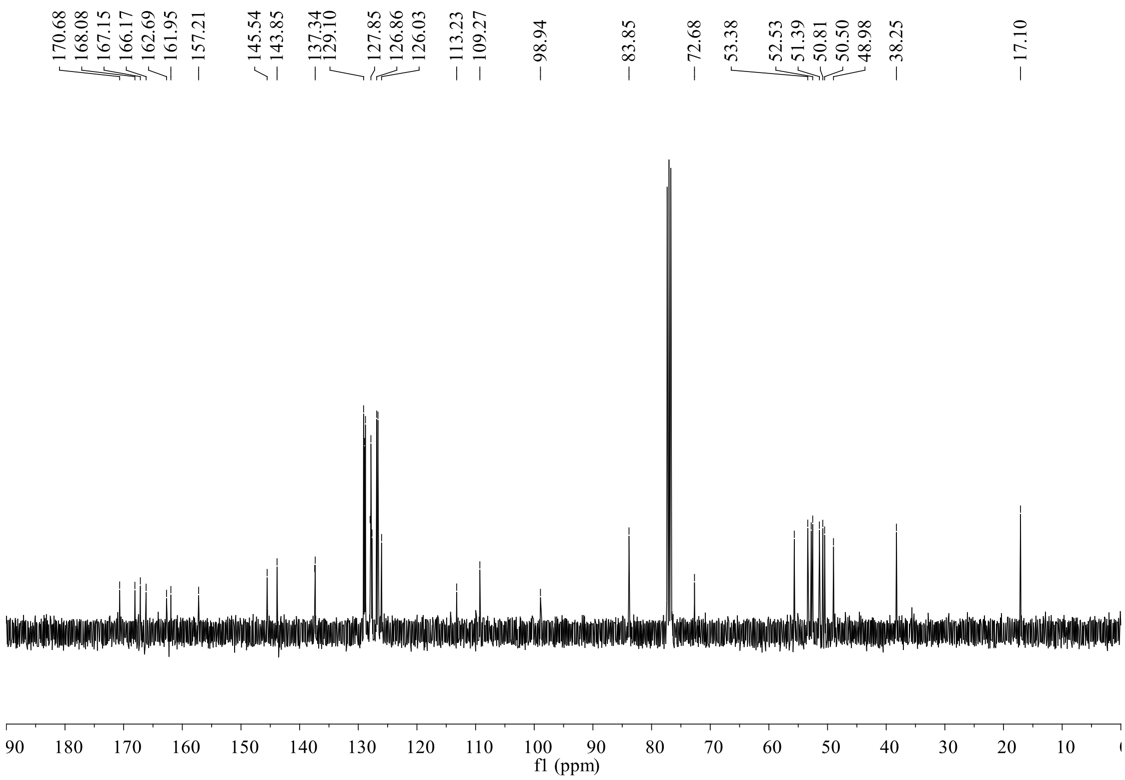
170.68
168.08
167.15
166.17
162.69
161.95
157.21
145.54
143.85
137.34
129.10
127.85
126.86
126.03
113.23
109.27
98.94
83.85
72.68
53.38
52.53
51.39
50.81
50.50
48.98
38.25
17.10
f1 (ppm)

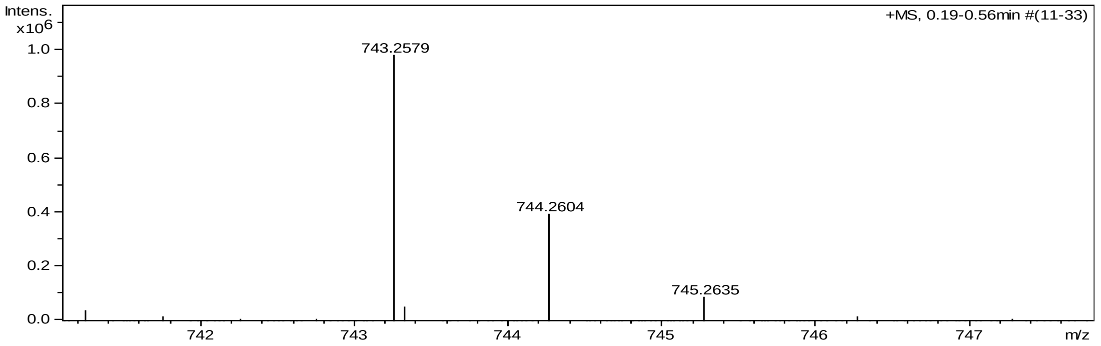
Intens.
x10^6
+MS, 0.19-0.56min #(11-33)
743.2579
744.2604
745.2635
m/z

4b,5,6,7-Tetraethyl 3-methyl *rel*-(4*R*,4a*R*,4b*S*,8a*S*)-1,8-dibenzyl-2-methyl-4-phenyl-4,4a,8,8a-tetrahydrocyclopenta[4,5]pyrrolo[2,3-*b*]pyridine-3,4b,5,6,7(1*H*)-pentacarboxylate (4t): yellow solid, 30%, m.p. 170-172 °C; ^{1}H NMR (400 MHz, $CDCl_3$) δ 7.38 (t, *J* = 7.2 Hz, 2H, ArH), 7.35-7.27 (m, 4H, ArH), 7.22-7.15 (m, 4H, ArH), 7.11 (t, *J* = 7.2 Hz, 2H, ArH), 7.03 (t, *J* = 7.2 Hz, 1H, ArH), 6.95 (d, *J* = 7.2 Hz, 2H, ArH), 5.75 (d, *J* = 15.6 Hz, 1H, CH_2), 5.62 (d, *J* = 3.6 Hz, 1H, CH), 4.74 (d, *J* = 15.6 Hz, 1H, CH_2), 4.68 (d, *J* = 16.4 Hz, 1H, CH_2), 4.74-4.38 (m, 2H, CH_2), 4.17-4.13 (m, 2H, CH_2), 4.12-4.07 (m, 1H, CH_2), 4.02-3.94 (m, 1H, CH_2), 3.83 (d, *J* = 16.0 Hz, 1H, CH_2), 3.73-3.65 (m, 1H, CH_2), 3.53(dd, J_1 = 8.8 Hz, J_2 = 4.0 Hz, 1H, CH), 3.27 (s, 3H, OCH_3), 3.25-3.17 (m, 2H, CH_2,CH), 2.06 (s, 3H, CH_3), 1.44 (t, *J* = 7.2 Hz, 3H, CH_3), 1.18 (t, *J* = 7.2 Hz, 3H, CH_3), 1.11 (t, *J* = 7.2 Hz, 3H, CH_3), 0.79 (t, *J* = 7.2 Hz, 3H, CH_3) ppm. ^{13}C NMR (100 MHz, $CDCl_3$) δ 170.5, 168.2, 166.7, 165.7, 162.6, 161.6, 157.1, 145.8, 144.2, 137.5, 129.0, 128.9, 128.7, 127.9, 127.8, 127.5, 126.9, 126.7, 125.9, 113.9, 110.0, 109.5, 99.1, 83.8, 73.0, 62.2, 61.2, 60.2, 59.5, 55.6, 52.9, 50.5, 49.0, 38.3, 17.1, 14.1, 13.9, 13.8, 13.7 ppm. IR (KBr) υ: 3390, 2944, 2872, 1729, 1692, 1593, 1563, 1431, 1384, 1334, 1228, 1194, 1100, 1081, 1030, 970, 880, 866, 841, 813, 770, 759 cm^{-1}; MS (*m/z*): HRMS (ESI) Calcd. for $C_{45}H_{48}NaN_2O_{10}$ ([M+Na]$^+$): 799.3201, found: 799.3203.

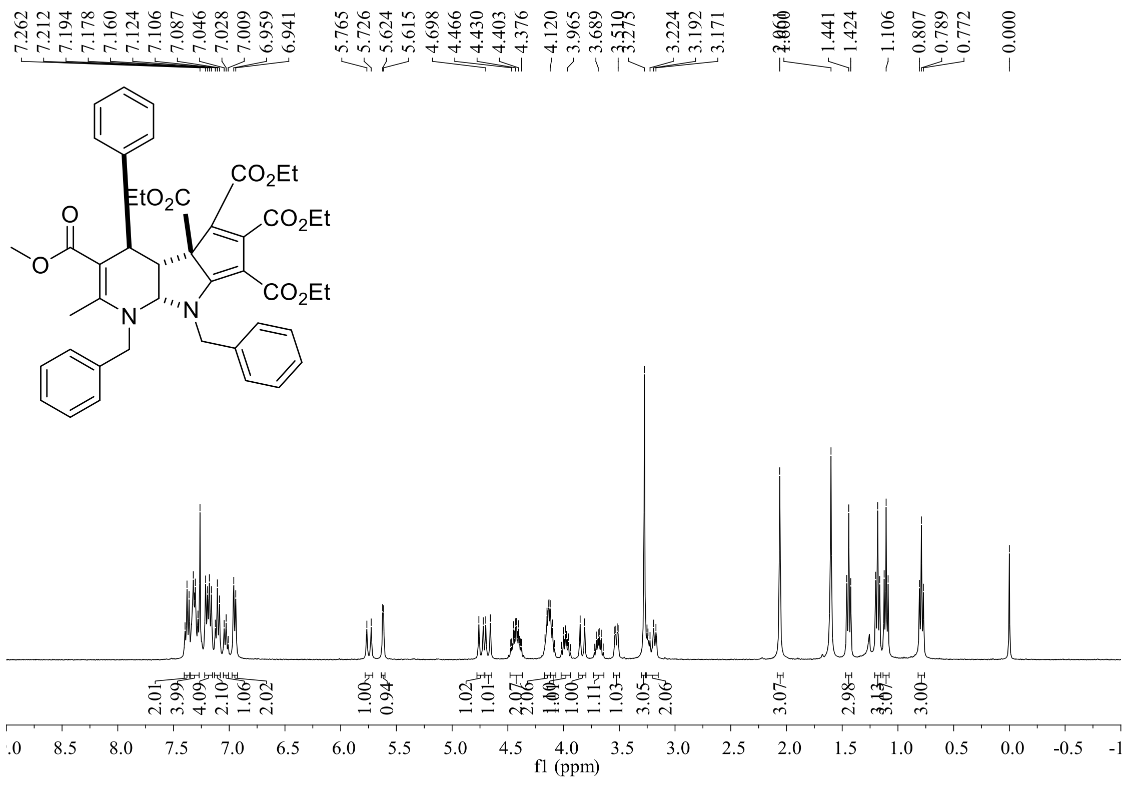

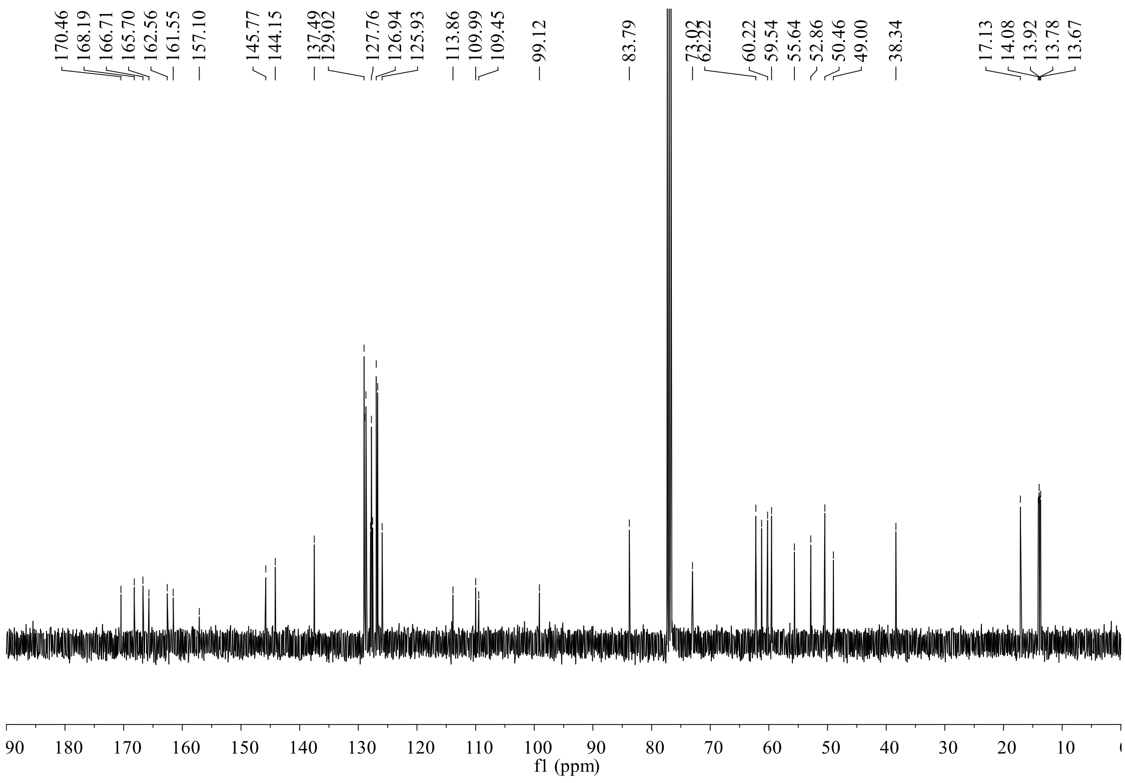
170.46
168.19
166.71
165.70
162.56
161.55
157.10
145.77
144.15
137.49
129.02
127.76
126.94
125.93
113.86
109.99
109.45
99.12
83.79
73.02
62.22
60.22
59.54
55.64
52.86
50.46
49.00
38.34
17.13
14.08
13.92
13.78
13.67
f1 (ppm)

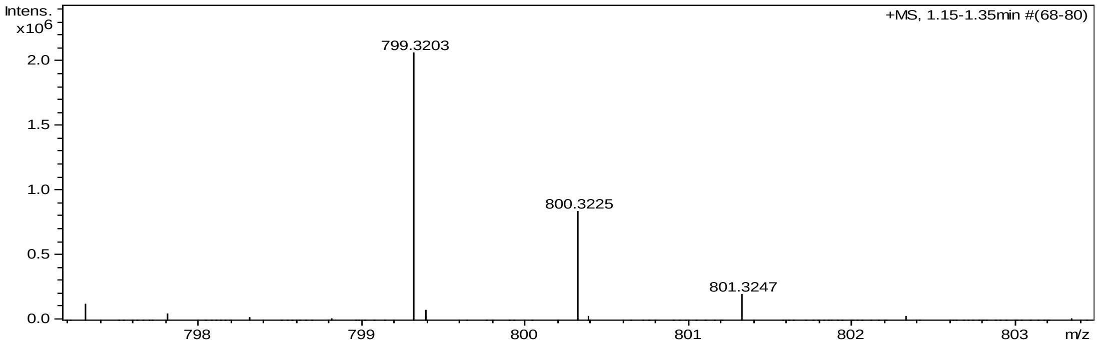
Intens.
x10^6
+MS, 1.15-1.35min #(68-80)
799.3203
800.3225
801.3247
m/z

Pentamethyl ***rel*-(4*R*,4a*R*,4b*S*,8a*S*)-1-benzyl-8-cyclohexyl-4-phenyl-4,4a,8,8a-tetrahydrocyclopenta[4,5]pyrrolo[2,3-*b*]pyridine-3,4b,5,6,7(1*H*)-pentacarboxylate (6a)**: yellow solid, 92%, m.p. 210-212 °C; ^{1}H NMR (400 MHz, $CDCl_3$) δ 7.53-7.49 (m, 2H, CH, ArH), 7.45-7.39 (m, 4H, ArH), 7.09 (t, J = 7.2 Hz, 2H, ArH), 7.02 (t, J = 7.2 Hz, 1H, ArH), 6.87 (d, J = 7.2 Hz, 2H, ArH), 5.76 (d, J = 3.2 Hz, 1H, CH), 4.94-4.86 (m, 1H, CH),4.58-4.50 (m, 2H, CH_2), 3.95 (s, 3H, OCH_3), 3.73 (s, 3H, OCH_3), 3.56 (s, 3H, OCH_3), 3.36 (s, 3H, OCH_3), 3.26 (dd, J_1 = 8.4 Hz, J_2 = 3.2 Hz, 1H, CH), 2.95 (s, 3H, OCH_3), 2.84 (d, J = 8.4 Hz, 1H, CH), 1.94-1.87 (m, 2H, CH_2), 1.81-1.72 (m, 4H, CH_2), 1.64-1.55 (m, 1H, CH_2), 1.46-1.32 (m, 2H, CH_2), 1.22-1.16 (m, 1H, CH_2) ppm. ^{13}C NMR (100 MHz, $CDCl_3$) δ 170.5, 167.5, 166.7, 166.2, 162.3, 161.8, 157.3, 143.8, 141.7, 136.8, 129.3, 128.9, 128.5, 127.8, 127.7, 125.9, 113.6, 108.9, 100.1, 81.2, 71.9, 60.0, 58.8, 53.4, 52.8, 52.4, 51.4, 50.8, 50.6, 36.0, 32.1, 31.4, 26.1, 25.7, 25.5 ppm. IR (KBr) υ: 3435, 2949, 2859, 1740, 1702, 1636, 1585, 1542, 1435, 1345, 1229, 1206, 1111, 1083, 1002, 980, 896, 852, 833, 791, 757 cm^{-1}; MS (*m*/*z*): HRMS (ESI) Calcd. for $C_{39}H_{42}NaN_2O_{10}$ ($[M+Na]^+$): 721.2732, found: 721.2725.

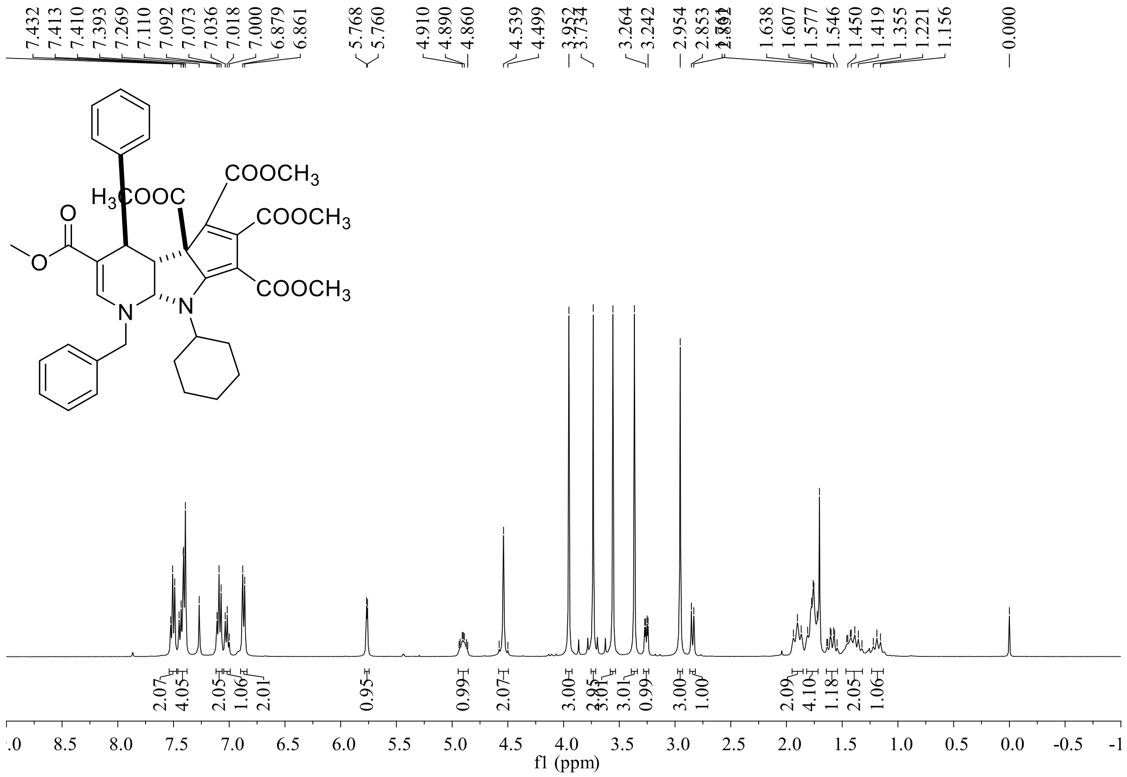

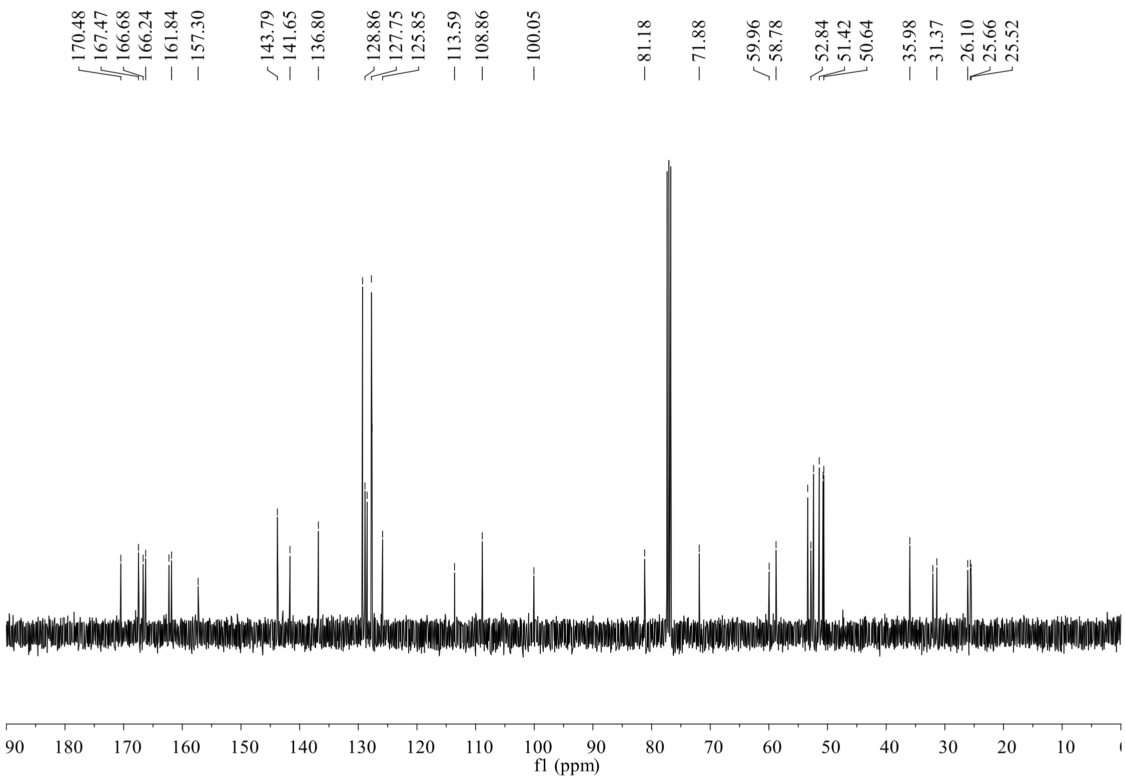

170.48
167.47
166.68
166.24
161.84
157.30
143.79
141.65
136.80
128.86
127.75
125.85
113.59
108.86
100.05
81.18
71.88
59.96
58.78
52.84
51.42
50.64
35.98
31.37
26.10
25.66
25.52
f1 (ppm)

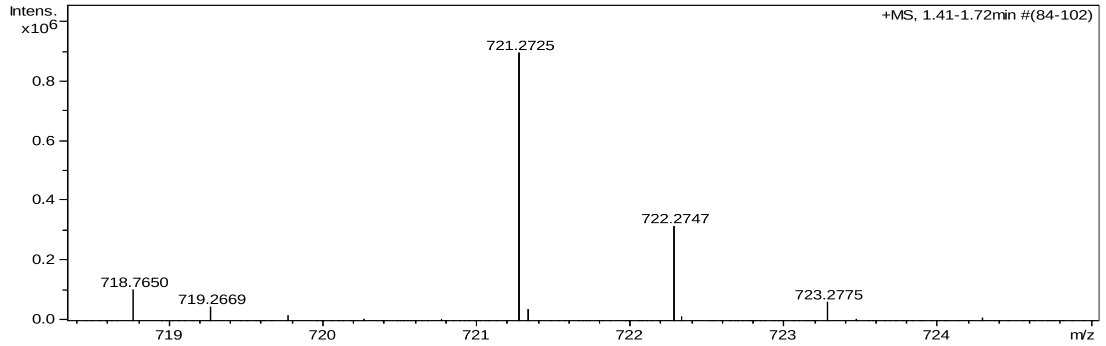

Intens.
x10^6
+MS, 1.41-1.72min #(84-102)
721.2725
722.2747
718.7650
719.2669
723.2775
m/z

Pentamethyl *rel*-(4*R*,4a*R*,4b*S*,8a*S*)-8-cyclohexyl-1-(3-methoxybenzyl)-4-phenyl-4,4a,8,8a-tetrahydrocyclopenta[4,5]pyrrolo[2,3-*b*]pyridine-3,4b,5,6,7(1*H*)-pentacarboxylate (6b): yellow solid, 82%, m.p. 197-199 °C; ^{1}H NMR (400 MHz, $CDCl_3$) δ 7.44-7.38 (m, 2H, CH, ArH), 7.12-7.03 (m, 3H, ArH), 6.99-6.90 (m, 5H, ArH), 5.76 (s, 1H, CH), 4.92-4.87 (m, 1H, CH), 4.55-4.46 (m, 2H, CH_2), 3.95 (s, 3H, OCH_3), 3.89 (s, 3H, OCH_3), 3.73 (s, 3H, OCH_3), 3.56 (s, 3H, OCH_3), 3.37 (s, 3H, OCH_3), 3.30 (dd, J_1 = 8.4 Hz, J_2 = 2.4 Hz, 1H, CH), 2.96 (s, 3H, OCH_3), 2.84 (d, J =8.4 Hz, 1H, CH), 1.93-1.86 (m, 2H, CH_2), 1.81-1.72 (m, 4H, CH_2), 1.59-1.53 (m, 1H, CH_2), 1.45-1.32 (m, 2H, CH_2), 1.21-1.12 (m, 1H, CH_2) ppm. ^{13}C NMR (100 MHz, $CDCl_3$) δ 170.5, 167.4, 166.6, 166.2, 162.3, 161.8, 160.2, 157.3, 143.9, 141.6, 138.4, 130.4, 128.9, 127.8, 125.9, 119.9, 114.0, 113.6, 113.3, 108.9, 100.1, 81.2, 71.9, 59.9, 58.8, 55.3, 53.4, 52.9, 52.4, 51.4, 50.7, 50.6, 36.0, 32.1, 31.4, 26.1, 25.7, 25.5 ppm. IR (KBr) υ: 3434, 2949, 2858, 1734, 1694, 1586, 1546, 1434, 1391, 1340, 1228, 1203, 1110, 1083, 1013, 981, 895, 852, 791 cm^{-1}; MS (*m*/*z*): HRMS (ESI) Calcd. for $C_{40}H_{44}NaN_2O_{11}$ ([M+Na]$^+$): 751.2837, found: 751.2830.

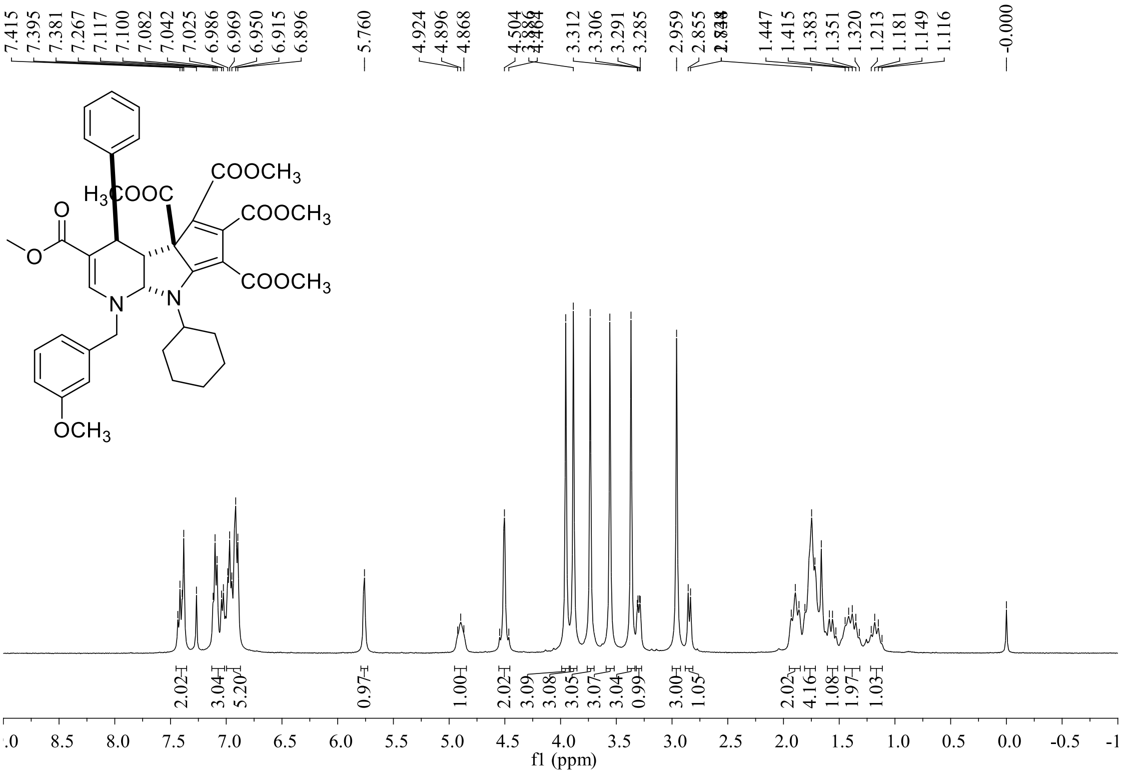

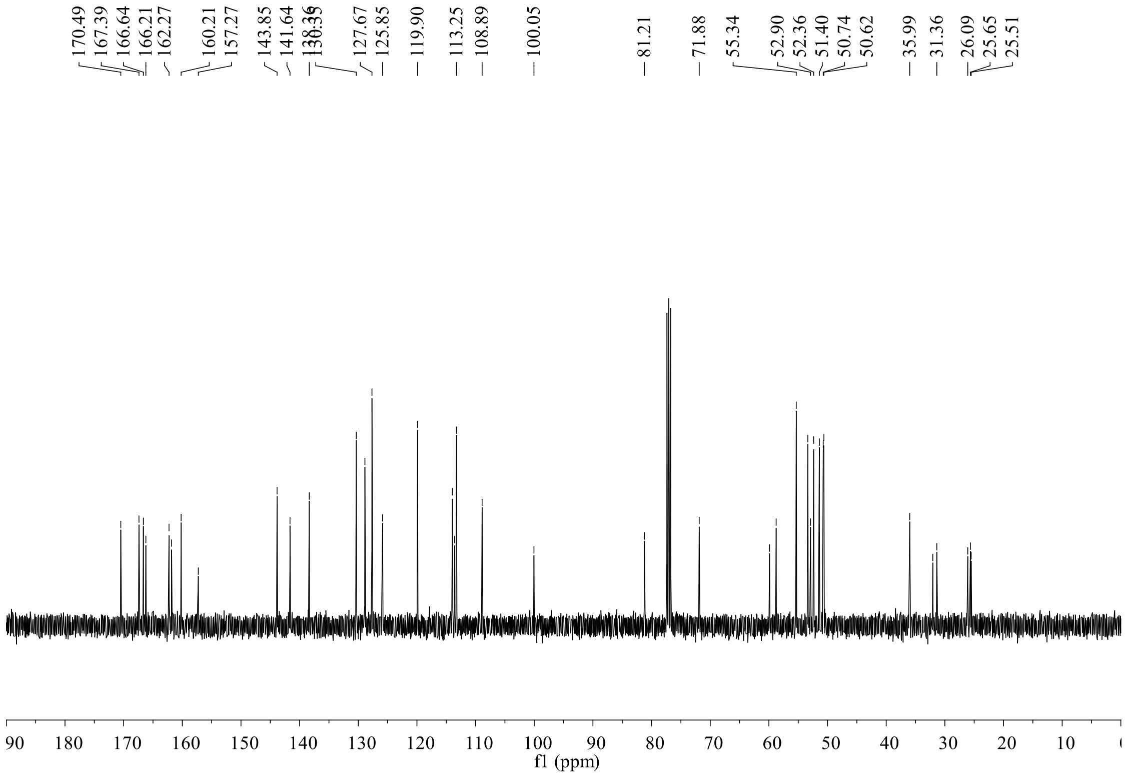
170.49
167.39
166.64
166.21
162.27
160.21
157.27
143.85
141.64
127.67
125.85
119.90
113.25
108.89
100.05
81.21
71.88
55.34
52.90
52.36
51.40
50.74
50.62
35.99
31.36
26.09
25.65
25.51
180 170 160 150 140 130 120 110 100 90 80 70 60 50 40 30 20 10
f1 (ppm)

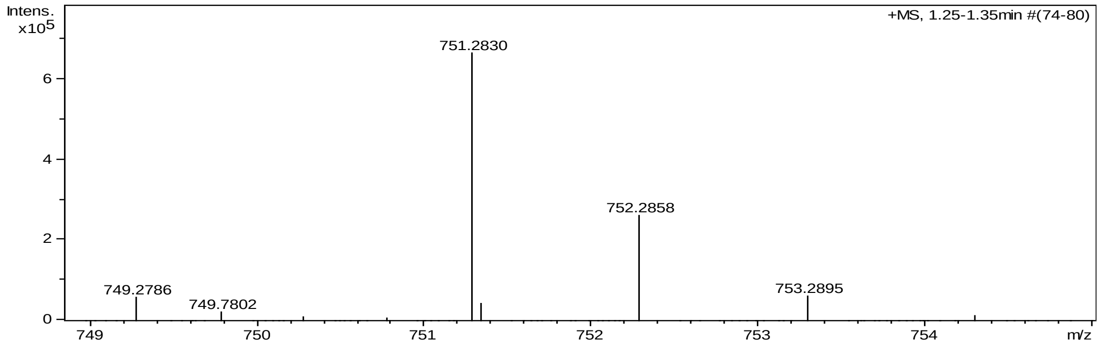
Intens.
x10^5
+MS, 1.25-1.35min #(74-80)
751.2830
752.2858
749.2786
749.7802
753.2895
749 750 751 752 753 754
m/z

Pentamethyl *rel*-(4*R*,4a*R*,4b*S*,8a*S*)-8-cyclohexyl-1-(2-methylbenzyl)-4-phenyl-4,4a,8,8a-tetrahydrocyclopenta[4,5]pyrrolo[2,3-*b*]pyridine-3,4b,5,6,7(1*H*)-pentacarboxylate (6c): yellow solid, 83%, m.p. 214-216 °C; ^{1}H NMR (400 MHz, $CDCl_3$) δ 7.39-7.29 (m, 5H, CH, ArH), 7.14 (t, *J* = 7.2 Hz, 2H, ArH), 7.07-6.98 (m, 3H, ArH), 5.70 (d, *J* = 2.8 Hz, 1H, CH), 4.84 (d, *J* = 11.6 Hz, 1H, CH), 4.64 (d, *J* = 5.2 Hz, 1H, CH_2), 4.57 (d, *J* = 5.2 Hz, 1H, CH_2), 3.96 (s, 3H, OCH_3), 3.74 (s, 3H, OCH_3), 3.55 (s, 3H, OCH_3), 3.45 (dd, J_1 = 8.4 Hz, J_2 = 2.8 Hz, 1H, CH), 3.35 (s, 3H, OCH_3), 2.98 (s, 3H, OCH_3), 2.89 (d, *J* = 8.8 Hz, 1H, CH), 2.47 (s, 3H, CH_3), 1.93-1.82 (m, 3H, CH_2), 1.81-1.70 (m, 3H, CH_2), 1.67-1.61 (m, 1H, CH_2), 1.44-1.32 (m, 2H, CH_2), 1.23-1.13 (m, 1H, CH_2) ppm. ^{13}C NMR (100 MHz, $CDCl_3$) δ 170.7, 167.4, 166.7, 166.3, 162.3, 161.8, 157.3, 143.9, 142.0, 135.8, 134.7, 131.2, 128.9, 128.5, 128.3, 127.8, 126.7, 125.9, 107.8, 100.3, 81.3, 77.3, 77.0, 76.7, 71.8, 58.7, 57.3, 53.4, 52.8, 52.4, 51.4, 50.8, 50.6, 36.2, 31.8, 31.4, 26.1, 25.8, 25.5, 19.5 ppm. IR (KBr) υ: 3434, 2948, 2850, 1740, 1701, 1583, 1542, 1434, 1344, 1304, 1227, 1205, 1109, 1081, 1001, 979, 896, 833, 790 cm^{-1}; MS (*m/z*): HRMS (ESI) Calcd. for $C_{40}H_{44}NaN_2O_{10}$ ($[M+Na]^+$): 735.2888, found: 735.2882.

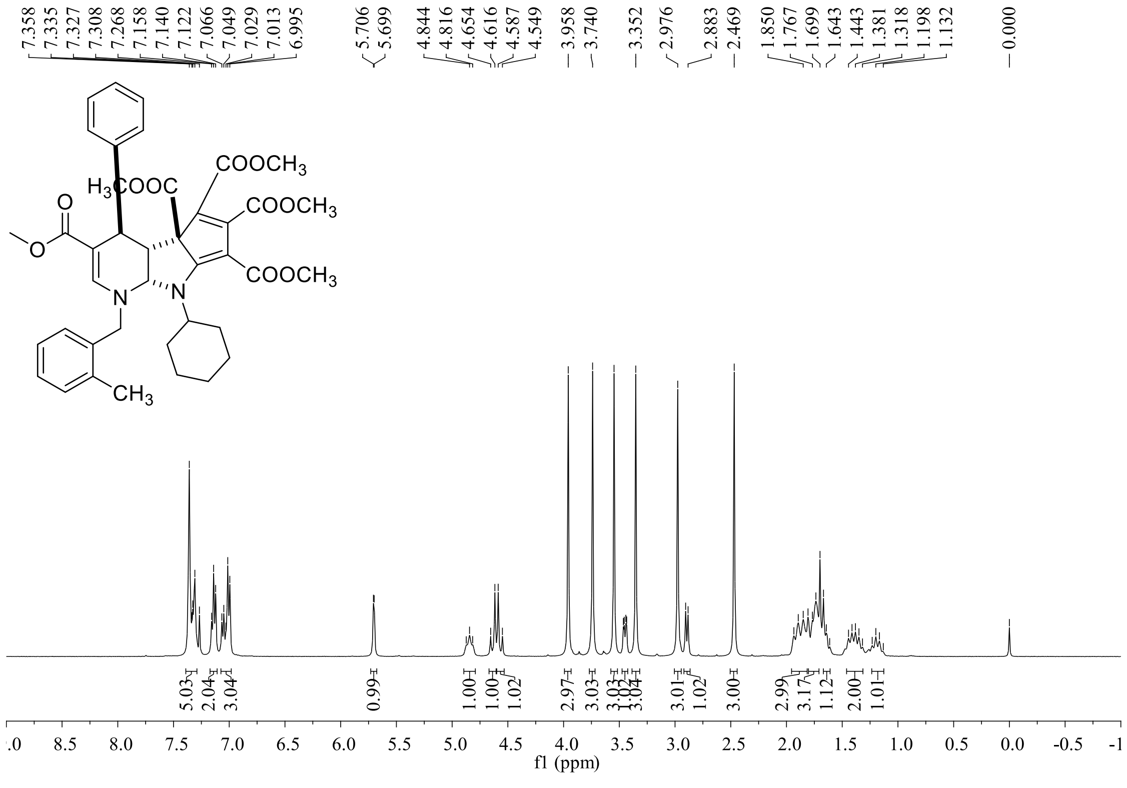

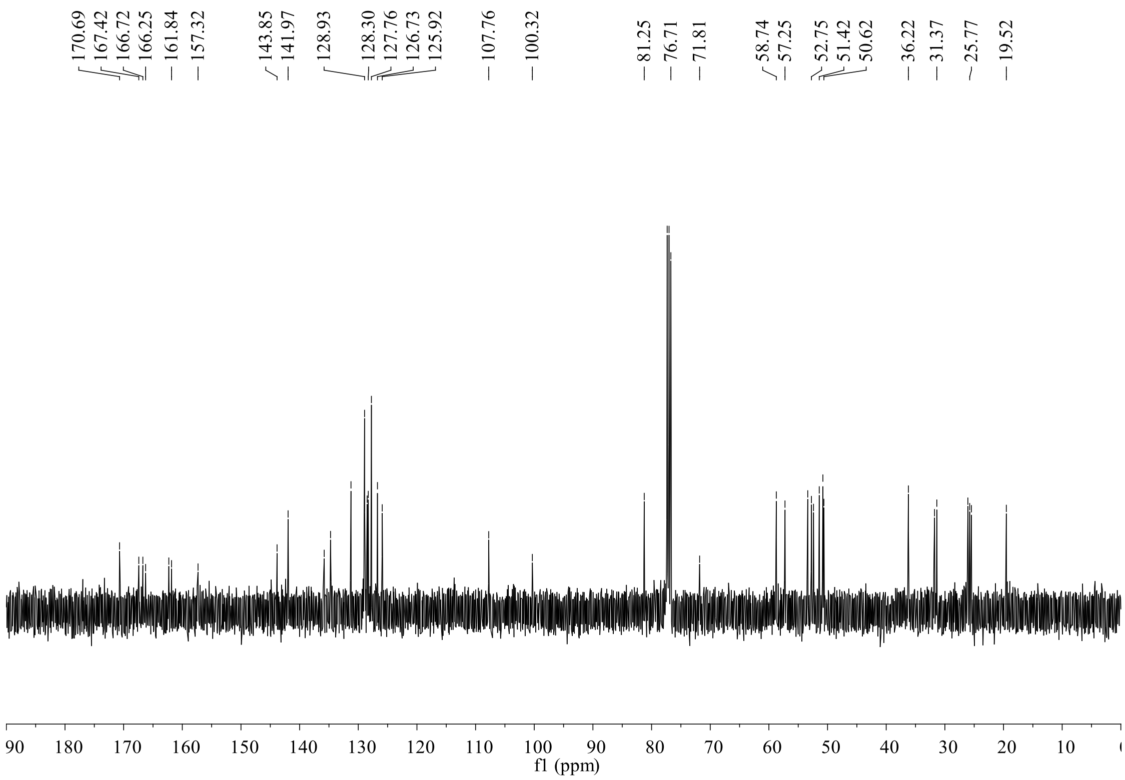
170.69
167.42
166.72
166.25
161.84
157.32
143.85
141.97
128.93
128.30
127.76
126.73
125.92
107.76
100.32
81.25
76.71
71.81
58.74
57.25
52.75
51.42
50.62
36.22
31.37
25.77
19.52
90 180 170 160 150 140 130 120 110 100 90 80 70 60 50 40 30 20 10
f1 (ppm)

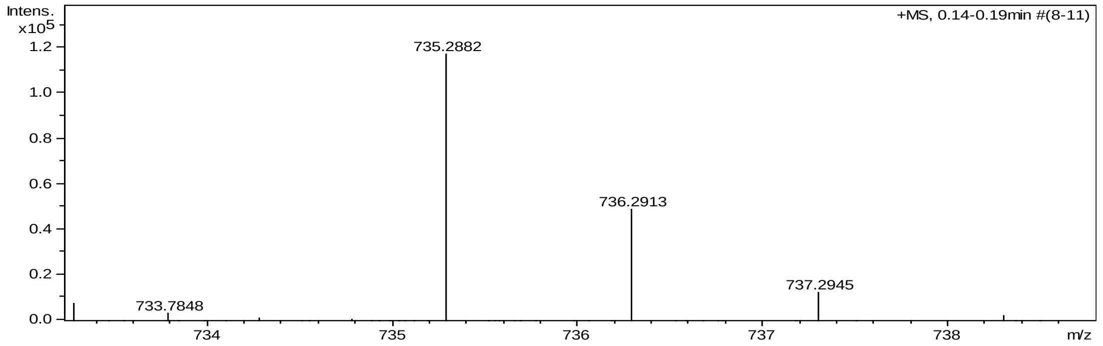
Intens.
x10^5
+MS, 0.14-0.19min #(8-11)
1.2
1.0
0.8
0.6
0.4
0.2
0.0
735.2882
736.2913
737.2945
733.7848
734
735
736
737
738
m/z

Pentamethyl *rel*-(4*R*,4a*R*,4b*S*,8a*S*)-8-cyclohexyl-4-phenyl-1-(*p*-tolyl)-4,4a,8,8a-tetrahydrocyclopenta[4,5]pyrrolo[2,3-*b*]pyridine-3,4b,5,6,7(1*H*)-pentacarboxylate (6d): yellow solid, 83%, m.p. 231-233 °C; ^{1}H NMR (400 MHz, $CDCl_3$) δ 7.80 (s, 1H, CH), 7.29 (s, 1H, ArH), 7.21-7.07 (m, 8H, ArH), 6.39 (d, *J* = 2.8 Hz, 1H, CH), 4.54 (t, *J* = 11.6 Hz, 1H, CH), 3.98 (s, 3H, OCH_3), 3.74 (s, 3H, OCH_3), 3.71 (dd, J_1 = 8.4 Hz, J_2 = 3.2 Hz, 1H, CH), 3.61 (s, 3H, OCH_3), 3.42 (s, 3H, OCH_3), 3.04-3.01 (m, 1H, CH), 3.01 (s, 3H, OCH_3), 2.39 (s, 3H, CH_3), 1.83-1.72 (m, 3H, CH_2), 1.66-1.50 (m, 2H, CH_2), 1.43-1.33 (m, 2H, CH_2), 1.24-1.16 (m, 2H, CH_2), 0.93-0.83 (m, 1H, CH_2) ppm. ^{13}C NMR (100 MHz, $CDCl_3$) δ 170.0, 167.7, 166.8, 166.2, 162.3, 161.8, 157.6, 143.6, 142.8, 139.5, 134.7, 130.6, 129.0, 127.9, 126.1, 120.0, 113.6, 109.9, 100.5, 80.2, 72.0, 58.6, 53.5, 52.9, 52.4, 51.4, 50.9, 50.8, 36.5, 31.3, 30.5, 25.8, 25.7, 24.8, 20.8 ppm. IR (KBr) υ: 3434, 2948, 2861, 1735, 1702, 1579, 1540, 1515, 1437, 1339, 1227, 1201, 1115, 1088, 1004, 981, 889, 819, 782, 757 cm^{-1}; MS (*m/z*): HRMS (ESI) Calcd. for $C_{39}H_{42}NaN_2O_{10}$ ($[M+Na]^+$): 721.2732, found: 721.2728.

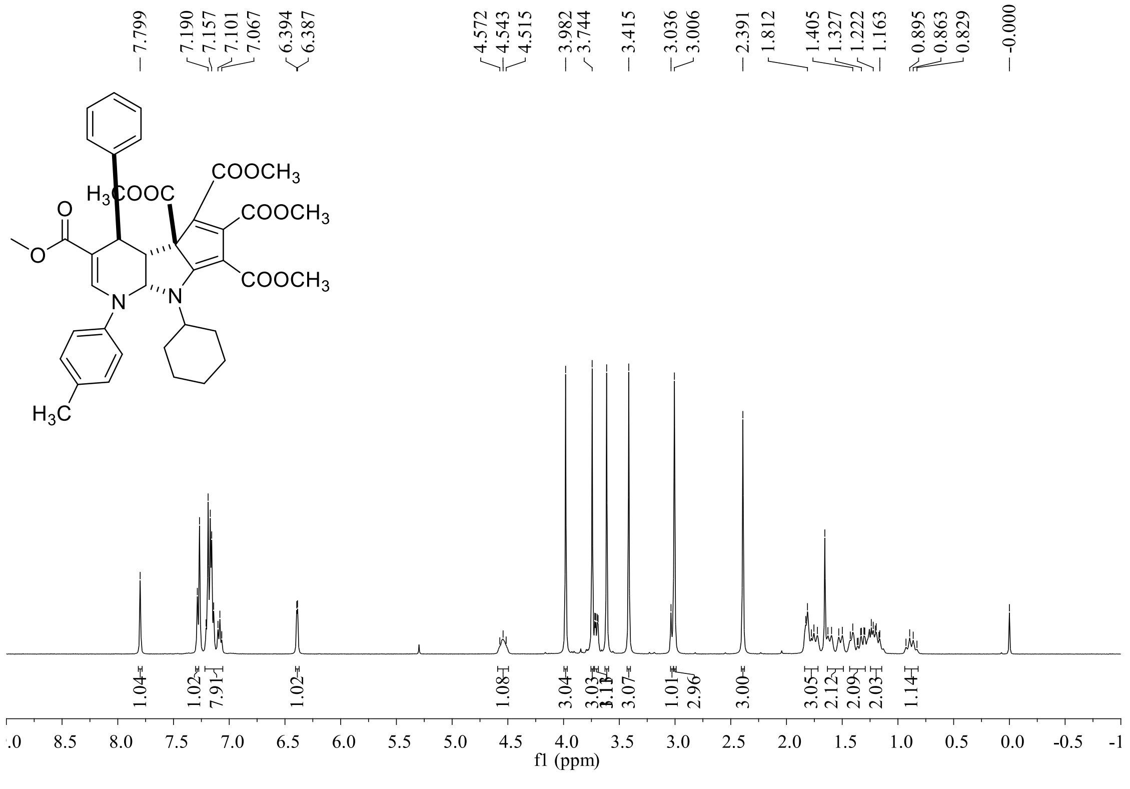

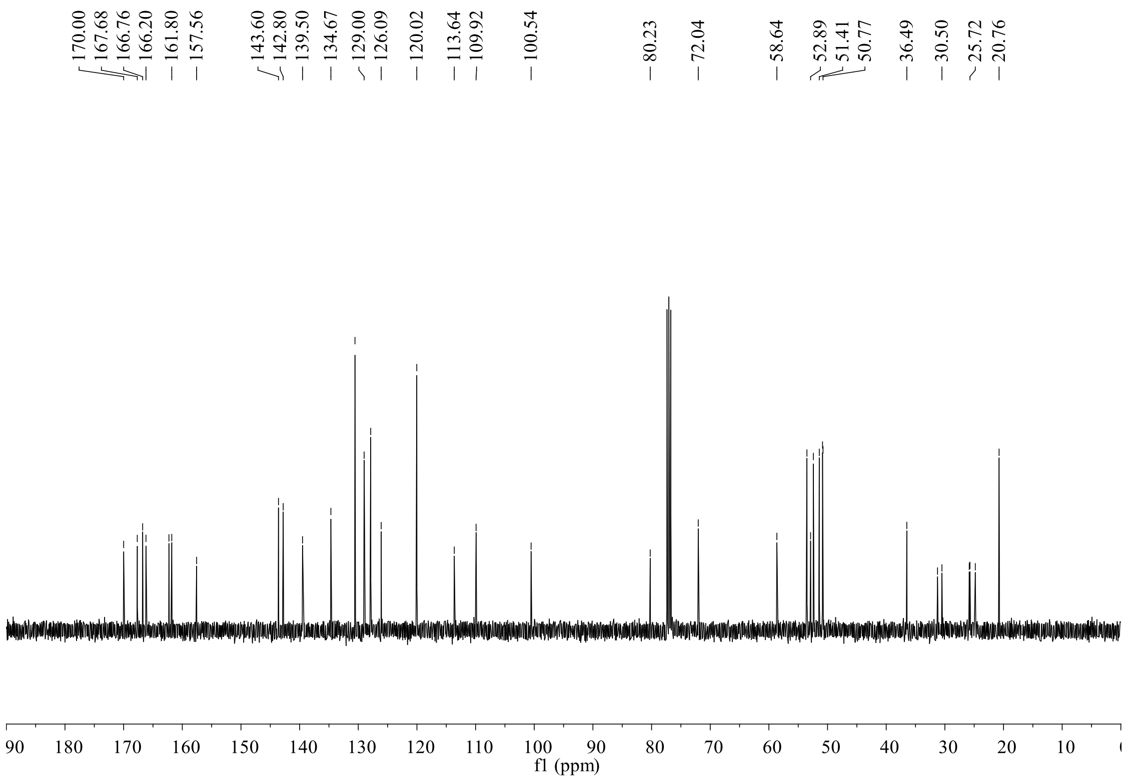
170.00
167.68
166.76
166.20
161.80
157.56
143.60
142.80
139.50
134.67
129.00
126.09
120.02
113.64
109.92
100.54
80.23
72.04
58.64
52.89
51.41
50.77
36.49
30.50
25.72
20.76
f1 (ppm)

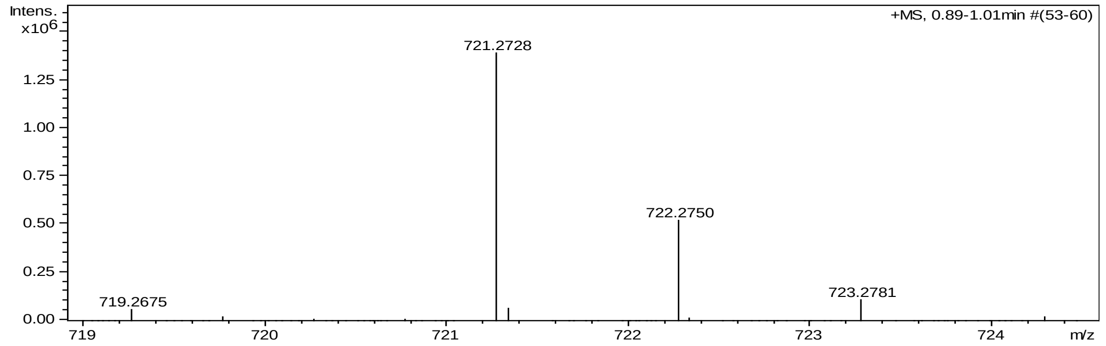
Intens.
x10^6
+MS, 0.89-1.01min #(53-60)
721.2728
722.2750
723.2781
719.2675
m/z

Pentamethyl *rel*-(4*R*,4a*R*,4b*S*,8a*S*)-1-(3-chlorophenyl)-8-cyclohexyl-4-phenyl-4,4a,8,8a-tetrahydrocyclopenta[4,5]pyrrolo[2,3-*b*]pyridine-3,4b,5,6,7(1*H*)-pentacarboxylate (6e): yellow solid, 84%, m.p. 201-203 °C; ^{1}H NMR (400 MHz, $CDCl_3$) δ 7.77 (s, 1H, CH), 7.42 (t, *J* = 8.0 Hz, 1H, ArH), 7.28 (s, 1H, ArH), 7.22-7.08 (m, 7H, ArH), 6.41 (s, 1H, CH), 4.54 (t, *J* = 12.4 Hz, 1H, CH), 3.98 (s, 3H, OCH_3), 3.75 (s, 3H, OCH_3), 3.72 (dd, J_1 = 8.4 Hz, J_2 = 3.2 Hz, 1H, CH), 3.63 (s, 3H, OCH_3), 3.45 (s, 3H, OCH_3), 3.06-3.03 (m, 1H, CH), 3.02 (s, 3H, OCH_3), 1.84-1.64 (m, 4H, CH_2), 1.56-1.46 (m, 2H, CH_2), 1.37-1.31 (m, 1H, CH_2), 1.26-1.15 (m, 2H, CH_2), 0.93-0.83 (m, 1H, CH_2) ppm. ^{13}C NMR (100 MHz, $CDCl_3$) δ 169.7, 167.7, 166.4, 166.1, 162.2, 161.7, 157.5, 146.0, 143.1, 137.7, 135.9, 131.1, 129.0, 128.0, 126.3, 124.7, 119.9, 117.6, 113.9, 112.2, 100.8, 79.9, 72.1, 58.6, 53.6, 52.9, 52.4, 51.5, 51.1, 50.8, 36.6, 31.3, 30.8, 25.9, 25.7, 24.9 ppm. IR (KBr) υ: 3434, 2948, 2860, 1735, 1699, 1590, 1538, 1482, 1436, 1337, 1229, 1200, 1115, 1087, 1006, 833, 788, 770, 751 cm^{-1}; MS (*m*/*z*): HRMS (ESI) Calcd. for $C_{38}H_{39}ClNaN_2O_{10}$ ([M+Na]$^{+}$): 741.2185, found: 741.2178.

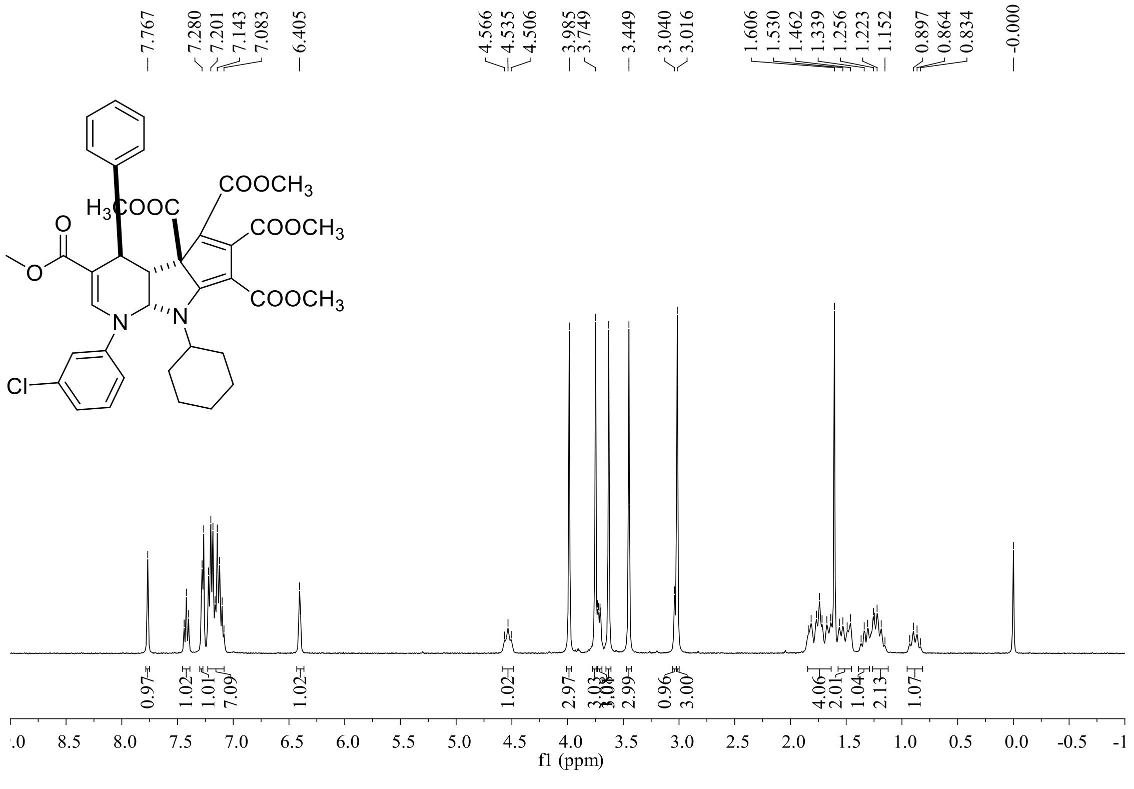

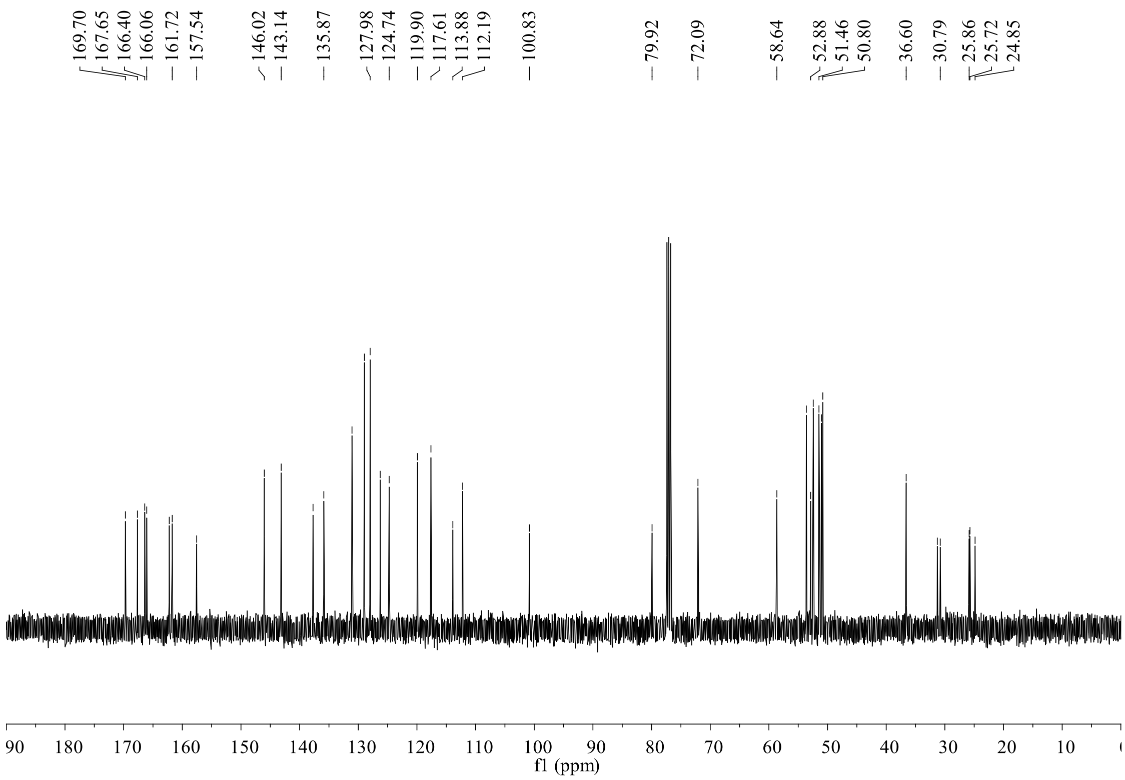
169.70
167.65
166.40
166.06
161.72
157.54
146.02
143.14
135.87
127.98
124.74
119.90
117.61
113.88
112.19
100.83
79.92
72.09
58.64
52.88
51.46
50.80
36.60
30.79
25.86
25.72
24.85
f1 (ppm)

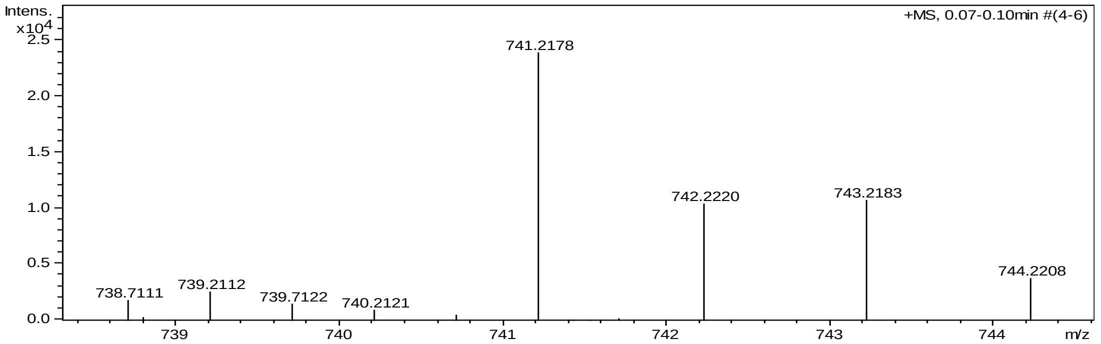
Intens.
x10^4
+MS, 0.07-0.10min #(4-6)
741.2178
742.2220
743.2183
744.2208
738.7111
739.2112
739.7122
740.2121
m/z

Pentamethyl *rel*-(4*R*,4a*R*,4b*S*,8a*S*)-8-cyclohexyl-4-phenyl-1-(*o*-tolyl)-4,4a,8,8a-tetrahydrocyclopenta[4,5]pyrrolo[2,3-*b*]pyridine-3,4b,5,6,7(1*H*)-pentacarboxylate (6f): yellow solid, 81%, m.p. 208-210 °C; ^{1}H NMR (400 MHz, $CDCl_3$) δ 7.62 (s, 1H, CH), 7.43 (d, *J* = 7.6 Hz, 1H, ArH), 7.36-7.32 (m, 2H, ArH), 7.29-7.27 (m, 1H, ArH), 7.23-7.20 (m, 4H, ArH), 7.12-7.09 (m, 1H, ArH), 5.98 (d, *J* = 2.4 Hz, 1H, CH), 4.16 (t, *J* = 12.0 Hz, 1H, CH), 3.98 (s, 3H, OCH_3), 3.83 (dd, J_1 = 9.2 Hz, J_2 = 3.2 Hz, 1H, CH), 3.75 (s, 3H, OCH_3), 3.57 (s, 3H, OCH_3), 3.38 (s, 3H, OCH_3), 3.06-3.03 (m, 1H, CH), 3.01 (s, 3H, OCH_3), 2.55 (s, 3H, CH_3), 2.10-2.02 (m, 1H, CH_2), 1.80-1.64 (m, 4H, CH_2), 1.57-1.54 (m, 1H, CH_2), 1.47-1.45 (m, 1H, CH_2), 1.23-1.07 (m, 3H, CH_2) ppm. ^{13}C NMR (100 MHz, $CDCl_3$) δ 169.9, 167.7, 166.8, 166.2, 162.1, 161.8, 157.5, 144.9, 143.8, 141.9, 134.0, 132.7, 129.0, 127.9, 127.5, 127.3, 126.3, 126.1, 114.1, 107.3, 101.7, 82.1, 77.4, 77.1, 76.8, 72.2, 59.0, 53.4, 52.4, 51.41, 51.35, 50.8, 50.7, 36.4, 30.8, 30.1, 25.8, 25.7, 24.4, 19.3 ppm. IR (KBr) υ: 3434, 2947, 2860, 1736, 1700, 1587, 1546, 1494, 1436, 1344, 1229, 1203, 1114, 1096, 1000, 952, 832, 793, 751 cm^{-1}; MS (*m/z*): HRMS (ESI) Calcd. for $C_{39}H_{42}NaN_2O_{10}$ ([M+Na]$^+$): 721.2732, found: 721.2725.

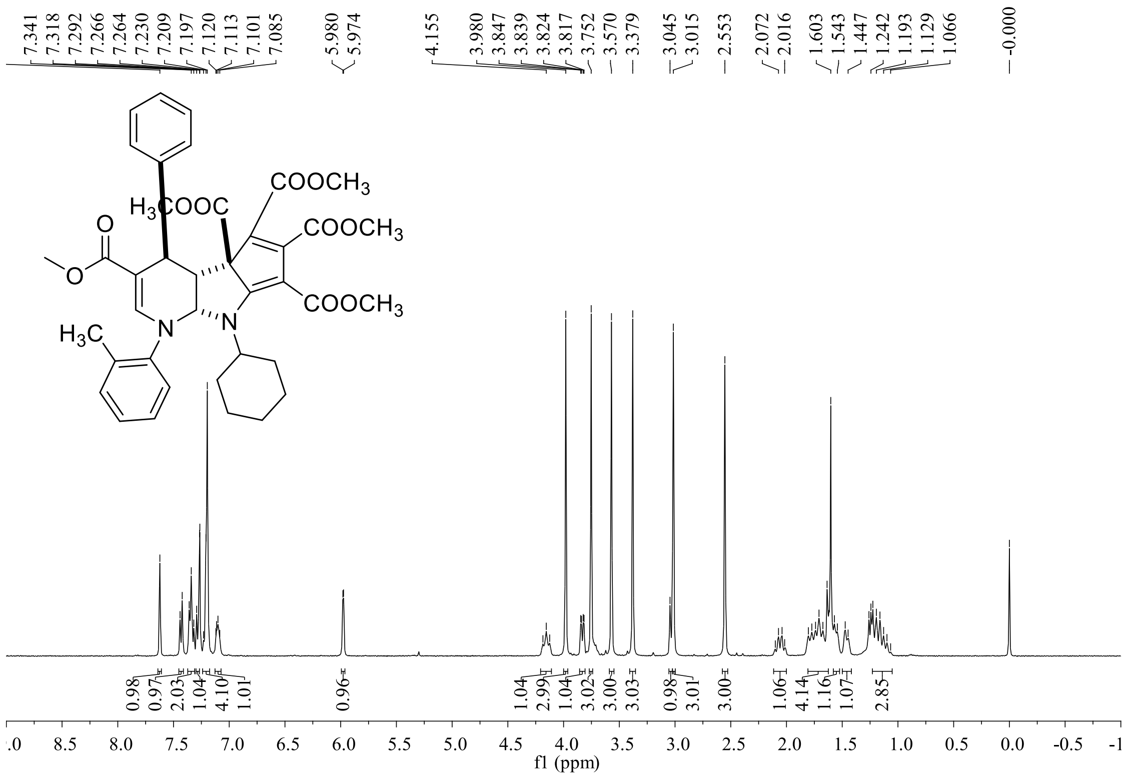

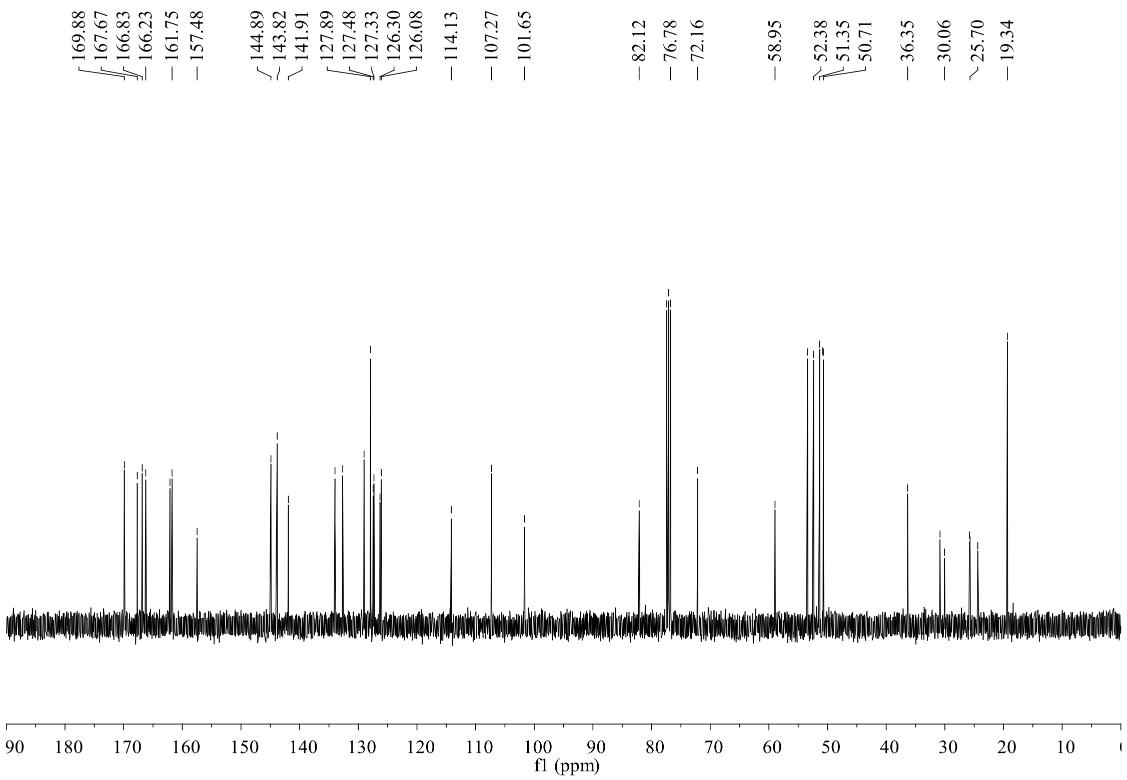
169.88
167.67
166.83
166.23
161.75
157.48
144.89
143.82
141.91
127.89
127.48
127.33
126.30
126.08
114.13
107.27
101.65
82.12
76.78
72.16
58.95
52.38
51.35
50.71
36.35
30.06
25.70
19.34
f1 (ppm)

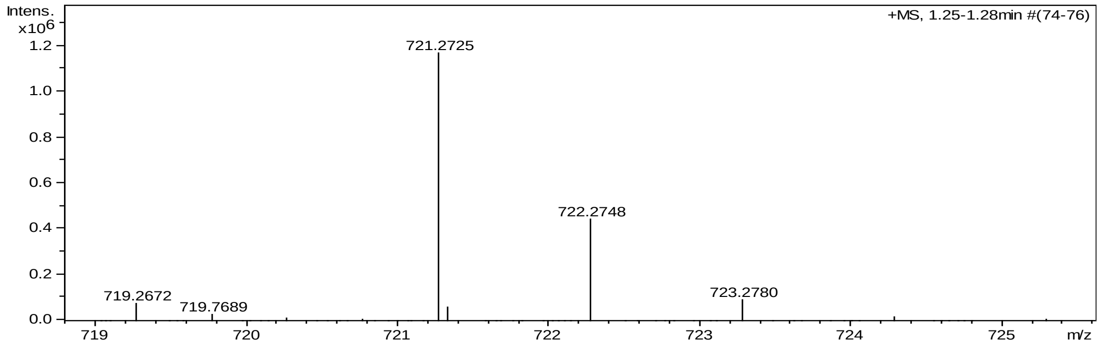
Intens.
x10^6
+MS, 1.25-1.28min #(74-76)
721.2725
722.2748
723.2780
719.2672
719.7689
m/z

Pentamethyl *rel*-(4*R*,4a*R*,4b*S*,8a*S*)-1-(4-bromophenyl)-8-cyclohexyl-4-(2-methoxyphenyl)-4,4a,8,8a-tetrahydrocyclopenta[4,5]pyrrolo[2,3-*b*]pyridine-3,4b,5,6,7(1*H*)-pentacarboxylate (6g): yellow solid, 88%, m.p. 233-235 °C; ^{1}H NMR (400 MHz, $CDCl_3$) δ 7.69 (s, 1H, CH), 7.58 (d, *J* = 8.4 Hz, 2H, ArH), 7.16 (d, *J* = 8.4 Hz, 2H, ArH), 7.11-7.07 (m, 2H, ArH), 6.81 (t, *J* = 7.2 Hz, 1H, ArH), 6.73 (d, *J* = 7.6 Hz, 1H, ArH), 6.39 (s, 1H, CH), 4.57 (t, *J* = 12.0 Hz, 1H, CH), 3.97 (s, 3H, OCH_3), 3.88 (dd, J_1 = 6.4 Hz, J_2 = 2.4 Hz, 1H, CH), 3.72 (s, 3H, OCH_3), 3.64 (s, 3H, OCH_3), 3.62 (s, 3H, OCH_3), 3.46 (s, 3H, OCH_3), 3.28-3.19 (m, 1H, CH), 3.01 (s, 3H, OCH_3), 1.87-1.67 (m, 4H, CH_2), 1.57-1.49 (m, 2H, CH_2), 1.43-1.34 (m, 1H, CH_2), 1.26-1.21 (m, 2H, CH_2), 0.96-0.86 (m, 1H, CH_2) ppm. ^{13}C NMR (100 MHz, $CDCl_3$) δ 169.7, 168.1, 166.6, 166.2, 162.3, 161.8, 157.6, 156.8, 144.0, 137.5, 132.9, 127.6, 120.7, 120.2, 117.0, 113.7, 112.2, 110.4, 100.1, 80.5, 72.5, 58.7, 55.1, 53.6, 52.3, 51.4, 51.0, 50.7, 31.4, 30.9, 25.9, 25.8, 25.0 ppm. IR (KBr) υ: 3448, 2948, 2857, 1736, 1706, 1655, 1584, 1544, 1435, 1399, 1341, 1312, 1230, 1155, 1113, 1075, 1002, 967, 923, 852, 823, 756 cm^{-1}; MS (*m/z*): HRMS (ESI) Calcd. for $C_{39}H_{41}BrNaN_2O_{11}$ ($[M+Na]^+$): 815.1786, found: 815.1767.

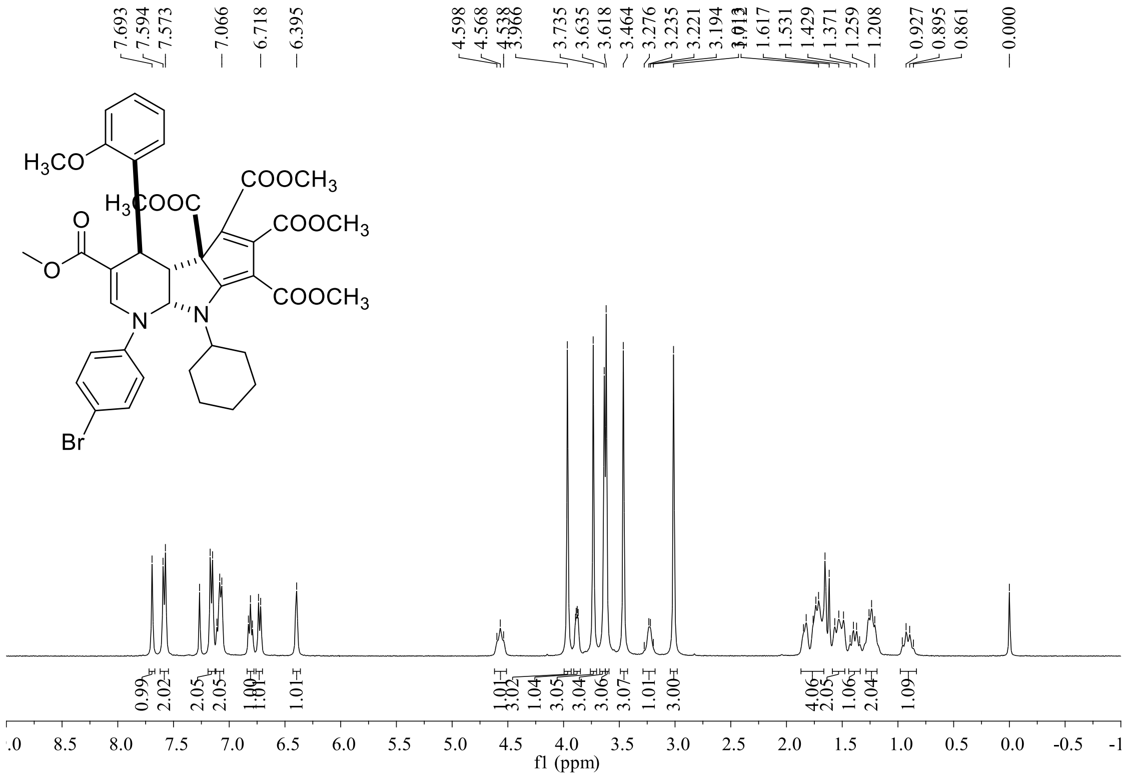

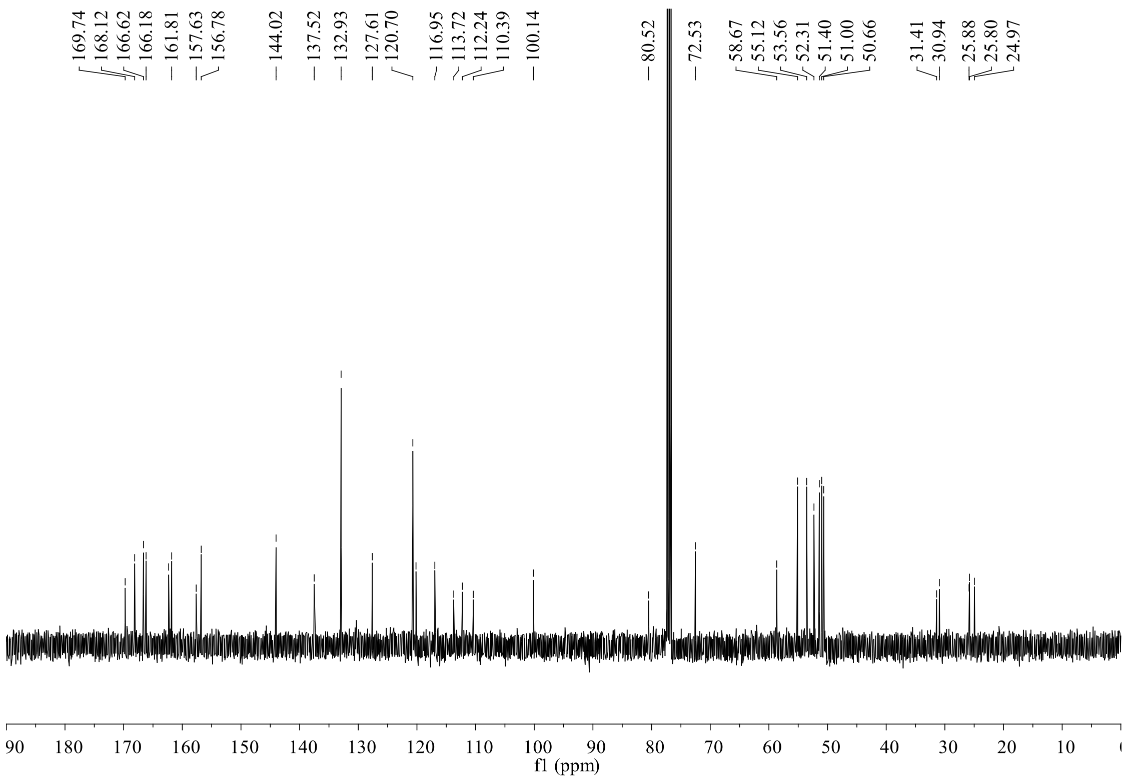
169.74
168.12
166.62
166.18
161.81
157.63
156.78
144.02
137.52
132.93
127.61
120.70
116.95
113.72
112.24
110.39
100.14
80.52
72.53
58.67
55.12
53.56
52.31
51.40
51.00
50.66
31.41
30.94
25.88
25.80
24.97
f1 (ppm)

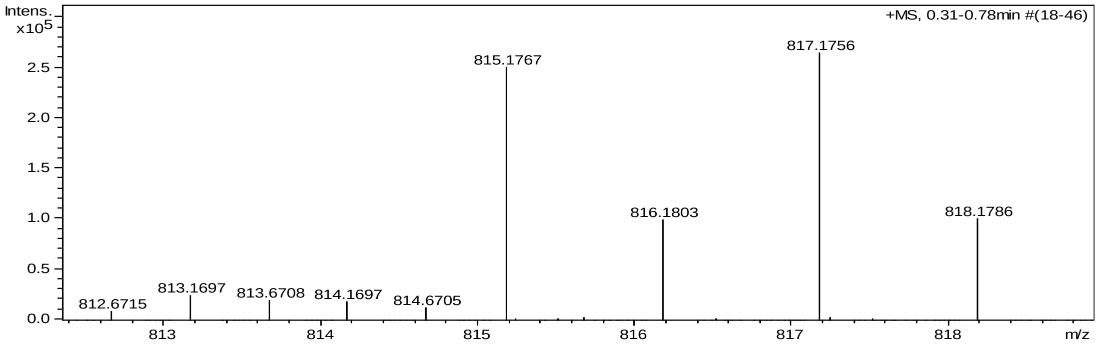
Intens.
x10^5
+MS, 0.31-0.78min #(18-46)
815.1767
817.1756
816.1803
818.1786
812.6715
813.1697
813.6708
814.1697
814.6705
m/z

Pentamethyl *rel*-(4*R*,4a*R*,4b*S*,8a*S*)-1-(4-bromophenyl)-8-cyclohexyl-4-(4-nitrophenyl)-4,4a,8,8a-tetrahydrocyclopenta[4,5]pyrrolo[2,3-*b*]pyridine-3,4b,5,6,7(1*H*)-pentacarboxylate (6h): yellow solid, 82%, m.p. 230-232 °C; ^{1}H NMR (400 MHz, $CDCl_3$) δ 8.08 (d, *J* = 8.4 Hz, 2H, ArH), 7.84 (s, 1H, CH), 7.63 (d, *J* = 8.8 Hz, 2H, ArH), 7.34 (d, *J* = 8.8 Hz, 2H, ArH), 7.19 (d, *J* = 8.8 Hz, 2H, ArH), 6.43 (d, *J* = 2.8 Hz, 1H, CH), 4.54 (t, *J* = 12.0 Hz, 1H, CH), 3.99 (s, 3H, OCH_3), 3.76 (s, 3H, OCH_3), 3.67-3.64 (m, 1H, CH), 3.63 (s, 3H, OCH_3), 3.46 (s, 3H, OCH_3), 3.13 (d, *J* = 8.4 Hz, 1H, CH), 3.07 (s, 3H, OCH_3), 1.87-1.79 (m, 1H, CH_2), 1.78-1.64 (m, 3H, CH_2), 1.58-1.51 (m, 1H, CH_2), 1.47-1.40 (m, 1H, CH_2), 1.29-1.17 (m, 3H, CH_2), 0.90-0.80 (m, 1H, CH_2) ppm. ^{13}C NMR (100 MHz, $CDCl_3$) δ 169.5, 167.3, 166.0, 165.8, 162.1, 161.5, 157.8, 151.5, 146.4, 143.9, 139.7, 133.3, 130.0, 123.2, 121.7, 118.4, 113.5, 109.2, 101.5, 79.5, 71.8, 58.9, 53.8, 52.6, 52.1, 51.6, 51.2, 51.0, 36.5, 31.4, 30.7, 25.8, 25.7, 24.8 ppm. IR (KBr) υ: 3430, 2950, 2854, 1738, 1699, 1583, 1542, 1435, 1401, 1345, 1232, 1206, 1111, 1045, 1002, 979, 831, 786 cm^{-1}; MS (*m*/*z*): HRMS (ESI) Calcd. for $C_{38}H_{38}BrNaN_3O_{12}$ ($[M+Na]^+$): 830.1531, found: 830.1512.

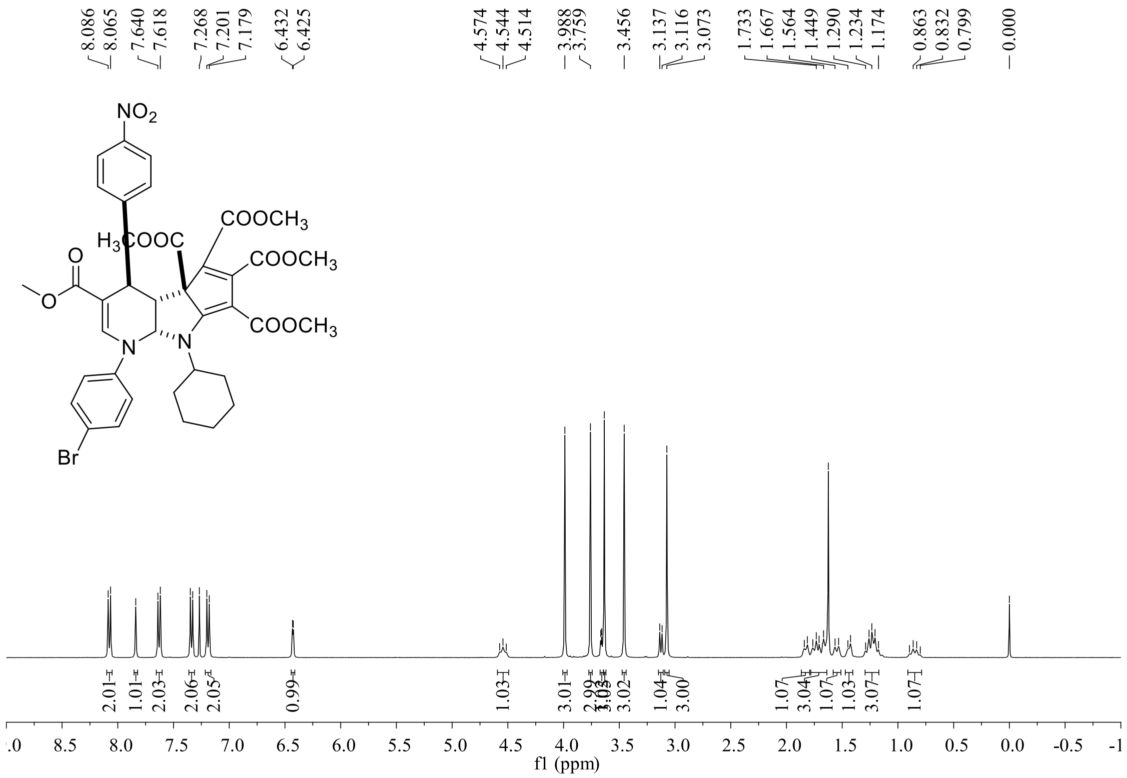

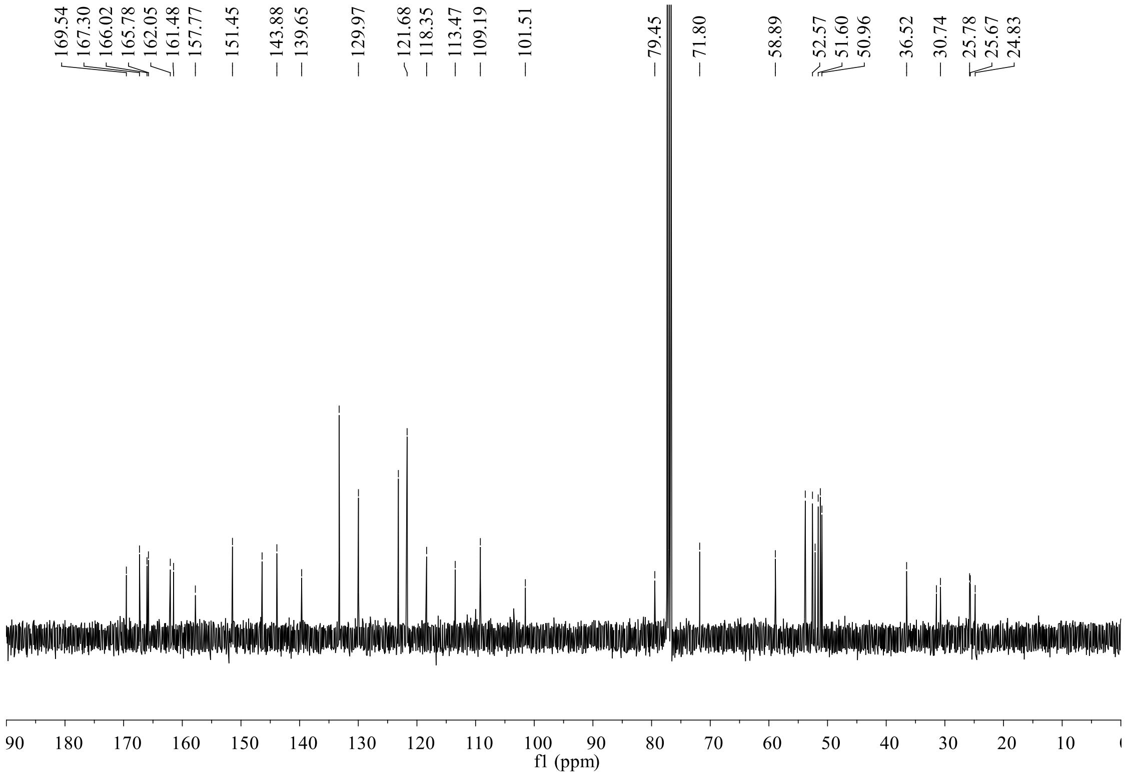
169.54
167.30
166.02
165.78
162.05
161.48
157.77
151.45
143.88
139.65
129.97
121.68
118.35
113.47
109.19
101.51
79.45
71.80
58.89
52.57
51.60
50.96
36.52
30.74
25.78
25.67
24.83
f1 (ppm)

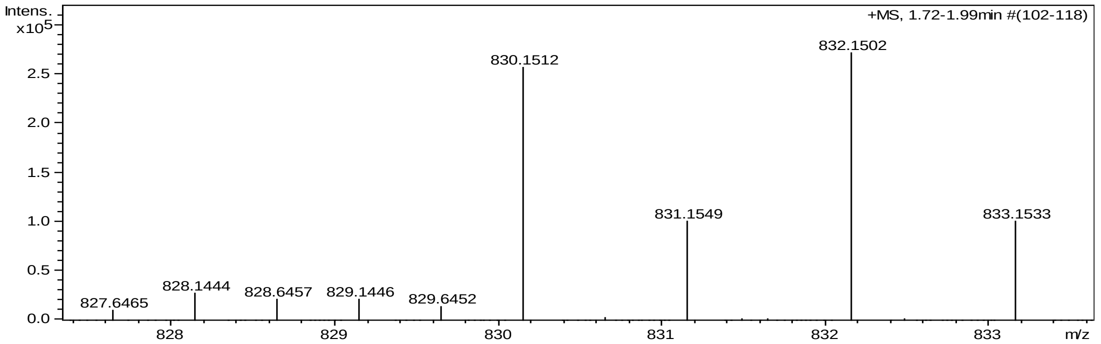
Intens.
x10^5
+MS, 1.72-1.99min #(102-118)
830.1512
832.1502
831.1549
833.1533
827.6465
828.1444
828.6457
829.1446
829.6452
m/z

4b,5,6,7-Tetraethyl 3-methyl *rel*-(4*R*,4a*R*,4b*S*,8a*S*)-1-benzyl-8-cyclohexyl-4-phenyl-4,4a,8,8a-tetrahydrocyclopenta[4,5]pyrrolo[2,3-*b*]pyridine-3,4b,5,6,7(1*H*)-pentacarboxylate (6i): yellow solid, 77%, m.p. 210-212 °C; ^{1}H NMR (400 MHz, $CDCl_3$) δ 7.51-7.40 (m, 6H, CH, ArH), 7.09-6.99 (m, 3H, ArH), 6.89 (t, *J* = 6.8 Hz, 2H, ArH), 5.80 (d, *J* = 2.8 Hz, 1H, CH), 4.96 (t, *J* = 11.6 Hz, 1H, CH), 4.56 (d, *J* = 15.2 Hz, 1H, CH_2), 4.51 (d, *J* = 15.2 Hz, 1H, CH_2), 4.47-4.39 (m, 1H, CH_2), 4.37-4.29 (m, 1H, CH_2), 4.23-4.14 (m, 2H, CH_2), 4.13-4.06 (m, 1H, CH_2), 3.98-3.90 (m, 1H, CH_2), 3.65-3.57 (m, 1H, CH_2), 3.37 (s, 3H, OCH_3), 3.22 (dd, J_1 = 8.8 Hz, J_2 = 3.2 Hz, 1H, CH), 3.18-3.12 (m, 1H, CH_2), 2.86 (t, *J* = 8.8 Hz, 1H, CH), 1.92-1.71 (m, 6H, CH_2), 1.63-1.54 (m, 1H, CH_2), 1.48-1.35 (m, 5H, CH_2, CH_3), 1.31 (t, *J* = 7.2 Hz, 3H, CH_3), 1.21-1.15 (m, 1H, CH_2), 1.07 (t, *J* = 7.2 Hz, 3H, CH_3), 0.76 (t, *J* = 7.2 Hz, 3H, CH_3) ppm. ^{13}C NMR (100 MHz, $CDCl_3$) δ 170.4, 166.9, 166.7, 165.7, 162.2, 161.4, 144.1, 141.8, 136.9, 129.2, 128.9, 128.4, 127.8, 127.6, 125.8, 114.3, 108.8, 100.4, 81.2, 77.3, 77.0, 76.7, 72.1, 62.1, 61.1, 60.2, 60.0, 59.4, 58.6, 52.8, 50.6, 36.1, 32.1, 31.4, 26.1, 25.6, 14.11, 14.05, 13.8, 13.6 ppm. IR (KBr) υ: 3435, 2940, 2859, 1729, 1702, 1576, 1538, 1448, 1367, 1330, 1229, 1188, 1108, 1080, 1027, 988, 897, 853, 761 cm^{-1}; MS (*m*/*z*): HRMS (ESI) Calcd. for $C_{43}H_{50}NaN_2O_{10}$ ([M+Na]$^{+}$): 777.3358, found: 777.3350.

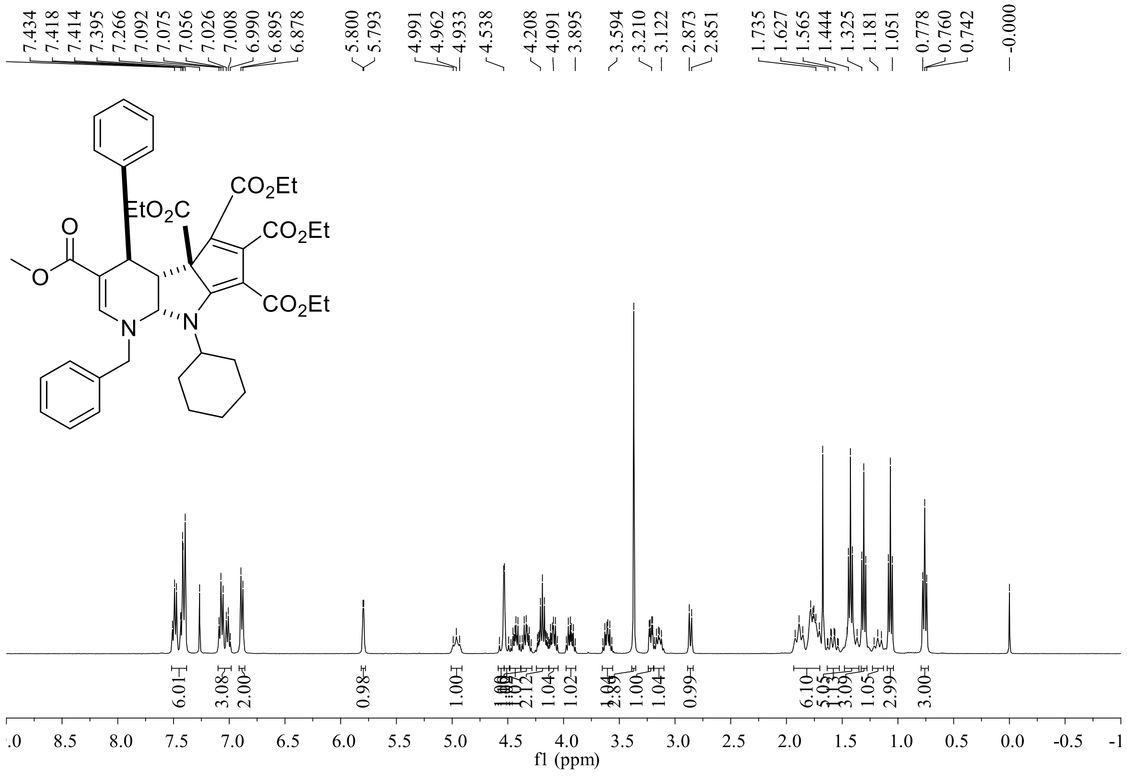

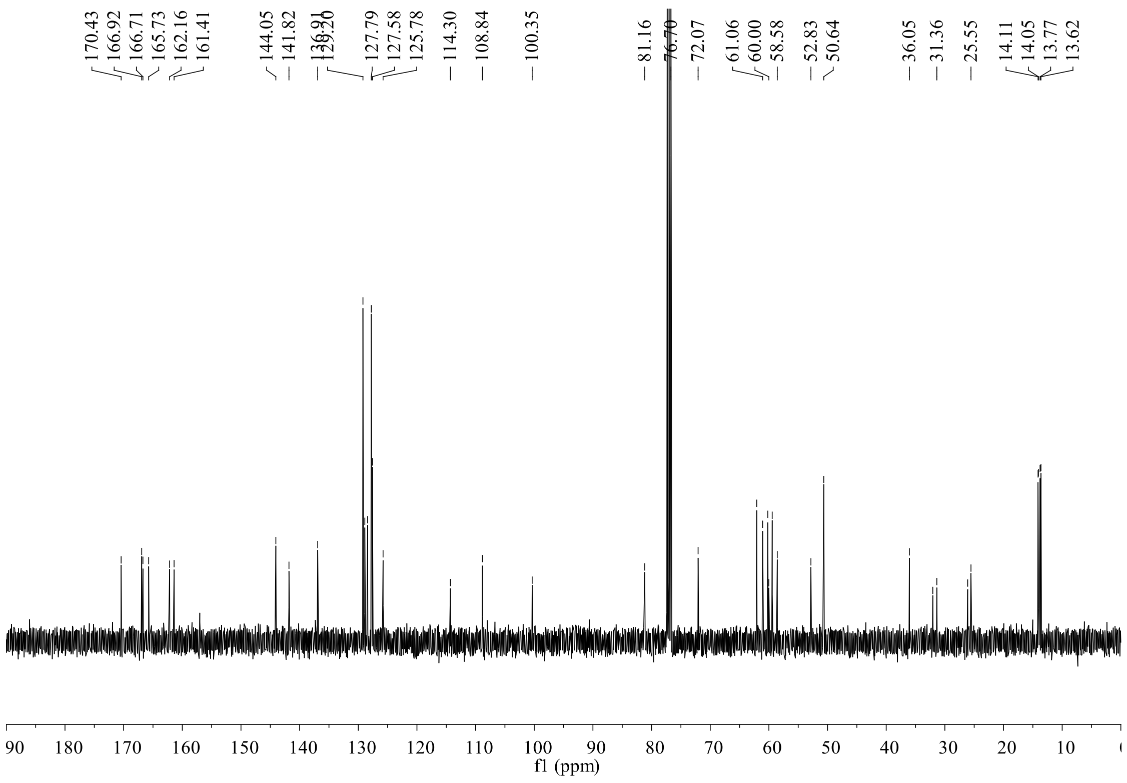
170.43
166.92
166.71
165.73
162.16
161.41
144.05
141.82
136.91
129.20
127.79
127.58
125.78
114.30
108.84
100.35
81.16
76.70
72.07
61.06
60.00
58.58
52.83
50.64
36.05
31.36
25.55
14.11
14.05
13.77
13.62
f1 (ppm)

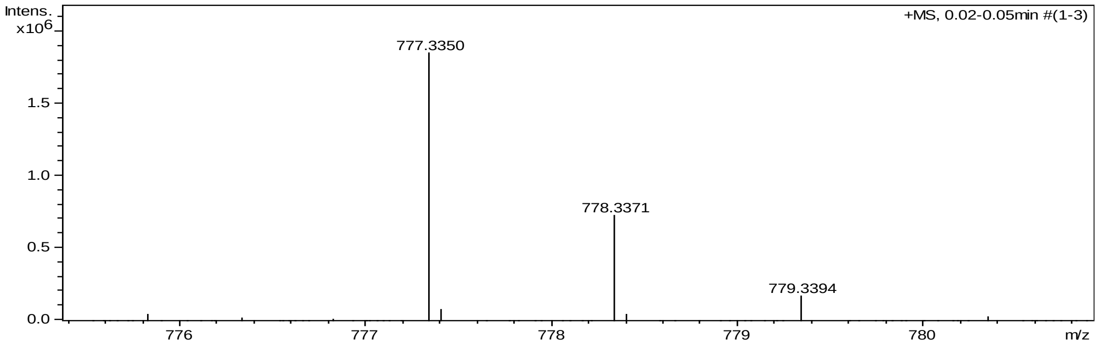
Intens.
x10^6
+MS, 0.02-0.05min #(1-3)
777.3350
778.3371
779.3394
m/z

Pentamethyl *rel*-(4*R*,4a*R*,4b*S*,8a*S*)-1-benzyl-8-(*tert*-butyl)-4-phenyl-4,4a,8,8a-tetrahydrocyclopenta[4,5]pyrrolo[2,3-*b*]pyridine-3,4b,5,6,7(1*H*)-pentacarboxylate (6j): yellow solid, 70%, m.p. 205-207 °C; ^{1}H NMR (400 MHz, $CDCl_3$) δ 7.53-7.41 (m, 5H, CH, ArH), 7.36 (s, 1H, ArH), 6.98-6.97 (m, 3H, ArH), 6.51 (d, *J* = 6.4 Hz, 2H, ArH), 5.90 (s, 1H, CH), 4.56 (d, *J* = 14.4 Hz, 1H, CH_2), 4.46 (d, *J* = 14.4 Hz, 1H, CH_2), 3.96 (s, 3H, OCH_3), 3.74 (s, 3H, OCH_3), 3.61 (s, 3H, OCH_3), 3.40 (s, 3H, OCH_3), 3.10-3.07 (m, 1H, CH), 2.98 (s, 3H, OCH_3), 2.68 (d, *J* = 7.6 Hz, 1H, CH), 1.53 (s, 9H, CH_3) ppm. ^{13}C NMR (100 MHz, $CDCl_3$) δ 170.5, 167.9, 166.4, 166.1, 162.7, 161.6, 157.1, 144.2, 140.9, 136.8, 129.3, 128.6, 128.5, 128.4, 127.5, 125.7, 115.3, 111.7, 105.4, 83.5, 77.4, 77.1, 76.7, 72.8, 59.6, 58.5, 53.2, 52.5, 51.4, 50.9, 50.7, 49.7, 35.7, 29.9 ppm. IR (KBr) υ: 3434, 2952, 2360, 1735, 1702, 1642, 1595, 1533, 1435, 1395, 1358, 1233, 1177, 1108, 1080, 1000, 978, 915, 851, 793, 761 cm^{-1}; MS (*m/z*): HRMS (ESI) Calcd. for $C_{37}H_{40}NaN_2O_{10}$ ($[M+Na]^+$): 695.2575, found: 695.2570.

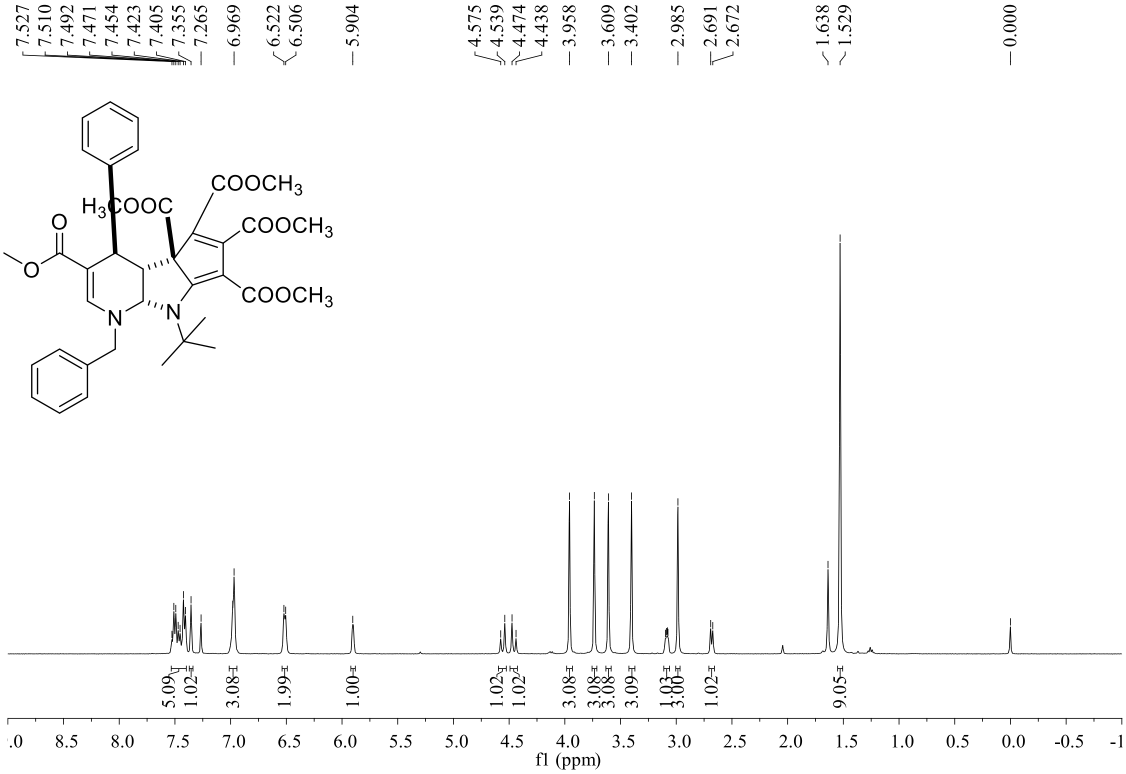

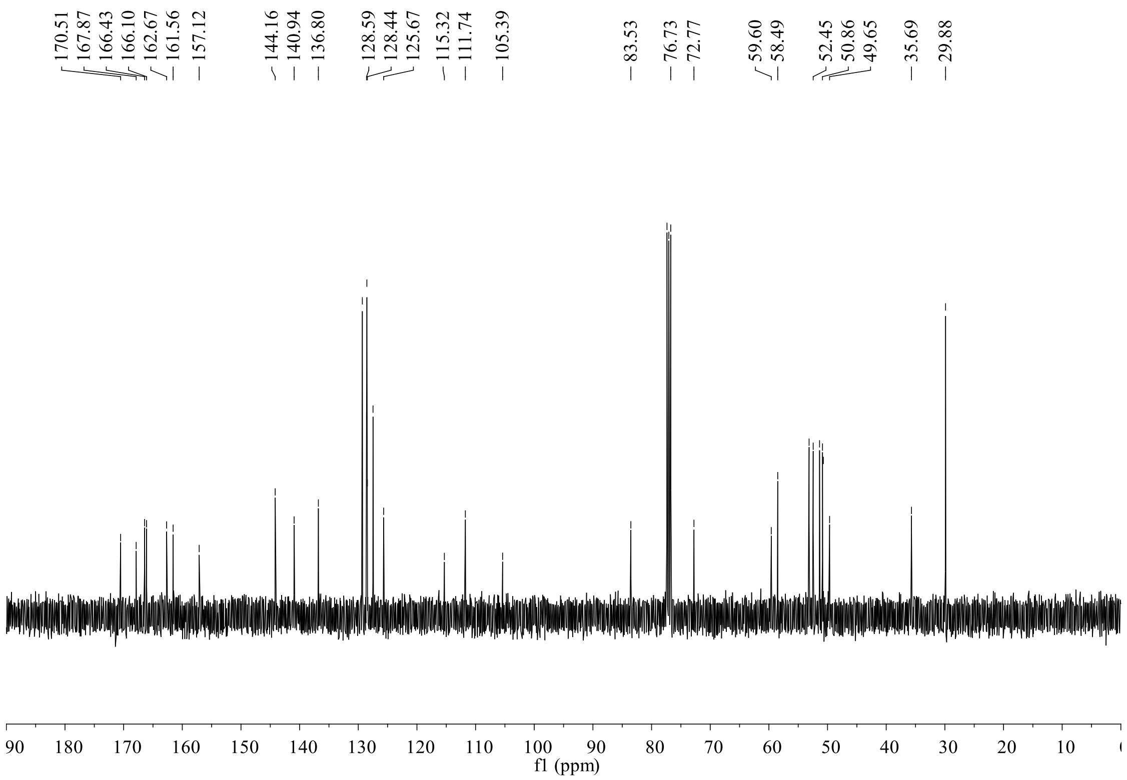
170.51
167.87
166.43
166.10
162.67
161.56
157.12
144.16
140.94
136.80
128.59
128.44
125.67
115.32
111.74
105.39
83.53
76.73
72.77
59.60
58.49
52.45
50.86
49.65
35.69
29.88
f1 (ppm)

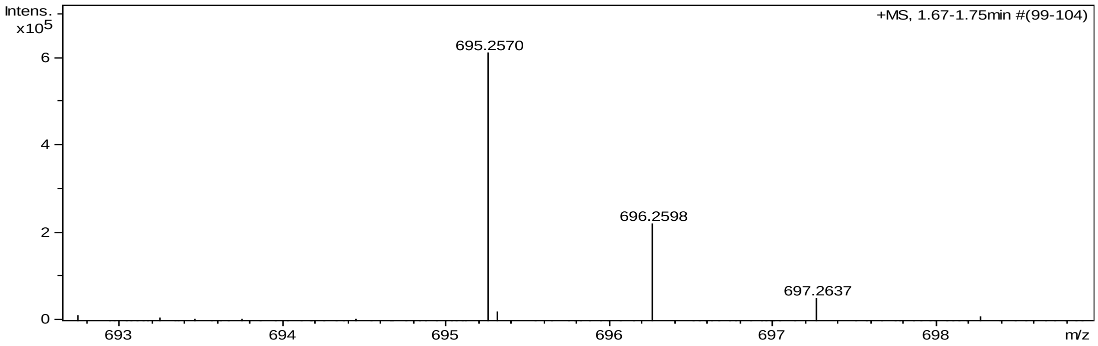
Intens.
x10^5
+MS, 1.67-1.75min #(99-104)
695.2570
696.2598
697.2637
m/z

Pentamethyl *rel*-(4*R*,4a*R*,4b*S*,8a*S*)-1,8-dibenzyl-4-phenyl-4,4a,8,8a-tetrahydrocyclopenta[4,5]pyrrolo[2,3-*b*]pyridine-3,4b,5,6,7(1*H*)-pentacarboxylate (6k): yellow solid, 58%, m.p. 218-220 °C; ^{1}H NMR (400 MHz, $CDCl_3$) δ 7.42-7.31 (m, 7H, CH, ArH), 7.19-7.14 (m, 4H, ArH), 7.11-7.03 (m, 5H, ArH), 6.08 (d, *J* = 16.0 Hz, 1H, CH_2), 5.50 (d, *J* = 2.8 Hz, 1H, CH), 4.51 (d, *J* = 16.0 Hz, 1H, CH_2), 4.39 (d, *J* = 15.2 Hz, 1H, CH_2), 4.04-3.96 (m, 4H, CH_2, CH_3) 3.68 (s, 3H, OCH_3), 3.58 (s, 3H, OCH_3), 3.48 (dd, J_1 = 8.8 Hz, J_2 = 3.2 Hz, 1H, CH), 3.38 (s, 3H, OCH_3), 3.01-2.99 (m, 1H, CH), 2.98 (s, 3H, OCH_3) ppm. ^{13}C NMR (100 MHz, $CDCl_3$) δ 170.8, 167.0, 166.7, 166.0, 162.7, 161.9, 156.9, 143.7, 141.4, 137.0, 136.6, 129.1, 129.0, 128.9, 128.3, 127.9, 127.1, 126.6, 126.0, 113.6, 107.1, 99.5, 80.9, 77.3, 77.0, 76.7, 72.4, 59.1, 53.7, 53.4, 52.5, 51.4, 50.8, 50.7, 48.5, 35.9 ppm. IR (KBr) υ: 3390, 2949, 1738, 1696, 1585, 1548, 1434, 1383, 1346, 1230, 1116, 1094, 1075, 1011, 988, 847, 810, 795, 780, 763 cm^{-1}; MS (*m*/*z*): HRMS (ESI) Calcd. for $C_{40}H_{38}NaN_2O_{10}$ ([M+Na]$^+$): 729.2419, found: 729.2414.

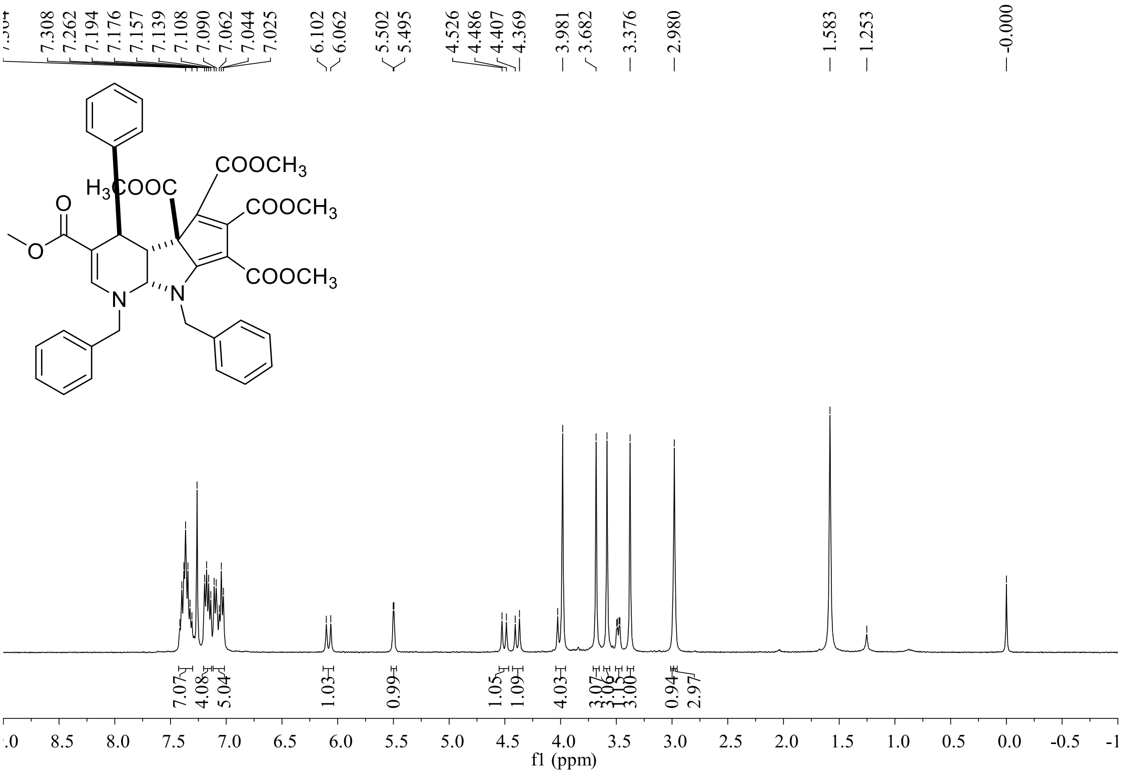

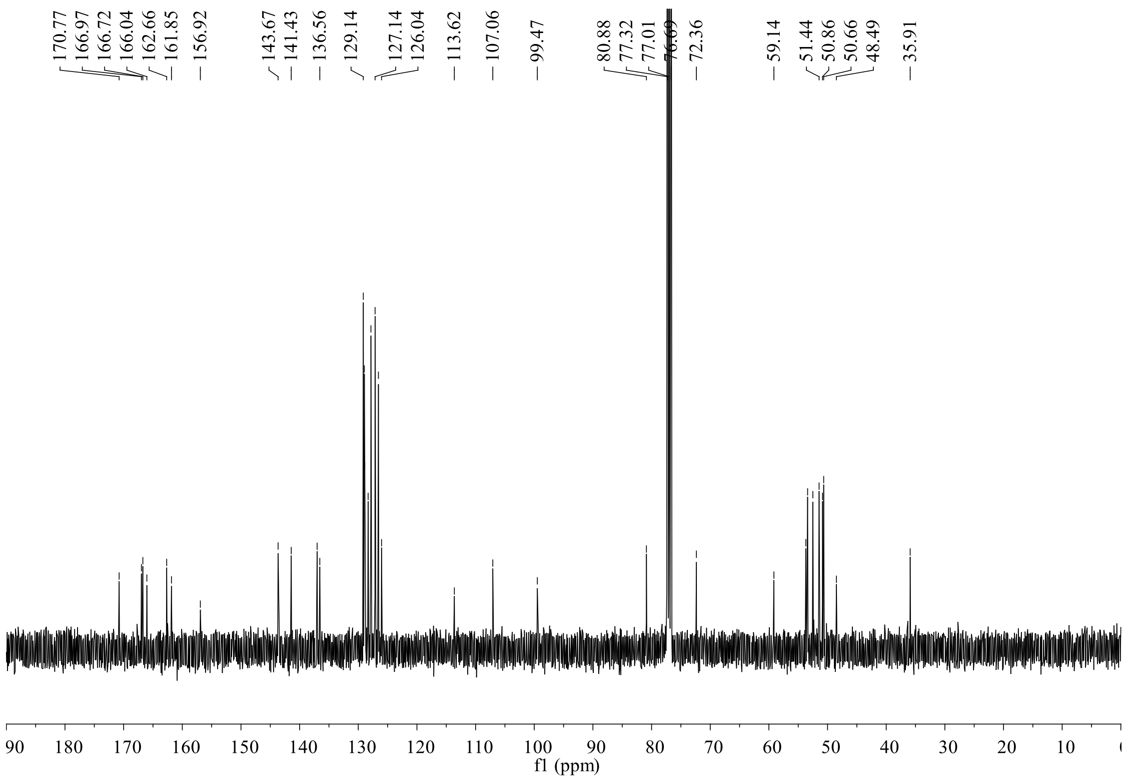
170.77
166.97
166.72
166.04
162.66
161.85
156.92
143.67
141.43
136.56
129.14
127.14
126.04
113.62
107.06
99.47
80.88
77.32
77.01
72.36
59.14
51.44
50.86
50.66
48.49
35.91
180
170
160
150
140
130
120
110
100
90
80
70
60
50
40
30
20
10
f1 (ppm)

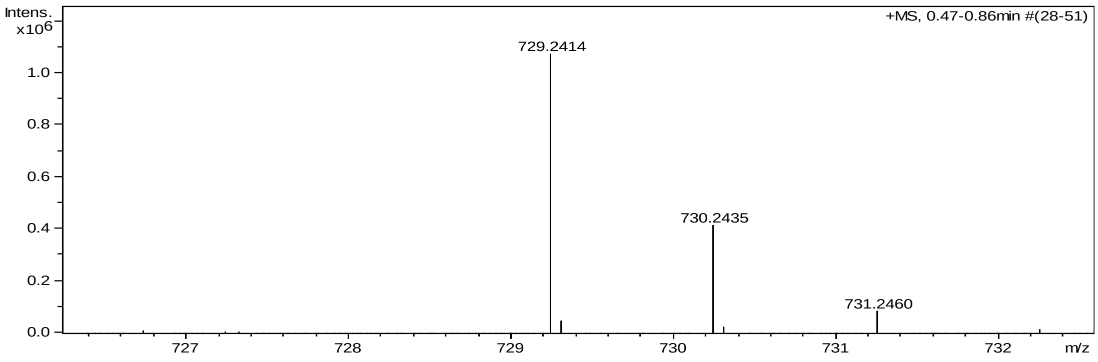
Intens.
x10^6
+MS, 0.47-0.86min #(28-51)
729.2414
730.2435
731.2460
1.0
0.8
0.6
0.4
0.2
0.0
727
728
729
730
731
732
m/z
